# Single-Cell Sequencing of Developing Human Gut Reveals Transcriptional Links to Childhood Crohn’s Disease

**DOI:** 10.1016/j.devcel.2020.11.010

**Published:** 2020-12-21

**Authors:** Rasa Elmentaite, Alexander D.B. Ross, Kenny Roberts, Kylie R. James, Daniel Ortmann, Tomás Gomes, Komal Nayak, Liz Tuck, Sophie Pritchard, Omer Ali Bayraktar, Robert Heuschkel, Ludovic Vallier, Sarah A. Teichmann, Matthias Zilbauer

**Affiliations:** 1 Wellcome Sanger Institute, Wellcome Genome Campus, Hinxton CB10 1SA, UK; 2 Wellcome Trust, MRC Cambridge Stem Cell Institute, University of Cambridge, Cambridge CB2 0SZ, UK; 3 Department of Surgery, University of Cambridge, Cambridge CB2 0QQ, UK; 4 Department of Paediatrics, University of Cambridge, Cambridge CB2 0QQ, UK; 5 Theory of Condensed Matter, Cavendish Laboratory, Department of Physics, University of Cambridge, Cambridge CB3 0HE, UK; 6 European Molecular Biology Laboratory, European Bioinformatics Institute (EBI), Wellcome Genome Campus, Hinxton CB10 1SA, UK; 7 Department of Paediatric Gastroenterology, Hepatology and Nutrition, Cambridge University Hospitals Trust, Cambridge CB2 0QQ, UK

**Keywords:** single-cell RNA sequencing, villus formation, inflammatory bowel disease, human fetal gut development, intestinal stem cells, pediatric Crohn's disease, intestinal organoids

## Abstract

Human gut development requires the orchestrated interaction of differentiating cell types. Here, we generate an in-depth single-cell map of the developing human intestine at 6–10 weeks post-conception. Our analysis reveals the transcriptional profile of cycling epithelial precursor cells; distinct from LGR5-expressing cells. We propose that these cells may contribute to differentiated cell subsets via the generation of LGR5-expressing stem cells and receive signals from surrounding mesenchymal cells. Furthermore, we draw parallels between the transcriptomes of *ex vivo* tissues and *in vitro* fetal organoids, revealing the maturation of organoid cultures in a dish. Lastly, we compare scRNA-seq profiles from pediatric Crohn’s disease epithelium alongside matched healthy controls to reveal disease-associated changes in the epithelial composition. Contrasting these with the fetal profiles reveals the re-activation of fetal transcription factors in Crohn’s disease. Our study provides a resource available at www.gutcellatlas.org, and underscores the importance of unraveling fetal development in understanding disease.

## Highlights

• Single-cell RNA-seq map of the developing and pediatric human intestine
• Cycling BEX5+ epithelial precursors are distinct from adult LGR5+ stem cells
• Human fetal intestinal organoids mature in culture
• Fetal transcription factors are reactivated in the Crohn’s disease epithelium

## Graphical Abstract

**Figure:**
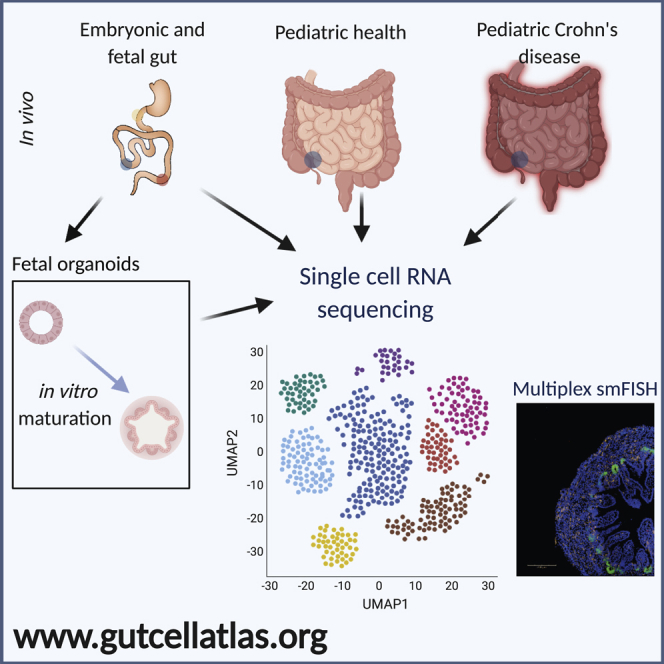

## Teaser

Elmentaite and Ross et al. generated a detailed single-cell RNA-seq map of the developing human intestine. Data analyses revealed the presence of an early intestinal epithelial precursor cell and the re-activation of fetal transcription factors in the intestinal epithelium of children diagnosed with Crohn’s disease. The study provides a unique resource accessible to all researchers at www.gutcellatlas.org.

## Introduction

Development of the human intestine is a highly complex process that requires synergy between a wide range of cell types. Subtle differences between humans and mice (Chin et al., 2017; Yanai et al., 2017) combined with a limited access to human fetal and embryonic tissues, has rendered our understanding of these processes in humans rudimentary. Importantly, environmentally triggered alterations in early development have been implicated in a range of immune-mediated pathologies, including inflammatory bowel diseases (IBD) (Sonntag et al., 2007; Cilieborg et al., 2012; Dupaul-Chicoine et al., 2013; Kraiczy et al., 2016). Furthermore, a number of studies have reported a link between early fetal intestinal epithelial cell dynamics and IBD (Kraiczy et al., 2016; Yui et al., 2018; Wang et al., 2019), suggesting that fetal-like transcriptional programs may re-appear in the intestinal epithelium of IBD patients. Hence, deciphering physiological intestinal development is a critical step toward prevention and treatment of such conditions.

The human intestinal tract develops from the endodermal germ cell layer of the embryo, beginning with the formation of a simple tube at 3–4 weeks post-conception (PCW). Prior to villus formation, the intestinal epithelium, forming the innermost lining of the gut tube, is pseudostratified and is globally proliferative (Grosse et al., 2011). By the end of the first trimester (12 PCW), regionalization of the intestinal tube occurs and a crypt-villus axis starts to appear. While little is known about the villus formation in humans, two mechanisms have been proposed in model organisms: mesenchymal clustering in mice and the force generated by smooth muscle in chickens (Karlsson et al., 2000; Walton et al., 2012, 2016a, 2016b; Shyer et al., 2013). While there are significant differences between the two models, both employ similar signaling, including gradients of hedgehog (HH), PDGF, and bone morphogenetic protein (BMP) ligands (Kolterud et al., 2009; Korinek et al., 1998; Kurahashi et al., 2008; Madison et al., 2005; Geske et al., 2008; Shyer et al., 2015).

In the adult intestine, LGR5 is a marker of stem cells that reside at the bottom of intestinal crypts and give rise to all epithelial cell subsets (Barker et al., 2007). The ability to generate self-organizing intestinal epithelial organoids from fetal gut epithelium as early as 8–10 PCW implies the presence of these LGR5+ stem cells (Fordham et al., 2013). Indeed, the use of organoid models as tools to investigate early fetal intestinal development has been demonstrated previously (Kraiczy et al., 2019). Nevertheless, the cross-talk between epithelial cell subsets and other mucosal cell types, as well as cell lineage trajectories, remain unknown. Recent studies have used single-cell RNA sequencing (scRNA-seq) to interrogate intestinal regional specification and immune system development in mice and humans (Gao et al., 2018; Nowotschin et al., 2019; Czerwinski et al., 2020; Yu et al., 2020; Li et al., 2019; Schreurs et al., 2019). However, human villus formation and epithelial dynamics have not yet been explored in detail.

In this study, we performed single-cell transcriptional profiling of human embryonic and early fetal gut samples obtained from nine human embryos spanning between ages 6 and 10 PCW. Additionally, we profiled mucosal biopsies from the small bowel of healthy children aged between 4 and 12 years and a group of children newly diagnosed with Crohn’s disease (CD)—a common form of IBD. In total, we generated single-cell transcriptomes of ~90,000 primary human intestinal cells, providing a rich resource and a detailed roadmap. Using these data, as well as scRNA-seq profiles of human fetal gut derived organoids, we describe embryonic and fetal epithelium composition, trace their differentiation dynamics and signaling partners, and provide links to regenerating CD epithelium.

## Results

### Single-Cell Map of the Human Embryonic, Fetal and Pediatric Gut

**Figure 1:**
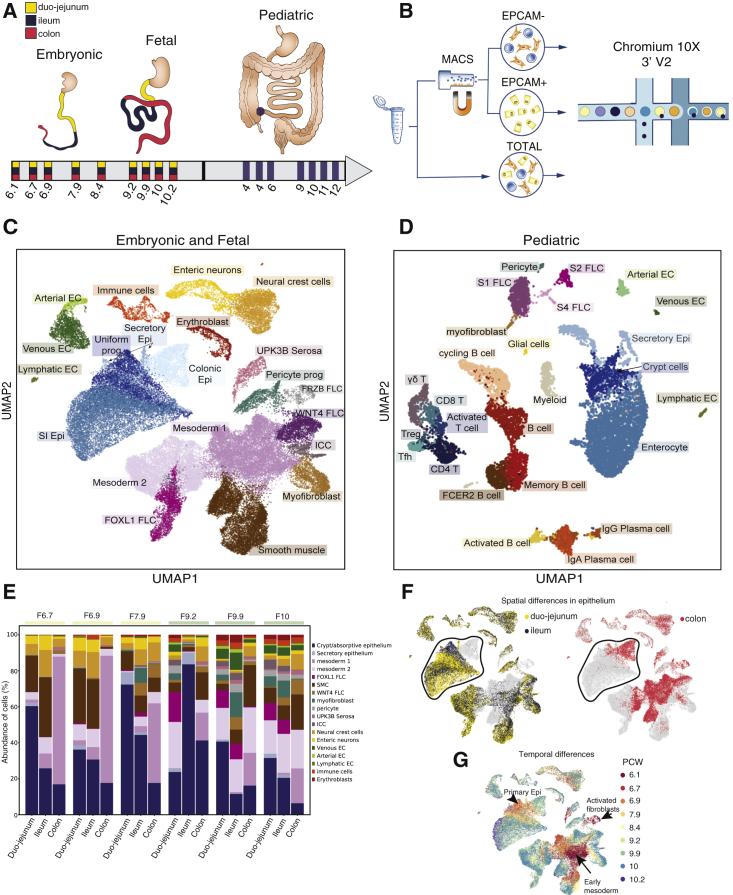
Single-Cell Profiling of Embryonic, Fetal and Pediatric Gut (A) Schematic illustration of experimental design. Blue circles mark biopsy location (i.e., terminal ileum). (B) Tissue dissociation and single-cell sequencing strategy. (C and D) (C) and (D) UMAP projection of embryonic/fetal (n = 9 donors) and childhood/adolescence (n = 8 donors) scRNA-seq samples, respectively. (E) Changes in embryonic/fetal cell type abundance (% of cells) at different developmental time points grouped by intestinal regions. Time point annotation colored in yellow and green are embryonic and fetal, respectively. Crypt/absorptive epithelium are SI, colonic epithelium, and uniform progenitors grouped together. (F and G) UMAP plots colored by (F) gut region of the embryonic cells, (G) post-conception week of embryonic and fetal cells as in (C). Circled populations in F are epithelial cells. EC, endothelial cell; FLC, fibroblasts; Epi, epithelium; SI, small intestinal; prog, progenitors; ICC, interstitial cells of Cajal. See also Figure S1; Table S1.

Human embryos with a post-conceptional age ranging from 6 to 10 weeks were dissected to remove the intestinal tube, which was divided into proximal small bowel (duodenum and jejunum), distal small bowel (ileum), and large bowel (colon). Additionally, we obtained small bowel (i.e., terminal ileum) mucosal biopsies from healthy children aged between 4 and 12 years (Figure 1A; Table S1). Tissue samples were dissociated into single-cell suspensions and processed using the 10x Genomics Chromium workflow (STAR Methods). In a subset of samples, the intestinal epithelial cell fraction was enriched by performing magnetic bead sorting for epithelial cell adhesion molecule (EPCAM) (Figure 1B; Table S1). In total, 62,854 fetal (n = 34) and 11,302 pediatric terminal ileal (n = 8) cells passed quality control and doublet exclusion criteria (Figure S1; Table S1).

**Figure 2:**
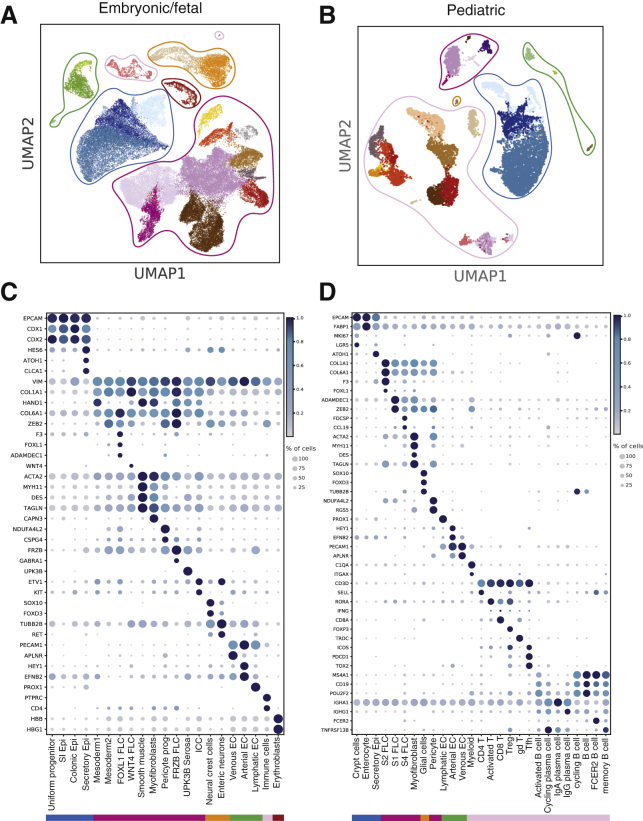
Cell-Type Groups and Their Marker Genes Identified in Fetal and Pediatric Datasets (A and C) UMAP plots of fetal and pediatric datasets (A and C, respectively) broadly grouped into seven groups: epithelial (blue), mesenchymal (dark pink), neural (orange), endothelial (green), immune (light pink), and erythroid lineage (brown). (B and D) Dot plots of relative expression and percentage of cells expressing marker genes in fetal (B) and pediatric (D) datasets. The color bars match the cell-type group colors. Epi, epithelium; FLC, fibroblasts; EC, endothelial cells; ICC, interstitial cells of Cajal.

Embryonic/fetal and pediatric datasets were processed individually to identify the cell types present in the samples. Clustering and cell-type-specific marker gene expression revealed seven major cell types in embryonic/fetal samples, including immune, erythroblast, endothelial, neural crest, smooth muscle (SMC), mesenchymal, and epithelial cell populations (Figures 2A–2D). Assessment of cellular subsets and their expression markers allowed us to further subdivide cell types, as outlined in Figures 1C and 1D. All major cell types were also identified in pediatric biopsy samples with the exception of enteric neurons, smooth muscle, and serosal cells (Figure 1D), whose exclusion was expected, given that the depth of forceps biopsy is restricted to mucosa.

Comparing the cellular composition across the three developmental stages (embryonic, fetal, and pediatric), we observed notable differences. For example, the mesenchymal cell compartment was greatly expanded in proportion as well as diversity in embryonic and fetal samples (Figures 1C and 1D). Conversely, pediatric samples were dominated by immune cells, including follicular/memory B cells, T cells, and plasma and myeloid cells. Embryonic and fetal samples contained a comparatively smaller proportion of immune cells, including macrophages, dendritic cells, monocytes, as well as T and B cells (data not shown).

Differentiated cell states were more frequently observed in the proximal than distal regions of the developing gut (Figure 1E). For example, we observed the highest proportion of secretory epithelial subset as well as cells of immune, erythrocyte, and endothelial lineages captured in the duo-jejunum, followed by expansion in the ileum, while the smallest proportion was observed in the colon at any given time point. Conversely, the undifferentiated mesodermal cells (mesoderm 1) were most abundant in the early colonic samples and decreased by 10 PCW, while the same subset was observed only in low abundance in the duo-jejunum samples even at the earliest time points (Figure 1E).

As shown in Figure 1F, the spatiotemporal distribution of individual cell clusters demonstrated a significant separation of embryonic/fetal epithelial cell clusters according to gut region and developmental time point (Figures 1F, 1G, and S1I). Temporal separation in epithelial cells of healthy pediatric samples was less pronounced (Figures S1C and S1E). These differences highlight major developmental changes in the intestinal epithelium during the captured time periods. Hence, we next aimed to further elucidate underlying mechanisms and pathways.

### Intestinal Epithelial Cell-Type Changes during Human Villus Formation

**Figure 3:**
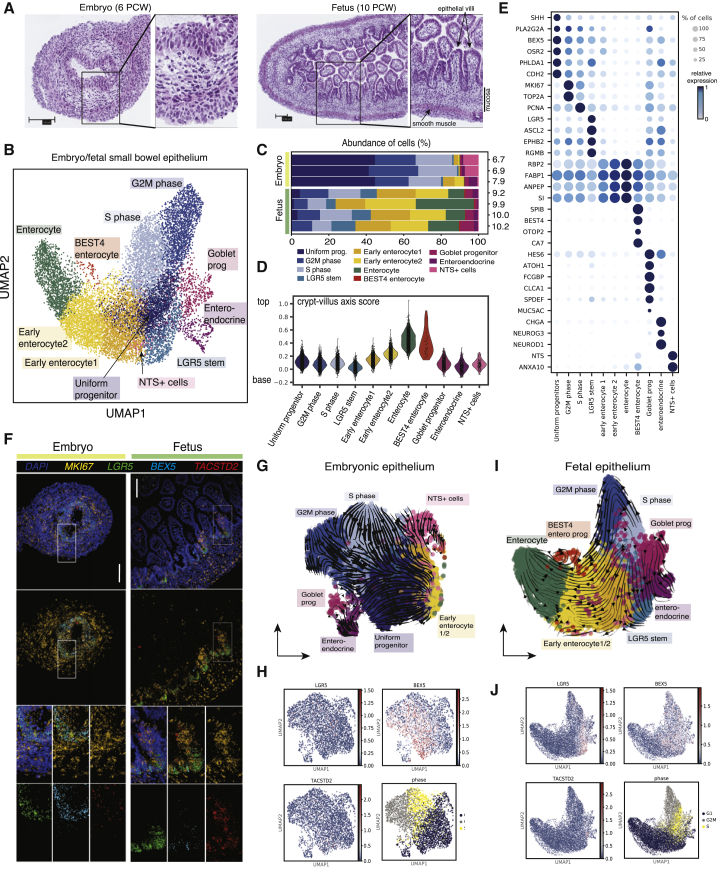
Epithelial Cell Composition during Villus Formation in Humans (A) Representative hematoxylin and eosin staining of embryonic and fetal ileum at 6 and 10 PCW (n = 3 donors). (B) Sub-clustered epithelial cells from duo-jejunum and ileum colored by cell type. (C) Changes in epithelial cell-type abundance (% of cells) at different developmental time points and in two small bowel regions. Colors match the cell-type annotation in (B). (D) Pseudo-spatial distribution of developing epithelial cells along the crypt-villus (base-top) axis. The axis score was derived by using the expression of selected crypt-villus axis markers as defined by Moor et al. (2018) and Parikh et al. (2019). (E) Dotplot with marker genes used to annotate fetal epithelial cell subtypes. (F) smFISH analysis of *MKI67*, *LGR5*, *BEX5*, and *TACSTD2* transcripts in embryonic (pseudostratified) and fetal (vilified) epithelium. (Embryonic: 6 PCW; fetal: 10 PCW.) Zoom-in boxes show channels with and without DAPI, as well as each channel independently. Scale bar: main panel, 100 μm; zoom panel, 50 μm. (G–J) (G) Embryonic and (I) fetal epithelium scVelo graphs with overlaid arrows. Expression of *LGR5*, *BEX5*, *TACSTD2*, and cell cycle phase overlaid on (H) embryonic and (J) fetal epithelial cells shown as feature plots. See also Figure S2.

Approximately midway through the first trimester, the human intestine is lined by a thick, pseudostratified epithelium that largely fills the intestinal lumen (Figure 3A). Only 3–4 weeks later a single-cell layer starts to appear and by 10 PCW a primitive villus structure can be observed (Figure 3A). In order to examine transcriptional changes that occur during this transition, we sub-clustered fetal small bowel (duo-jejunum and ileum) epithelial cells based on the expression of *EPCAM*. Following dimensionality reduction, we identified 11 epithelial cell clusters and their differentially expressed genes (Figures 3B and 2E). Among them were two clusters: a cluster with high expression of canonical adult-stem-cell genes, including leucine-rich repeat-containing G-protein coupled receptor 5 (*LGR5*), achaete-scute complex homolog 2 (*ASCL2*), ephrin type-B receptor 2 (*EPHB2*), and repulsive guidance molecule BMP co-receptor B (*RGMB*) (Figures 3B and 2E), and a cluster that was defined by high expression of sonic hedgehog (*SHH*), phospholipase A2 group IIA (*PLA2G2A*), brain expressed X-linked 5 (*BEX5*), and cadherin-2 (*CDH2*) (Figures 3C and 2E). The latter cluster, which we refer to as “uniform progenitors,” displayed relatively low or absent expression of *LGR5*, *ASCL2*, and *SMOC2* (Figures 3E and S2B). Conversely, the LGR5-high stem-cell cluster showed downregulation of genes expressed in the uniform progenitors, such as *BEX5* and *CDH2* (Figure S2C).

The number of uniform progenitors decreased at 10 PCW (Figure 3C), coinciding with villus emergence and the appearance of LGR5+ stem cells, immature and maturing enterocytes, and goblet and enteroendocrine cells (Figure 3C). Paneth cells were not evident and a population of *BEST4*/*OTOP2*+ enterocytes was present in the first trimester of development, confirming previous reports (Mallow et al., 1996; Parikh et al., 2019; Smillie et al., 2019). In addition, we used expression data to define the position of differentiating epithelial cell subsets along the crypt-villus (base-top) axis using scoring of gene sets previously reported in adults (Moor et al., 2018; Parikh et al., 2019) (STAR Methods). While immature and differentiated enterocytes as well as *BEST4*-enterocytes localized to the top of the axis, the cycling cells, *LGR5*+ stem cells, and uniform progenitors localized to the crypt bottom (Figure 3D).

In both chicken and mouse, the process of villus emergence affects epithelial proliferation (Walton et al., 2016a, 2016b). While epithelial cells located at the inter-villus region remain proliferative, those at the tip of the villus withdraw from the cell cycle. This is considered a critical event in the development of intestinal adult stem cells. We used *BEX5* as a marker of uniform progenitors alongside *LGR5* and *MKI67* to visualize epithelial changes during villus emergence. In addition, we included *TACSTD2* (*Trop2A* in mice), which has been shown to mark the murine fetal intestinal progenitors (Mustata et al., 2013). Our imaging confirmed that a similar proliferation restriction occurs in the developing human intestine, whereby embryonic epithelium is uniformly cycling, whereas at 10 PCW cycling cells become restricted to the inter-villus domains. We also noted that at the embryonic stages, uniform progenitors are *LGR5*-low and *BEX5*-high, whereas the cells restricted at the inter-villus domains upregulate *LGR5* expression and downregulate *BEX5* (Figure 3F). *TACSTD2* was expressed in both embryonic epithelium and fetal developing crypts, showing similarities to mouse models. Together, these results suggest that at the embryonic stages, the epithelium is composed of highly cycling, uniform progenitors that express low levels of LGR5.

In addition, we applied the scVelo and partition-based graph abstraction (PAGA) trajectory algorithms to epithelial cells of the small bowel (duo-jejunum and ileum) to better understand cell differentiation dynamics during the transition from embryonic (6–8 PCW) to early fetal (9–10 PCW) epithelium (Figures 3G–3J, S2D, S2E, and S2F–S2I). At the embryonic stages, rapidly cycling cells formed the start point of the trajectory and appeared to give rise to *BEX5-*high uniform progenitors. We also observed a proportion of differentiated cells, such as enteroendocrine and goblet cells, that may differentiate from cycling cells (Figures 3G–3J). Combined scVelo and PAGA trajectory analyses suggest that uniform progenitors may be capable of self-renewing as well as giving rise to differentiated cell lineages (Figures S2D, S2E, and S2F–S2I), as recently proposed in mice (Guiu et al., 2019). At the fetal stages, the differentiation dynamics change, suggesting the beginning of adult-like differentiation of LGR5+ stem cells into its progeny (Figures 3G–3J, S2E, and S2I). This finding indicates that the cycling epithelium undergoes transcriptional transition from uniform epithelium into *LGR5+* stem cells and therefore may act as both a primitive stem cell of the early gut and as a progenitor to *LGR5+* stem cells later in development.

In summary, *in silico* trajectory analysis revealed complex cell dynamics in the embryonic and fetal epithelium and further supports the conclusion that epithelium at embryonic stages represent a uniformly cycling epithelial progenitor cell.

### Cell-Cell Cross-Talk That Supports Villus Formation in Humans

**Figure 4:**
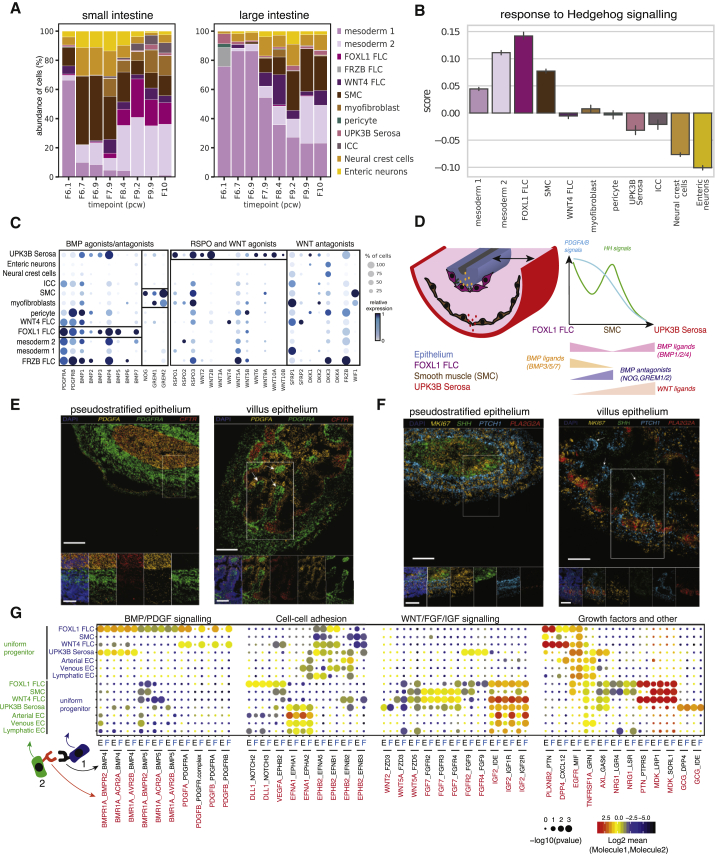
Cell-Cell Interactions that Support Transition from Embryonic to Fetal Epithelium in Humans (A) Abundance of mesenchymal and neuronal cell subsets (% of cells) in developing gut from small (left panel) and large intestines (right panel). (B) Average expression score of hedgehog (HH) pathway genes. (C) Dot plot with expression of *BMP* and *WNT* agonists/antagonist and *RSPO* genes in all mesenchymal cells. (D) Pseudo-positioning schematic of *PDGF* and *HH* receptor expression as well as *BMP* and *WNT* ligand expression in the cross-section of the developing small bowel upon villus formation. (E and F) (E) Visualization of *PDGF* ligand and receptor and (F) HH pathway genes in the embryonic small intestine using smFISH at two developmental time points (left panel: embryonic; right panel: fetal). Scale bar: main panel, 100 μm. *PLA2G2A* expression marks developing smooth muscle, UPK3B+ serosal cells, and uniform progenitor cells. (G) Dot plot of ligand-receptor interactions between uniform progenitor cells and mesenchymal/endothelial populations as predicted using CellPhoneDB analysis in embryonic (columns marked with E) and fetal (columns marked with F) samples. Point size indicates permutation p value and color indicates the scaled mean expression level of ligand and receptor. The interacting cell type and molecule pair relationship is explained in a schematic, where molecule 1 (black) in cell-type cluster 1 (blue) interacts with molecule 2 (red) in cell-type cluster 2 (yellow). FLC, fibroblasts; SMC, smooth muscle cells; EC, endothelial cells; ICC, interstitial cells of Cajal; PCW, post-conception weeks. See also Figure S3.

Remodeling of epithelium from pseudostratified to crypt-villus critically relies on the cross-talk with non-epithelial cell subsets. Next, we aimed to address mechanisms and signaling pathways implicated in human villus formation. First, we defined the changes in mesenchymal cell abundance across developmental time points in both small and large intestines in order to identify potential cell types that appear or become restricted during villus formation (Figure 4A). At sampled timepoints, we observed the disappearance of undifferentiated mesodermal subsets and the appearance of more differentiated mesenchymal subsets, suggesting remodeling of the mesenchymal compartment. Coinciding with epithelial remodeling, we detected the emergence of *FOXL1*+ fibroblasts in small intestinal regions. In contrast, developing SMCs were captured in both regions across embryonic and fetal time points (Figure 4A). Apart from high expression of PDGF receptor genes, *FOXL1+* fibroblasts were marked by expression of multiple BMP ligands (*BMP3/5/7*) as well as the adult colonic mucosal S2 fibroblast marker, *F3* (Kinchen et al., 2018). We further show that *FOXL1*+ cells transcriptionally best match adult S2 cells described in the colon (Kinchen et al., 2018) (Figures S3D and S3E). In addition, we visualized *PDGFRA-*high and *FOXL1/F3* expressing mesenchymal cell clustering around the thick, pseudostratified epithelium in 6 PCW human embryos (Figures 4E and S3B) and near-forming villi at 10 PCW (Figure 4E). This observation is reminiscent of mesenchymal clustering seen in E14.5 mouse embryos (Walton et al., 2016a, 2016b).

HH pathway activation is instrumental in regulating many aspects of intestinal development. For example, mesenchymal cluster size and subsequent villus emergence have recently been shown to be controlled by GLI2-driven activation of cell polarity pathway (Walton et al., 2012; Rao-Bhatia et al., 2020). In addition, GLI2 also regulates Wnt ligand expression in *FOXL1*+ telocytes, both in stomach and intestine (Kim et al., 2020). Using human scRNA-seq data, we further address which mesenchymal subsets show activation of HH signaling based on the co-expression of HH signaling pathway components (STAR Methods). The analysis identified mesodermal cells, *FOXL1*+ fibroblasts and SMCs as the main responders to HH signaling (Figure 4B). We further validated the expression of the Sonic hedgehog gene (*SHH*) by embryonic epithelium and *in situ* expression of its receptor, *PTCH1* (Figure 4F). We show that *PTCH1* expression forms ripples with high expression by cells located around the epithelium (at the site of clustering *FOXL1+* fibroblasts) and SMCs that were marked by *PLA2G2A* expression in scRNA-seq data (Figures 4C and S3A) as well as *in situ* (Figure S3C, white arrows).

While *FOXL1*+ fibroblasts highly expressed PDGF receptor and BMP ligand genes (Figure 4C), developing SMCs and myofibroblasts were marked by expression of BMP antagonists (*NOG*, *GREM1/2*) (Figure 4C). Similar opposing gradients were reported in the microenvironment of the adult mouse gut (McCarthy et al., 2020). We also observed expression of multiple WNT and RSPO ligand genes primarily in mesothelial serosa cells (Figure 4C), and further visualized the expression of *WNT2B* transcripts in these cells *in situ* (Figure S3B). We summarize these findings in the schematic (Figure 3D).

Using CellPhoneDB (Vento-Tormo et al., 2018; Efremova et al., 2020), we found that the mesenchymal populations displaying the highest number of cell-type-specific interactions with epithelial cell types were *FOXL1+* and *WNT4+* fibroblasts in both fetal and embryonic time points as well as mesothelial serosal cells in embryonic timepoints (Figures S3F and S3G). The most specific interactions between *FOXL1*+ fibroblasts and the uniform progenitors were via BMP, PDGF, Notch, Wnt, and FGF signaling pathways (Figure 4G). Other ligands secreted by *FOXL1+* fibroblast included *NRG1*, *CXCL12* and *VEGFA*. We also identified growth factors, such as *IGF2*, *PTN*, and *MDK*, as secreted by this mesenchymal subset and received by uniform progenitors (Figure 4G).

Together, these data demonstrate interactions relevant to the human villus formation and point toward signaling pathways implicated in early human gut development.

### Fetal Organoids Show *In Vitro* Maturation Recapitulating *In Vivo* Epithelial Transition

The ability of intestinal epithelial stem cells to give rise to all cell subsets has led to the development of intestinal organoid culture models (Sato et al., 2011a, 2011b; Tsai et al., 2017). Such organoids can be generated from the human fetal gut, providing the opportunity to investigate epithelial cell-intrinsic and -extrinsic developmental mechanisms (Fordham et al., 2013; Kraiczy et al., 2019). Here, we applied scRNA-seq to developing human gut organoids (STAR Methods, Table S1) and performed *in silico* analyses by classifying epithelial cells using transcriptional profiles derived from primary tissue.

**Figure 5:**
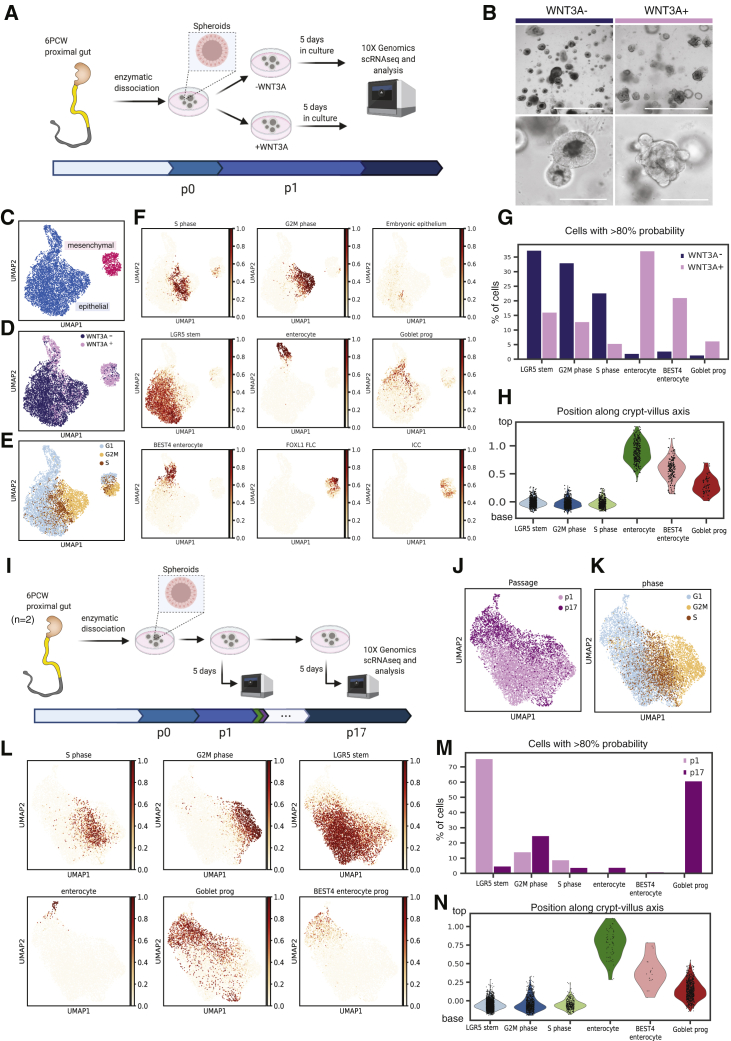
Fetal Intestinal Organoids Mature in Culture (A) Schematic representation of WNT3A organoid culture experiment. (B) Brightfield images of fetal organoids grown without (WNT3A−) or with (WNT3A+) conditional medium at passage 2, day 5 post-passage. Scale bar: top panels, 1,000 μm, top panels, 200 μm. UMAP plots of single cells from fetal organoids grown with or without WNT3A. (C–E) Cells are colored by either (C) cell type, (D) condition, or (E) cell cycle phase. (F) Cell-type prediction in WNT3A± organoid culture using logistic regression classifiers trained on all primary small intestinal fetal cells. UMAP plots show overlaid predicted probability for selected cell types. Abundance of cell types (% of cells) in organoids, as confidently predicted (over 80% probability of a single cell type) by the logistic regression classifier. (H) Pseudo-spatial distribution of organoid epithelial cells along the crypt-villus (base-top) axis. (I) Schematic representation of experimental design. The experiment was performed using two independent biological samples (replicate 1 from 5.4 PCW and replicate 2 from 6.4 PCW). (J and K) On day 5 of passage 1 (p1) organoids were split and one fraction was kept in the culture while the other was dissociated and processed using 3′ V2 10× protocol. On day 5 of passage 17 (p17) the organoids were dissociated and processed using the 10× platform again. UMAP plots with single cells colored by either (J) passage or (K) cell cycle phase. (L) Prediction of cell types of p1 and p17 organoids using logistic regression trained on primary fetal cells. UMAP plots represent visualization with overlaid predicted probability for selected cell types as in (F). (M) Predicted cell-type abundance or PCW age of fetal organoids grown for 1 or 17 passages as predicted using logistic classifier. N) Pseudo-spatial distribution of organoid epithelial cells along the crypt-villus (base-top) axis. FLC, fibroblasts; SMC, smooth muscle cells; EC, endothelial cells; ICC, interstitial cells of Cajal; PCW, post-conception weeks; prog, progenitor. Organoids were derived from fetal tissues BRC2038- 6.4 PCW, BRC2039 −5.4 PCW, BRC2206- 6.5 PCW. See also Figure S4.

In the adult small bowel mucosa, Paneth cells have been found to express the Wnt-agonist WNT3A, thereby providing a critical signal to the stem-cell niche (Sato et al., 2011a, 2011b). In addition to the absence of Paneth cells in the developing gut, we were also unable to identify any *WNT3A*-expressing mesenchymal cells in our scRNA-seq datasets (Figure 4C). Culture of intestinal epithelial organoids (IEOs) in the presence of recombinant WNT2B protein, expressed by the embryonic/fetal mesenchymal subsets (Figure 4C), did not lead to morphological difference or transcriptional activation of the Wnt signaling pathway (Figures S4D–S4H). However, given that WNT3A forms a key ingredient of previously reported human adult and fetal mucosa-derived intestinal epithelial organoid cultures (Sato et al., 2011a, 2011b; Fordham et al., 2013; Kraiczy et al., 2019) we aimed at assessing its requirement and impact on fetal gut organoids. IEO cultures were generated from the proximal small bowel and cultured in the presence or absence of WNT3A-conditioned medium (Figure 5A). Inclusion of WNT3A led to the presence of budding, crypt structures, while organoids lacking WNT3A appeared more spheroid-like (Figure 5B). Interestingly, single-cell transcriptional profiling of these cultures at an early passage (i.e., passage 2—approximately 2–3 weeks in culture) revealed the presence of intestinal epithelial cells as well as a small fraction of mesenchymal cells that resembled *FOXL1*-fibroblasts (Figures 5F, S4A, and S4B). Organoids were viable for several weeks even if cultured in the absence of WNT3A and showed evidence of active cell cycling (Figure 5E). However, WNT3A was required for long-term culture as WNT3A− organoids showed reduced viability and could not be cultured beyond 6 weeks. Importantly, observed phenotypic differences were matched by dramatic transcriptional changes leading to a clear separation of cells according to culture conditions (Figure 5D). Removing or reducing WNT3A in adult mucosa-derived IEOs has been shown to induce differentiation of epithelial cells and a reduced expression of LGR5 (Kraiczy et al., 2019). In contrast, when assessing epithelial cell identity and composition of fetal organoids using a logistic regression model trained on the primary fetal scRNA-seq profiles, organoids cultured in the presence of WNT3A were found to contain a greater proportion of differentiated cell types, including enterocytes and enteroendocrine cells compared with those cultured in its absence (Figures 5F and 5G).

Previous work suggests that human fetal gut organoids undergo a degree of *in vitro* maturation in culture (Tsai et al., 2017; Kraiczy et al., 2019). In order to examine this further, we generated organoids from embryonic gut samples aged 6 PCW and kept them in complete culture medium (containing WNT3A) over 5 months (Figure 5I). Single-cell profiling was applied to cultures once they were first established (after one week, passage 1) and following 5 months in culture (17 passages). Uniform manifold approximation and projection (UMAP) clustering revealed separation according to duration in culture, suggesting that significant transcriptional changes occur over time (Figure 5J). Major differences were also observed with regard to the predicted cell composition, such that older cultures contained a higher proportion of differentiated cell types, including enterocytes and goblet cell progenitors (Figures 5L, 5M, and S4C). Similar to the primary fetal scRNA-seq, organoid-derived enterocytes and BEST4-enterocytes were predicted to localize to the top of the villus axis, while the cycling cells—LGR5-stem cells, to the bottom of this axis (Figures 5H and 5N)—providing further evidence of epithelial cell maturity.

In summary, our findings reveal effects of Wnt signaling and specifically WNT3A on human fetal epithelial organoid cell diversity and maturity.

### Parallels between Fetal and Inflamed Epithelium in Crohn’s Disease Patients

Alterations in the composition, function, and cell dynamics of the intestinal epithelium are thought to play a critical role in the pathogenesis of CD. Moreover, a link has been proposed between early fetal development and regenerating epithelium in mice by demonstrating partial reprogramming of the regenerating colonic epithelium (Yui et al., 2018; Wang et al., 2019). In order to investigate these observations in humans, we performed scRNA-seq terminal ileum biopsies obtained from children newly diagnosed with CD (n = 7).

Compared with non-IBD samples, CD patients showed increased vascularization marked by expansion of arterial and venous endothelial cells as well as increased numbers of fibroblasts resembling S4 stromal cells (Figure S5C) first described in adult ulcerative colitis patients (Kinchen et al., 2018). We directly compared their transcriptional profiles using marker genes and a logistic regression model that showed high transcriptional similarity between colonic and ileal stromal populations (Figures S6A–S6F). In the immune compartment, we observed expansion of myeloid cells, CD4 T cells, and IgG plasma cells (Figure S5D), features recently described in ileal CD patients (Martin et al., 2019).

**Figure 6:**
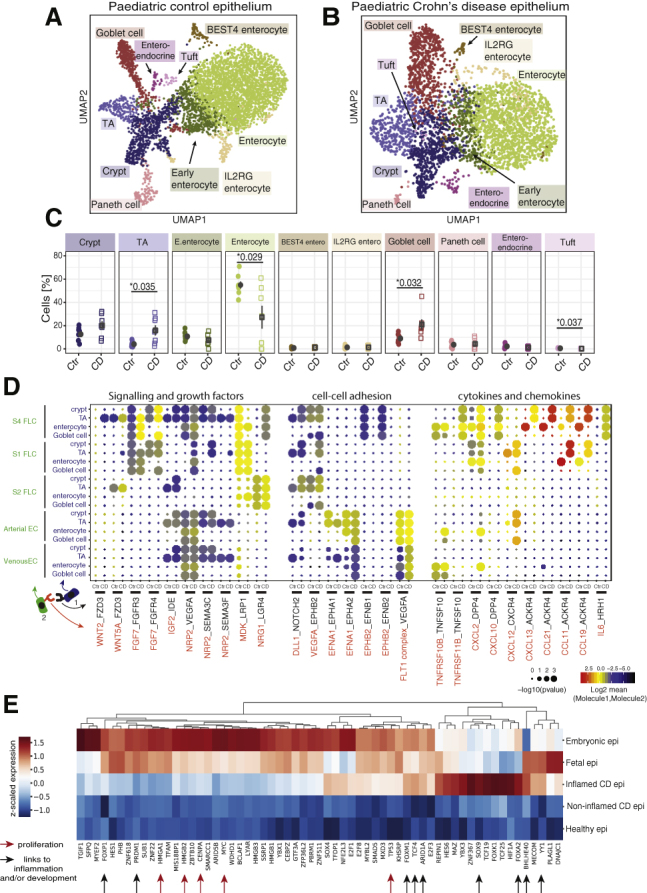
Epithelial Cell Dynamics in Crohn’s Disease Patients Show Transcriptional Similarities to Developing Epithelium (A and B) (A) and (B) UMAP plots of epithelial cell subtypes in healthy children (n = 8) and patients with CD (n = 7), respectively. (C) Epithelial cell-type changes in pediatric health and CD patients. TA, enterocytes, goblet cells, and tuft cell proportions were changed significantly between control and CD patients (p values indicated, t test). (D) Dot plot with ligand-receptor interactions between stromal (S1-S4 FLC) and endothelial cells (Arterial/venous EC) and selected epithelial cell types. Point size indicates permutation p value (CellPhoneDB). Color indicates the scaled mean expression level of ligand and receptor. FLC, fibroblasts; EC, endothelial cell. (E) Heatmap showing the relative mean expression of transcription factors, which were identified to be differentially expressed in CD epithelium, across epithelium from five groups. “Non-inflamed CD” was a group of patients with minimal epithelial composition changes as in (Figure S5F, arrows). Arrows point to genes discussed in text that either have previously been linked to proliferation (red arrows) or inflammation and/or development (black arrows). Epi, epithelium; CD, Crohn’s disease. See also Figures S5 and S6.

Comparing epithelial cell composition between CD and age-matched control samples, we observed significant differences, including an increase in transit amplifying (TA), goblet, and tuft cells, while the proportion of fully differentiated enterocytes was significantly reduced in CD epithelium (Figures 6A–6C and S5F). We then interrogated the cross-talk between stromal cells and the affected intestinal epithelial subtypes in the context of CD. We identified a number of cell-cell interactions that were specific between CD cell-type pairs (Figure 6D). For example, S4 fibroblasts were found to uniquely signal to CD TA cells via a *WNT2* ligand that were received by epithelial subtypes via *FZD3* receptor (Figure 6D). In addition, we observed chemokines and cytokines, such as *CXCL2*, *CXCL10*, *CXCL13*, *CCL11*, *and IL6* that were expressed by the S4 fibroblast population and received by intestinal epithelial cells. Interestingly, among cytokine interactions we found *TNFSF10-TNFRSF10B* signals that promote tumor necrosis factor (TNF)-related apoptosis and elimination of intestinal epithelial cells (Begue et al., 2006; Wu et al., 2019). Compared to healthy patients, specificity of *TNFSF10-TNFRSF10B* signaling in CD patients was reduced between goblet cells and S4 fibroblasts, while it remained similar in enterocytes. These changes may contribute to the selective loss of enterocytes and increase in goblet cell abundance seen in CD. Together, these findings highlight the complex cross-talk between the epithelium and surrounding stromal cells, which is likely to contribute toward chronic mucosal inflammation observed in childhood-onset CD.

Finally, growth factor interactions that we observed during development, such as interactions via *WNT5A*, *FGF7*, *IGF2*, *MDK*, and *NRG1* ligands, were also changed between controls and CD patients. To further understand the regulation of cell-cell interactions, we aimed to identify the transcription factors that were shared between CD and developing epithelium (STAR Methods). As shown in Figure 6E, we identified a number of such genes, some of which have been linked to IBD pathogenesis. Examples include the B lymphocyte-induced maturation protein-1 (Blimp1; encoded by the gene *PRDM1*) (Harper et al., 2011; Muncan et al., 2011; Ellinghaus et al., 2013); Forkhead Box transcription factors, *FOXP1* and *FOXM1*, linked to CD (de Lange et al., 2017; Bo et al., 2018); tumor suppressor *ARID1A*, recently reported to be under positive selection for somatic mutations in IBD colon (Olafsson et al., 2020); cell proliferation genes, such as *TP53* and *MYC*, associated with inflammation induced colorectal cancers (Horvath et al., 2015; Du et al., 2017; Lu et al., 2017); and *HMGA1/HMGB2* genes involved in the stem-cell expansion and associated with IBD (Vitali et al., 2011; Takaishi et al., 2012; Bush et al., 2013; Xian et al., 2017). Furthermore, our analyses identified several transcription factors implicated in stem cell and embryonic developmental. For example, the Wnt signaling transcription factor, TCF4 (Barker et al., 1999; Wehkamp et al., 2007), as well as *FOXA2*, and *SOX9*, expression of which is associated with development.

Taken together, our results confirm previous reports of altered intestinal epithelial cell dynamics in regenerating CD epithelium and identify several disease-associated cell-cell interactions in childhood-onset CD. Importantly, we provide supportive evidence for the partial re-activation of developmental transcriptional pathways in CD epithelium.

## Discussion

Previous studies have reported the presence of proliferative, immature progenitor epithelial cells in the human fetal intestine at around 10 PCW (Fordham et al., 2013; Guiu et al., 2019). We demonstrate that this cell population forms the vast majority of pseudostratified intestinal epithelium in the human embryo (6–8 PCW) and expresses *CDH2*, *BEX5*, *SSH*, and *PLA2G2A*, all of which have been previously linked to the stem-cell potential. For example, *CDH2* was linked to regulation of cell fate decision in the mesodermal lineage (Alimperti and Andreadis, 2015), and *SHH* to the initiation of villus formation in the developing mouse intestine (Shyer et al., 2015), while BEX-family genes were found to be expressed in tissue stem/progenitor cells (Ito et al., 2014). In addition, we demonstrate *LGR5* expression in the embryonic epithelium, albeit at lower levels than in fetal tissue. At the embryonic stages, all epithelium is uniformly cycling, while at the fetal stages, cycling cells become restricted to the bottom of the inter-villus domains, and upregulate *LGR5* expression. While analogous processes of early epithelial development were previously reported in chicken and mouse (Shyer et al., 2015), scRNA-seq data provide insights into the diversity and maturity of epithelial cell types found at fetal stages. Nevertheless, lineage tracing experiments in organoid cultures are necessary to provide evidence on the source of LGR5+ stem cells in humans.

The presence of secretory cells, including enteroendocrine and goblet cells, in multiple embryonic samples prompted us to hypothesize that the more abundant, cycling, uniform progenitors, as opposed to a few captured LGR5 stem cells, may be the source of secretory cells. These observations are in keeping with recent lineage tracing experiments by Guiu et al. (2019) that suggest the presence of alternative sources for differentiated cell subsets during development, given that fetal LGR5 cells alone are unable to sustain intestinal growth. *In silico* trajectory analysis imply that the uniform embryonic progenitors and cycling cells may be the source of differentiated cell subsets captured in embryonic stages; however, future studies are needed to address the source of differentiated cell subsets in the fetal gut.

Intestinal villus formation is one of the key developmental milestones of the first trimester. While common pathways have been proposed to mediate villus formation in chicken and mouse (*Bmp*, *Hh*, *Pdgf*), the mechanisms differ between species. In the chicken model, force generated by smooth muscle progressively deforms the epithelium to generate mucosal folds. In turn, the mesenchymal clusters form at the villus tips and restrict epithelial proliferation via Bmp signals (Shyer et al., 2015). A different mechanism was proposed in mice, where villus formation is driven by self-organizing mesenchymal cell clusters (Karlsson et al., 2000; Walton et al., 2012, 2016a, 2016b) and is uncoupled from the development of smooth muscle layers. Recent reports implicate the mesenchymal Fat4/PCP pathway in organization of mesenchymal clusters and demarcation of the emerging villus (Rao-Bhatia et al., 2020).

Using scRNA-seq data, we show that the appearance of FOXL1 fibroblasts was coincidental with epithelial changes in human intestinal epithelium. Foxl1 in mice marks a population of subepithelial telocytes that are essential for the intestinal stem-cell niche (Shoshkes-Carmel et al., 2018; Kaestner, 2019; McCarthy et al., 2020). In adult mice, telocytes support epithelial zonation along the villus and were recently shown to express Lgr5, Bmp, and Wnt ligands at the villus tip (Bahar Halpern et al., 2020). Our scRNA-seq and imaging data suggest that *FOXL1+* fibroblasts start developing at the embryonic stage in the absence of any visible villi. *FOXL1+* fibroblasts were also the main responders to HH signaling and expressed BMP and PDGFR genes during development, suggestive of their role as clustering mesenchymal cells critical for villus formation. Using *in silico* ligand-receptor analyses, we further identified multiple ligands-encoding genes, including *WNT5A*, *WNT2*, *NRG1*, and *IGF2*, with potential to modulate early villus formation in humans. Finally, we show transcriptional similarities between *FOXL1+* fibroblasts and *S2+* fibroblasts found in the human adult gut, suggesting that two cell states may represent the same lineage of mesenchymal cells equivalent to murine telocytes.

The mesenchymal cell clustering and subsequent villus emergence were proposed to follow a proximal-to-distal wave (Spence et al., 2011; Walton et al., 2012). Fordham et al. (2013) challenged this view by culturing fetal epithelial cells from proximal, middle, and distal regions of the mouse gut and observing the opposite trend of differentiation in the organoid system. While in this work restricted cell lineages were traced (Pdgfra+ mesenchymal cell clusters or differentiating epithelial cells), scRNA-seq allows for the investigation of multiple cell lineages at the same time. Our dataset provides evidence that multiple cell lineages differentiate and, in case of immune cells, home to the proximal intestinal regions first. We also observed differences in vascularization between three regions, where endothelial cells differentiated in the proximal-to-distal wave during human intestinal development.

IEOs have been generated from human fetal gut and shown to undergo a degree of *in vitro* development highlighting their use as powerful experimental tools (Fordham et al., 2013; Guiu et al., 2019; Kraiczy et al., 2019). Here, we combined generation of fetal organoids with single-cell profiling to interrogate fetal culture composition. Our findings indicate that, while not required for their establishment and short-term culture, WNT3A is essential for long-term propagation of fetal organoids and was found to be associated with a higher proportion of differentiated cell subsets. This parallels studies in mice, where embryonic progenitors were able to proliferate independent of Wnt prior to villus formation but not after (Chin et al., 2016). These findings also point to differences between adult and fetal gut epithelium as the generation of adult mucosa-derived intestinal organoids critically relies on the presence of WNT3A, while its withdrawal leads to increased differentiation into epithelial cell subsets and reduced expression of *LGR5* (Fordham et al., 2013; Kraiczy et al., 2019). Furthermore, organoids kept in culture for several months were found to contain an increased proportion of differentiated cell subsets as well as an increased number of LGR5+ cells. This suggests that current intestinal culture conditions select for highly proliferating cells. Finally, our *in vitro* studies further illustrate the utility of single-cell transcriptomics as a critical reference for validating and interpreting fetal organoid culture work.

A developmental origin of disease pathogenesis has been proposed for many complex, multifactorial conditions. IBD, and particularly CD, are thought to be caused by a complex interplay between the environment and genetic predisposition leading to an irreversibly altered immune response. Recent studies have reported expansion of a colonic mesenchymal subset in adult ulcerative colitis and associated this with resistance to anti-TNF treatment (Kinchen et al., 2018; Smillie et al., 2019). We observed expansion of a similar mesenchymal population in childhood CD, suggesting similarities between adult- and pediatric-onset IBD. In addition, comparison between CD and healthy epithelium suggests that CD epithelium is rapidly cycling and poised for goblet cell differentiation, consistent with previous reports (Gersemann et al., 2009). Furthermore, we describe ligand and receptor pairs that uniquely signal between affected epithelial subsets and expanded stromal populations, including WNT2 ligands received by TA cells. This provides a possible mechanism to sustain intestinal regeneration in disease.

Previous studies in mice have linked epithelial cell properties in the inflamed gut to the physiological status observed in early fetal development (Yui et al., 2018; Guiu et al., 2019; Wang et al., 2019). Here, we provide evidence in humans that regenerating CD epithelium shares transcription factor programs otherwise present only in fetal epithelium. Identified transcription factors including *TP53*, *MYC*, *HMGA1*, and *HMGA2* point to increased epithelial proliferation, which we also observed as an increase in TA cell abundance. Other genes have been reported in development of epithelium or inflammatory cells in other organs. Among them is the zinc-finger transcription factor, Yin and Yang (*YY1*), which has been shown to play a critical role in lung epithelial cell development and TGF-beta-induced lung fibrosis (Boucherat et al., 2015; Zhang et al., 2019). Another example is the expression of basic helix–loop–helix 40 (*BHLH40*), expression of which has been observed in a wide range of cells and tissues, including T cells, macrophages, dendritic cells, and the gastric epithelium (Lin et al., 2014; Teng et al., 2020). *BHLH40* was found to control cytokine production by T cells, thereby playing a critical role in the development of autoimmune neuroinflammation (Lin et al., 2014; Yu et al., 2018). Finally, our analyses confirm previous reports of Forkhead BoxM1 (*FOXM1*, also HFH-11) transcription factor being expressed in embryonic epithelial cell with its expression becoming reactivated in adult cell types by proliferative signals or oxidative stress (Ye et al., 1997).

In summary, we provide a detailed single-cell map of the human gut during embryonic, fetal, and pediatric health as well as during inflammatory disease and dissect transcriptional changes in epithelial cell dynamics during intestinal life.

## STAR★Methods

### Key Resources Table

**Table:** 

REAGENT/ RESOURCE	SOURCE	IDENTIFIER
**RNAscope probes**		

BEX5	ACD, Bio-Techne	581118
CFTR	ACD, Bio-Techne	603298-C2
F3	ACD, Bio-Techne	407618-C2
FOXL1	ACD, Bio-Techne	558088-C3
LGR5	ACD, Bio-Techne	311028-C2
MKI67	ACD, Bio-Techne	591778-C3
PDGFA	ACD, Bio-Techne	406728-C4
PDGFRA	ACD, Bio-Techne	604488
PLA2G2A	ACD, Bio-Techne	581108-C4
PTCH1	ACD, Bio-Techne	405788
SHH	ACD, Bio-Techne	600958-C2
TACSTD2	ACD, Bio-Techne	405478-C4
UPK3B	ACD, Bio-Techne	581098-C4
WNT2B	ACD, Bio-Techne	453368

**RNAscope reagents**		

RNAscope Multiplex Fluorescent Reagent Kit	ACD, Bio-techne	322800
RNAscope 4-plex Ancillary Kit for Multiplex Fluorescent Reagent Kit	ACD, Bio-techne	322830
Opal 520	Akoya Biosciences	FP1487001KT
Opal 570	Akoya Biosciences	FP1488001KT
Opal 650	Akoya Biosciences	FP1496001KT
TSA-biotin	Akoya Biosciences	NEL749A001KT
Streptavidin-conjugated Atto 425	Sigma Aldrich	09260-1MG-F

**Biological Samples**		

Human fetal intestinal tissue	University of Cambridge	Acquired directly from ethically approved internal study (REC-96/085)

**Chemicals, Peptides, and Recombinant Proteins**		

Chromium Single Cell 3’ Library & Gel Bead Kit v2	10x Genomics	Catalogue # PN-120237
WNT3A	Conditioned cell line (internal)	N/A
R-Spondin-1	Conditioned cell line (internal)	N/A
EGF	Invitrogen	PHG0313
A8301	Tocris	2939
Y27632	Sellech Chem	S1049
Noggin	N/A	N/A
B27	Thermo Fisher Scientific	17504044
HEPES buffer	Gibco (Life Technologies)	15630080
Glutamax	Gibco (Life Technologies)	35050061
DMEM-F12	Gibco (Life Technologies)	11320033
Liberase DH	Sigma Aldrich	5401054001
WNT2B	Abcam	ab132538
Hyaluronidase	Merck	HX0514

**Deposited Data**		

Fetal, pediatric and organoid single cell RNAseq data	This study	https://www.ebi.ac.uk/arrayexpress/experiments/E-MTAB-8901/
Single cell RNAseq data	GEO: GSE134809	Martin et al., 2019
Single cell RNAseq data	GEO: GSE114374	Kinchen et al., 2018

**Software and Algorithms**		

Cellranger 10x Genomics, version 2.1.1 (reference transcriptome GRCh38-1.2.0)	https://support.10xgenomics.com/single-cell-gene-expression/software/pipelines/latest/installation	Zheng et al., 2017
Scanpy package v1.4	https://icb-scanpy.readthedocs-hosted.com/en/stable/	Wolf et al., 2018
Scrublet	https://github.com/AllonKleinLab/scrublet	Wolock et al., 2019
human_cycle_markers.rds, scran package	https://github.com/MarioniLab/scran/tree/master/inst/exdata	Scialdone et al., 2015
BBKNN	https://bbknn.readthedocs.io/en/latest/	Polański et al., 2020
UMAP	https://scanpy.readthedocs.io/en/stable/api/scanpy.tl.umap.html	Becht et al., 2018
Leiden	https://scanpy.readthedocs.io/en/stable/api/scanpy.tl.leiden.html	Traag et al., 2019
scVelo 1.24 package	https://github.com/theislab/scvelo	Bergen et al., 2020
PAGA	https://github.com/theislab/paga	Wolf et al., 2019
CellPhoneDB v2.0	https://www.cellphonedb.org/	Efremova et al., 2020
Sklearn implementation linear_model.LogisticRegression	https://scikit-learn.org/stable/modules/generated/sklearn.linear_model.LogisticRegression.html	Pedregosa et al., 2011
fdrtool package (R version 3.5.0)	https://cran.r-project.org/web/packages/fdrtool/fdrtool.pdf	Strimmer, 2008
Pandas, Version 0.25.2	https://pandas.pydata.org/	N/A
NumPy, Version 1.16.2	https://pypi.org/project/numpy/1.16.2/	N/A
Anndata	https://pypi.org/project/anndata/	N/A
Scipy	https://www.scipy.org/	N/A

### Resource Availability

#### Lead Contact

Further information and requests for resources and reagents should be directed to and will be fulfilled by the Lead Contact; Matthias Zilbauer (mz304@medschl.cam.ac.uk).

#### Materials Availability

Human cell lines developed in this study can only be distributed following MTA arrangement.

#### Data and Code Availability

The accession number for the raw sequencing data reported in this paper is E-MTAB-8901. Processed single-cell RNA sequencing objects will be available for online visualisation and download at gutcellatlas.org. The code generated during this study will be available at Github https://github.com/Raselel/DevCell_GutAtlas/.

### Experimental Model and Subject Details

#### Fetal and Paediatric Tissue Sampling

First trimester human fetal tissue was collected from patients undergoing elective termination of pregnancy. Patients gave informed consent as part of the ethically approved research study (REC-96/085). Fetal age (post conception weeks, PCW) was estimated using the independent measurement of the crown rump length (CRL), using the formula PCW (days) = 0.9022 × CRL (mm) + 27.372. Fetal sample ages in post-conception weeks were as follows: BRC2029 - 6.1, BRC2026 - 8.4, BRC2043 - 10.2, BRC2046 - 6.7, BRC2049- 6.9, BRC2121 - 9.2, BRC2119- 7.9, BRC2133 - 9.9, BRC2134- 10. Organoids were derived from fetal tissues BRC2038- 6.4, BRC2039 - 5.4, BRC2206- 6.5.

Human intestinal mucosal biopsies were obtained from patients undergoing colonoscopy at Addenbrooke's Hospital, Cambridge, UK. All patients gave informed consent for extra biopsy samples to be taken for research use when undergoing elective colonoscopy (REC 17/EE/0265). Patients were then included if, after macroscopic visualization and histological analysis, they were diagnosed as either having CD, or being without an inflammatory diagnosis (control). Patient ages were as follows: Control group (T036- 4 years, T110- 4 years, T161- 4 years, T057- 6 years, T182- 9 years, T44- 10 years, T024- 12 years, T160- 10 years); CD group (T197- 9 years, T176- 11 years, T019- 12 years, T202- 12 years, T017-13 years, T189-13 years, T203-14 years).

### Method Details

#### Fetal and Paediatric Tissue Dissociation

Fetal intestinal samples were dissected into duodenum-jejunum (further referred to as the duo-jejunum), ileum and colon using anatomical landmarks, and processed to single-cell suspension in parallel. Both fetal samples and paediatric samples were processed using the same protocol. Briefly, paediatric biopsies or fetal tissue sections were immediately rinsed twice with Hank’s Balanced Salt Solution (HBSS) medium (Sigma-Aldrich) and digested in HBSS medium containing 1.07 Wünsch units/ml of Liberase DH (Roche) and 600 IU of Hyaluronidase (Calbiochem) on a shaking platform (750 rpm) at 37°C for up to 30 min. The tissue was gently homogenised using a P1000 pipette every 15 mins. A single-cell suspension was then passed through a 40 μm cell strainer to remove undigested tissue. Cells were spun down at 400 g at 4°C for 5 min and the pelleted cells were washed in DMEM/F12 three times using centrifugation.

Fetal cells were either loaded for scRNAseq directly following sample processing or subjected to EPCAM selection to enrich epithelial cells. For enrichment, single cells were suspended in MACS modified solution (PBS with 0.5% BSA, 2 mM EDTA and 100 IU/mL DNaseII) and stained with EPCAM (CD326) magnetic microbeads (Milteny biotec) according to the manufacturer’s protocol. Enrichment was performed using an autoMACS Pro Separator. Either only EPCAM positive (PCW 6.7, 6.9, 10.2, 9.3) or both EPCAM positive and negative (PCW 9.9, 10.1, 10) fractions were processed using the 10x Genomics single-cell transcriptomics system. All paediatric single-cell suspensions were subjected to the MACS enrichment using the same protocol as described for fetal samples. Both fetal and paediatric single-cell suspensions were carried forward into single-cell sequencing only if the viability was >60% (Table S1).

#### Intestinal Organoid Culture

Fetal organoids were cultured in Matrigel® (Corning) using media described in (Fordham et al., 2013) and also provided in Table S2. During organoid culture, the media was replaced every 4872hours. Organoids were passaged using mechanical disruption with a P1000 pipette and re-seeded in fresh growth-factor reduced Matrigel® (Corning). When comparing culture media, multiple wells were seeded from a single dissociated sample, and wells assigned to either of the media. Organoid lines grown in WNT2B were grown in identical conditions, but with WNT3A replaced by recombinant human WNT2B at 100ng/μl (Abcam).

Organoids were derived from fetal ileum from embryos aged 5.5 and 6.4 PCW (BRC2038, BRC2039) and were maintained *in vitro* for 17 passages. Organoids were dissociated for single cell RNA-sequencing at passage 1 (1 week) or passage 17 (~4 months) in culture and profiled using 10x Genomics single-cell transcriptomics. For the WNT2B/WNT3A comparison experiment, cells from fetal ileum from an embryo at 6.4 PCW (BRC2206) were used to generate organoid lines in either WNT2B and WNT3A, with dissociation for single cell analysis performed five days after the first passage. Processing for single-cell sequencing analysis was performed by removing the organoids from matrigel using incubation with Cell Recovery Solution at 4^°^C for 20 minutes, pelleting the cells, and re-suspending in TrypLE enzyme solution (Thermo Fisher) for incubation at 37^°^C for 10 mins. Cells were pelleted again and re-suspended in DMEM/F12. Brightfield images of organoids were taken using an EVOS Cell Imaging Systems microscope (Thermo Fisher).

#### Tissue Freezing, Sectioning and RNAscope

Tissue was prepared, stained, and imaged largely as described previously (Bayraktar et al., 2020). In short, fresh tissue samples were either embedded in OCT and frozen at -80°C on an isopentane-dry ice slurry, or fixed in 10% neutral-buffered formalin at 4°C for ~24 hours, and then embedded and frozen as above. Cryosections were cut at a thickness of 10-16 μm using a Leica CM3050 S cryostat and placed onto SuperFrost®Plus™ slides (VWR). Prior to staining, tissue sections were post-fixed in 4% paraformaldehyde in PBS for 15 minutes at 4°C, then dehydrated through a series of 50%, 70%, 100%, and 100% ethanol, for 5 minutes each.

Tissue sections were then processed using a Leica BOND RX to automate staining with the RNAscope® Multiplex Fluorescent Reagent Kit v2 Assay and RNAscope® 4-plex Ancillary Kit for Multiplex Fluorescent Reagent Kit v2 (Advanced Cell Diagnostics, Bio-Techne), according to the manufacturers’ instructions. Automated processing of non-fixed sections included pre-treatment with Protease IV for 30 minutes, but no heat treatment; fixed frozen sections were subjected to heat-induced epitope retrieval at 95°C in buffer ER2, and digestion with Protease III for 15 minutes. Tyramide signal amplification with Opal 520, Opal 570, and Opal 650 (Akoya Biosciences) was used to develop three probe channels. The fourth was developed using TSA-biotin (TSA Plus Biotin Kit, Perkin Elmer) and streptavidin-conjugated Atto 425 (Sigma Aldrich).

Stained sections were imaged with a Perkin Elmer Opera® Phenix™ High-Content Screening System, in confocal mode with 1 μm z-step size, using 20× (NA 0.16, 0.299 μm/pixel) and 40× (NA 1.1, 0.149 μm/pixel) water-immersion objectives.

#### Single-cell RNA-sequencing

Single-cell suspension for each primary intestinal or organoid sample was loaded onto a separate channel of a Chromium 10x Genomics single cell 3’v2 library chip as per manufacturer's protocol (10x Genomics; PN-120233), aiming for a cell capture recovery of 3000-5000 cells. cDNA sequencing libraries were prepared according to the manufacturer's protocol and sequenced on an Illumina Hi-seq 4000 (2x50bp paired-end reads).

#### Processing FASTQ Files and Quality Control

Raw sequence reads in FASTQ format from fetal, paediatric and organoid samples were processed and aligned to the GRCh38-1.2.0 human reference transcriptome using the Cellranger v2.1.1 pipeline (10x Genomics) with default parameters.

The resulting gene expression matrices integrated together using Scanpy package v1.4 (Wolf et al., 2018). A total of 34 fetal sample count matrices were merged together. Separately, 15 gene expression matrices of healthy and CD paediatric biopsy samples were merged together for cell annotation and direct comparisons. Organoid gene expression matrices from the same experiment were also merged separately.

The pre-processing followed the guidelines provided by Scanpy V1.4 tutorial (Wolf et al., 2018). In short, entries with fewer than 200 genes and greater than 9000 total genes were filtered to remove empty droplets and probable doublets, respectively. The distribution of Unique Molecular Identifiers (UMIs) and genes per cell were visualised using scanpy pl.scatter function (Figures S1A–S1C). To account for differences in sequencing depth across samples, we normalised expression values for total UMIs per cell and log transformed the counts.

#### Doublet Removal

All 10x runs were processed using Scrublet doublet detection pipeline with threshold of 0.25-0.3 (Wolock et al., 2019), and predicted doublets were excluded from the analysis. We further annotated doublets by sub-clustering the data and identifying clusters with gene expression of other clusters. In fetal samples the doublets were largely epithelial-mesenchymal and neuronal-mesenchymal. In paediatric samples, we found mostly T cell-enterocyte and Goblet cell-enterocyte doublets.

#### Clustering, Visualisation and Cell Annotation

For cell clustering we used highly variable genes selected using sc.pp.highly_variable_genes function with default parameters. In addition, cell-cycle signatures were determined using cycle stage marker genes imported from human_cycle_markers.rds, scran package (Scialdone et al., 2015) and removed from highly variable genes of the full dataset. Similarly, ribosomal protein genes were removed from the highly variable genes as they contributed to the highest variability in the F6.1 sample. In addition, UMI counts, percentage of mitochondrial genes were considered to be the source of unwanted variability and were regressed using Scanpy regress_out function (Wolf et al., 2018).

To remove variation of each 10X Genomics run and maintain the development related biological variation, we used batch balanced k nearest neighbour (BBKNN) method (Polański et al., 2020) on 40 principal components and trim parameter set to 20. Dimensionality reduction was performed on remaining highly variable genes and cells were visualised using Uniform Manifold Approximation and Projection (UMAP) plots (Becht et al., 2018). We then used Scanpy implementation of Leiden algorithm for unsupervised clustering of the data (Traag et al., 2019). Clusters were annotated using markers genes found in the literature in combination with differentially expressed genes (Wilcoxon test, function sc.tl.rank_genes_groups). Paediatric healthy and CD samples were annotated together, in order to draw direct comparisons. Marker gene expression was visualised using dot-plots where the size of the dot reflects the percentage of cells expressing the gene and color indicates relative expression.

### scVelo and PAGA Trajectory Analysis

Fetal epithelial cell dynamics in small bowel samples were analysed using scVelo 1.24 package implementation in Scanpy 1.4.5 (Bergen et al., 2020; Svensson and Pachter, 2018). The data was sub-clustered to epithelial cells and split into two groups 6–8 PCW (including F6.7, F6.9, F.7.9), and 9-10 PCW (including F9.9, F10, F10.1, F10.2). The clustering and visualisation was repeated using the same parameters as above for the sub-clustered cells. The data was then processed using default parameters following preprocessing as described in Scanpy scVelo implementation.

In short, the gene-specific velocities are obtained by fitting a ratio between unspliced and spliced mRNA abundances and then computing how the observed abundances change from those observed in steady state. The ratio of 'spliced', 'unspliced', and 'ambiguous' transcripts was calculated to be 0.67, 0.25, 0.07, and 0.76, 0.17, 0.06 for 6-8 PCW and 9-10 PCW groups, respectively. The samples were pre-processed using functions for detection of minimum number of counts, filtering and normalisation using scv.pp.filter_and_normalise and followed by scv.pp.moments function. The gene specific velocities were then calculated using scv.tl.velocity with mode set to stochastic, and scv.tl.velocity_graph functions) and visualised using scv.pl.velocity_graph function. In addition, we used tl.recover_latent_time function to infer a shared latent time from splicing dynamics and plotted the genes along time axis sorted by expression along dynamics using scv.pl.heatmap function.

For Partition-based graph abstraction (PAGA) analysis (Wolf et al., 2019), we use scanpy implementation, sc.tl.draw_graph (init_pos='paga') followed by sc.tl.paga (threshold=0.3) and sc.pl.paga function for analysis and plotting, and similarly Scanpy scVelo implementation function scv.tl.paga with default parameters and scv.pl.paga function for velocity-driven paga analysis and plotting.

#### Inferring Cell-Cell Interactions

To infer cell-cell interactions we applied the CellPhoneDB v2.0 python package (Vento-Tormo et al., 2018; Efremova et al., 2020) to four separate datasets: 1) embryonic cells from duo-jejunum and ileum; 2) fetal cells from duo-jejunum and ileum; 3) healthy paediatric samples; and 4) CD samples. Log transformed and normalised counts, and cell type annotations were used as an input. To narrow down the most relevant interactions, we looked for specific interactions classified by ligand/receptor expression in more than 10% of cells within a cluster and where log_2_ mean expression of ligand/receptor pair is greater than 0.

#### Cellular Composition Classification

Raw counts for Kinchen et al. (2018) and Martin et al. (2019) were downloaded from GEO website and processed-annotated according to the original publication. The python package Sklearn implementation linear_model.LogisticRegression (Pedregosa et al., 2011) was used to predict the cellular composition and temporal identity of either the organoid datasets (Figures 5F and 5L) or pediatric datasets (Figure S6). In case of organoids, expression matrix and annotation labels of all primary cells from the developing small bowel were used as an input for training the model. For prediction of fetal stromal cells, the model was trained on healthy biopsy data from Kinchen et al. (2018). For pediatric data predictions, expression matrix and annotation labels of either healthy biopsy data from Kinchen et al. (2018), or healthy pediatric ileal cells from this study were used as an input for training the model. In all cases above, we used C=0.20, solver='saga' and penalty set to L1 to favour sparsity in the scRNAseq expression matrix. The classifier estimated sparsity was over 95% and lr.score was over 0.9. We took into account predictions with probability higher than 80% and used top labels to calculate relative abundance of predicted cell types. The relative abundance of predicted cell-types in organoids was shown as percentage of cells per experimental condition (p1 vs p17, or WNT3A- vs WNT3A+).

#### Transcription Factors in Epithelium

For comparisons between fetal and paediatric epithelium, we merged and analysed cells collected from the matching anatomical location (fetal terminal ileum only) and enriched using the same strategy. First, we selected all transcription factors (TF) based on a list obtained from (http://bioinfo.life.hust.edu.cn/AnimalTFDB/#!/download) and used Scanpy sc.tl.rank_genes_groups function (Wilcoxon test) to select differentially expressed TF (from total of 1529 expressed TF) between inflamed CD (Figure S5, arrows) versus control and non-inflamed epithelium (5F, bottom barplot, samples with no arrows). Out of these, we selected TFs that were differentially expressed in CD patients and upregulated in fetal epithelium and plotted their relative mean expression as a heatmap using sns.clustermap, z-score calculated for genes (rows).

#### Crypt-Villus Axis and HH Signalling Score

The axis score was derived by using the expression of selected crypt-villus axis markers as defined by Moor et al. (2018) and Parikh et al. (2019) (SEPP1, CEACAM7, PLAC8, CEACAM1, TSPAN1, CEACAM5, CEACAM6, IFI27, DHRS9, KRT20, RHOC, CD177, PKIB, HPGD, LYPD8, APOBEC1, APOB, APOA4, APOA1, NPC1L1, EGFR, KLF4, ENPP3, NT5E, SLC28A2, ADA). Similarly, we use selected genes for the scoring of HH pathway activation (*PTCH1*, *PTCH2*, *GLI1*, *GLI2*, *GLI3*, *SMO*). The scoring was done using sc.tl.score_genes() function with default parameters to calculate the average expression of selected genes substrated with the average expression of reference genes.

### Quantification and Statistical Analysis

#### Percentage of Cells and Statistical Analysis

First, we calculated relative abundance of each epithelial cell type as percentage of cells per condition (control and CD, fetal ileum and fetal duodenum). We test for statistical significance using two-tailed t.test (R version 3.5.0) and report the p-values as extracted by fdrtool package (statistics = p_values). To assess regional contribution to epithelial clusters, we used total number of cells and two-way ANOVA for multiple comparisons (GraphPad).

## Supplemental Information

Document S1. Figures S1–S6

Table S1. Sample Metadata, Related to Figure 1 Fetal (proximal small bowel n = 21,597, distal small bowel n = 21,147, large bowel n = 20,110), paediatric, and organoid single-cell sample metadata

Table S2. Media composition of human fetal organoids cultured in this study, Related to Figure 4

Document S2. Article plus Supplemental Information
