# Supplementary material for: Single-Cell Sequencing of Developing Human Gut Reveals Transcriptional Links to Childhood Crohn’s Disease
Source: Dev Cell. 2020 Dec 21;55(6):771–783.e5. doi: 10.1016/j.devcel.2020.11.010 (PMC7762816; doi:10.1016/j.devcel.2020.11.010)
Supplement: Document S1. Figures S1–S6 [file mmc1.pdf]

Developmental Cell, Volume *55*

Supplemental Information

Single-Cell Sequencing of Developing Human Gut Reveals Transcriptional Links to Childhood Crohn's Disease

Rasa Elmentaite, Alexander D.B. Ross, Kenny Roberts, Kylie R. James, Daniel Ortmann, Tomás Gomes, Komal Nayak, Liz Tuck, Sophie Pritchard, Omer Ali Bayraktar, Robert Heuschkel, Ludovic Vallier, Sarah A. Teichmann, and Matthias Zilbauer

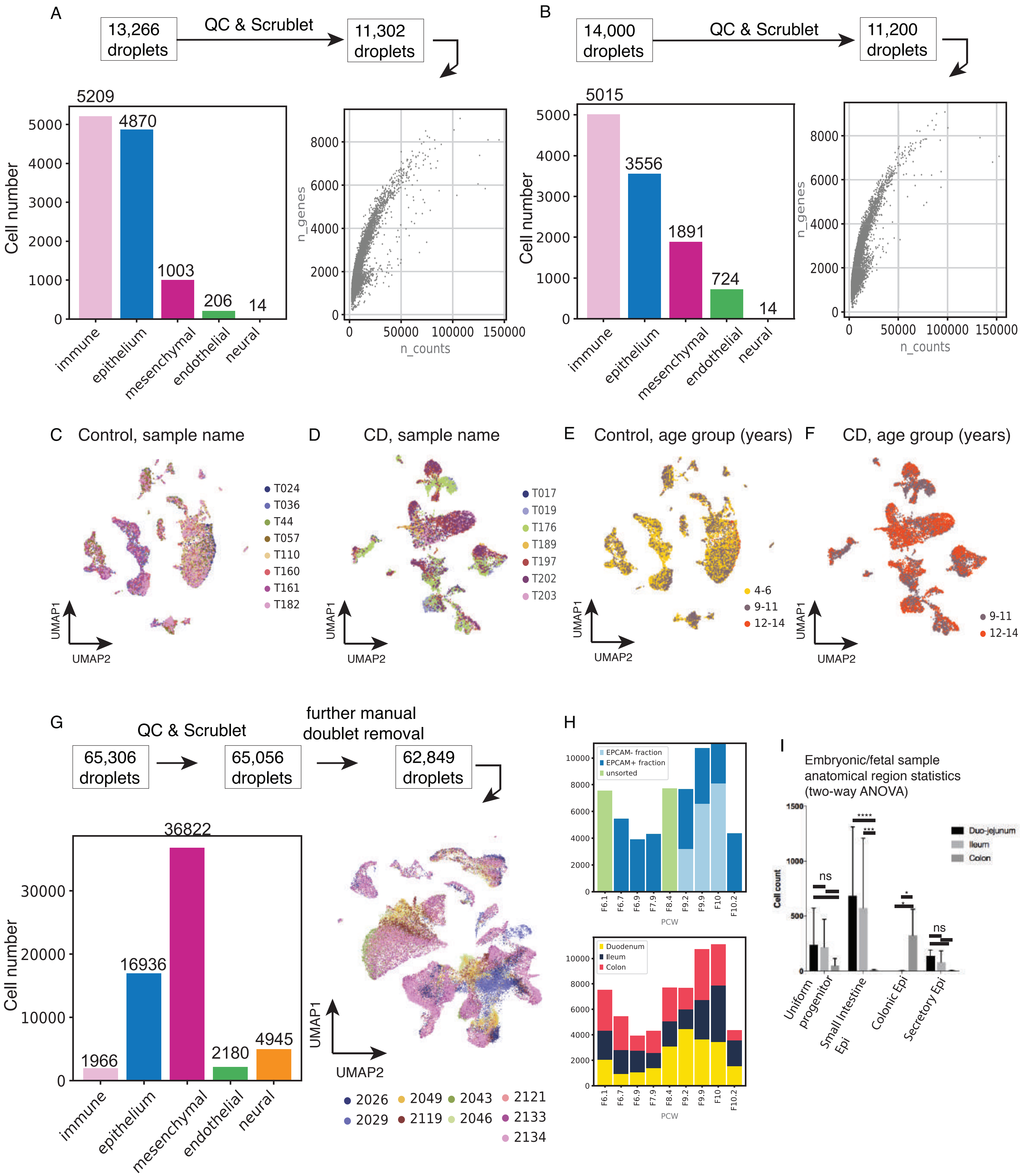
A
13,266 droplets
QC & Scrublet
11,302 droplets
Cell number
5209
4870
1003
206
14
immune
epithelium
mesenchymal
endothelial
neural
n_genes
n_counts
B
14,000 droplets
QC & Scrublet
11,200 droplets
5015
3556
1891
724
14
C Control, sample name
T024
T036
T44
T057
T110
T160
T161
T182
D CD, sample name
T017
T019
T176
T189
T197
T202
T203
E Control, age group (years)
4-6
9-11
12-14
F CD, age group (years)
9-11
12-14
UMAP1
UMAP2
G
QC & Scrublet
further manual doublet removal
65,306 droplets
65,056 droplets
62,849 droplets
36822
16936
1966
2180
4945
2026
2049
2043
2121
2029
2119
2046
2133
2134
H
EPCAM- fraction
EPCAM+ fraction
unsorted
Duodenum
Ileum
Colon
PCW
F6.1
F6.7
F6.9
F7.9
F8.4
F9.2
F9.9
F10
F10.2
I
Embryonic/fetal sample anatomical region statistics (two-way ANOVA)
Cell count
Duo-jejunum
Ileum
Colon
ns
****
***
*
Uniform progenitor
Small Intestine Epi
Colonic Epi
Secretory Epi

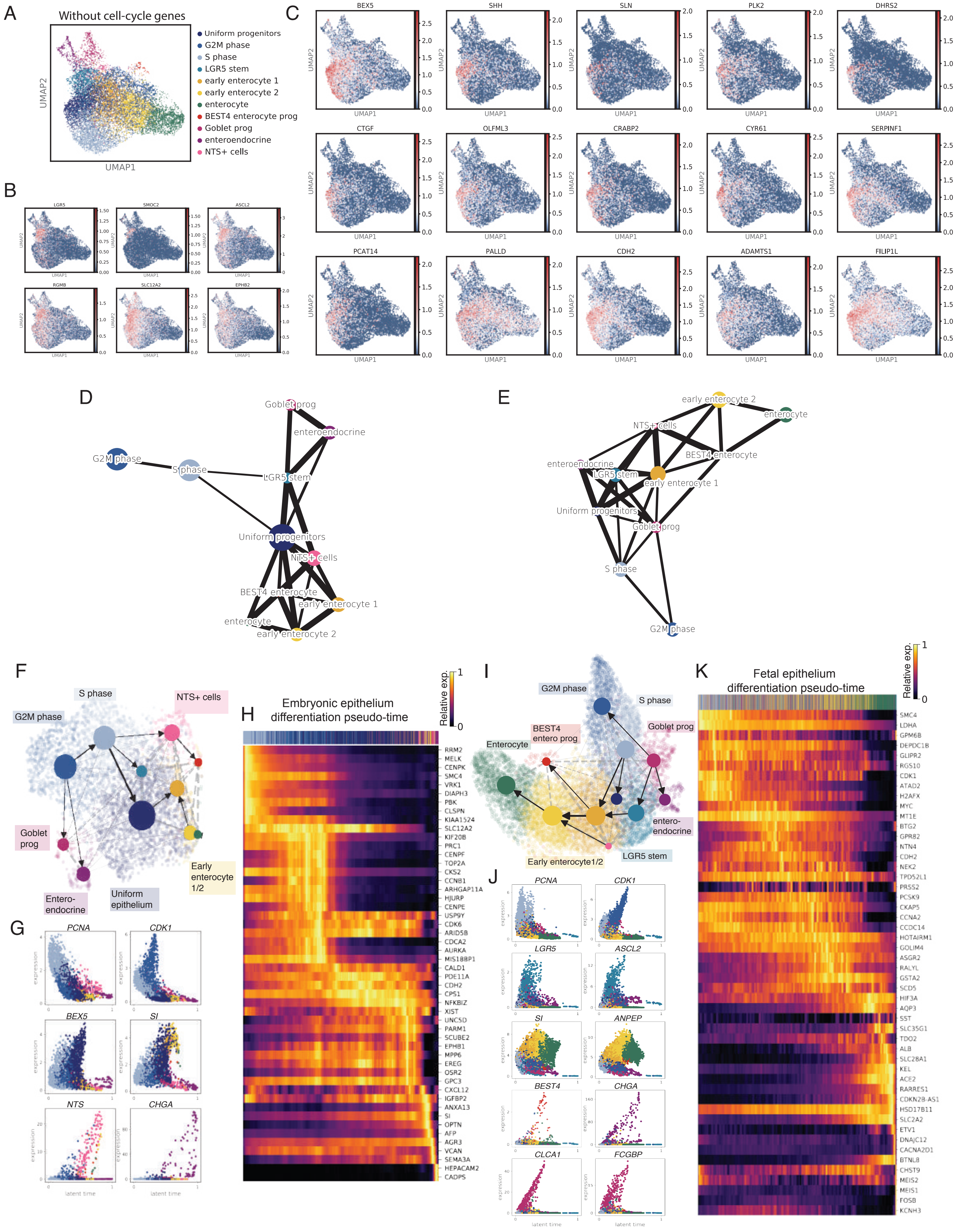

A
Without cell-cycle genes
Uniform progenitors
G2M phase
S phase
LGR5 stem
early enterocyte 1
early enterocyte 2
enterocyte
BEST4 enterocyte prog
Goblet prog
enteroendocrine
NTS+ cells
B
LGR5
SMOC2
ASCL2
RGMB
SLC12A2
EPHB2
C
BEX5
SHH
SLN
PLK2
DHRS2
CTGF
OLFML3
CRABP2
CYR61
SERPINF1
PCAT14
PALLD
CDH2
ADAMTS1
FILIP1L
D
E
F
G
PCNA
CDK1
BEX5
SI
NTS
CHGA
latent time
H
Embryonic epithelium differentiation pseudo-time
Relative exp.
RRM2
MELK
CENPK
SMC4
VRK1
DIAPH3
PBK
CLSPN
KIAA1524
SLC12A2
KIF20B
PRC1
CENPF
TOP2A
CKS2
CCNB1
ARHGAP11A
HJURP
CENPE
USP9Y
CDK6
ARID5B
CDCA2
AURKA
MIS18BP1
CALD1
PDE11A
CDH2
CPS1
NFKBIZ
XIST
UNC5D
PARM1
SCUBE2
EPHB1
MPP6
EREG
OSR2
GPC3
CXCL12
IGFBP2
ANXA13
SI
OPTN
AFP
AGR3
VCAN
SEMA3A
HEPACAM2
CADPS
I
G2M phase
S phase
BEST4 entero prog
Goblet prog
Enterocyte
entero-endocrine
LGR5 stem
Early enterocyte1/2
J
PCNA
CDK1
LGR5
ASCL2
SI
ANPEP
BEST4
CHGA
CLCA1
FCGBP
K
Fetal epithelium differentiation pseudo-time
SMC4
LDHA
GPM6B
DEPDC1B
GLIPR2
RGS10
CDK1
ATAD2
H2AFX
MYC
MT1E
BTG2
GPR82
NTN4
CDH2
NEK2
TPD52L1
PRSS2
PCSK9
CKAP5
CCNA2
CCDC14
HOTAIRM1
GOLIM4
ASGR2
RALYL
GSTA2
SCD5
HIF3A
AQP3
SST
SLC35G1
TDO2
ALB
SLC28A1
KEL
ACE2
RARRES1
CDKN2B-AS1
HSD17B11
SLC2A2
ETV1
DNAJC12
CACNA2D1
BTNL8
CHST9
MEIS2
MEIS1
FOSB
KCNH3

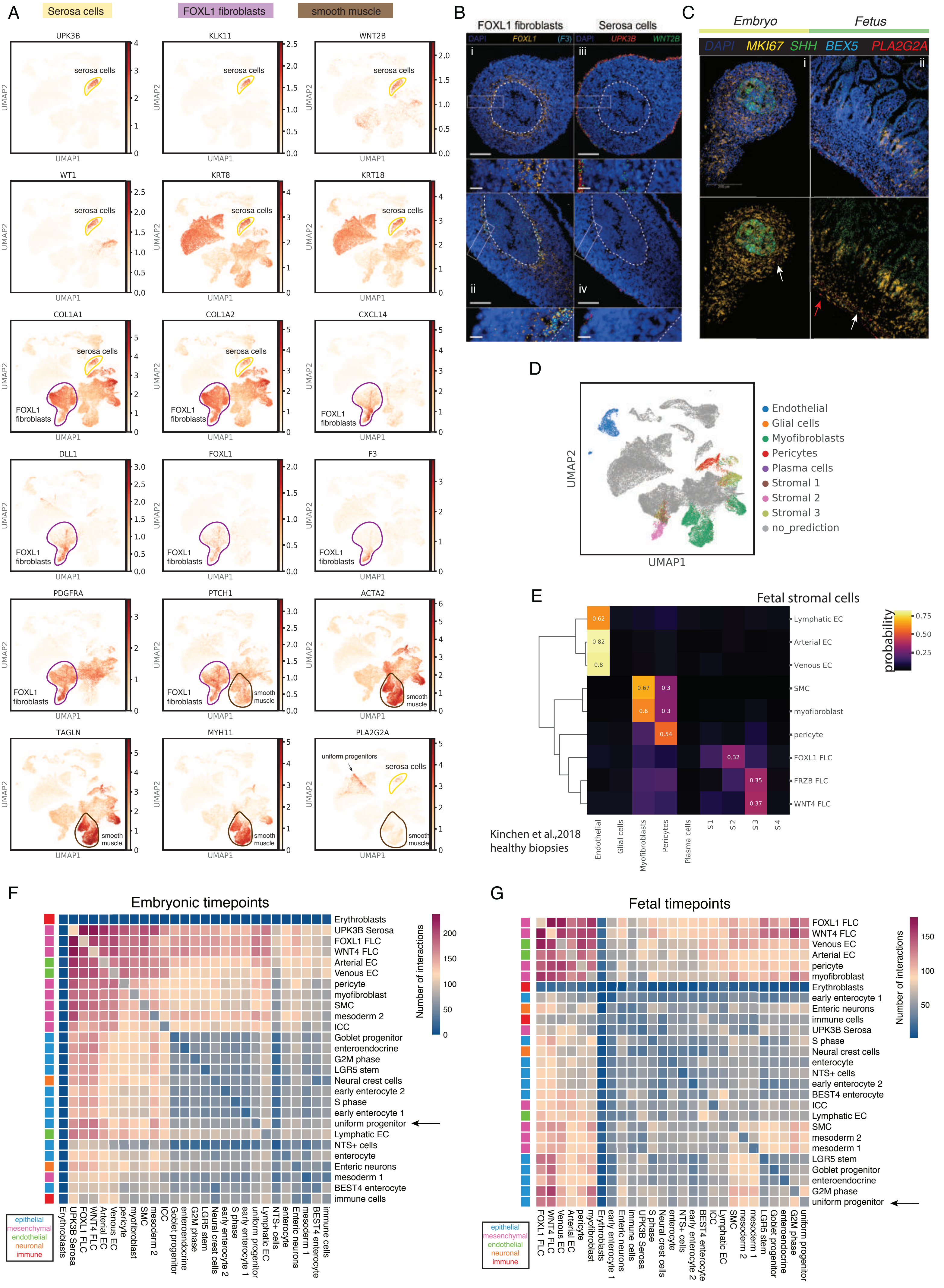

A
Serosa cells
FOXL1 fibroblasts
smooth muscle
UPK3B
KLK11
WNT2B
WT1
KRT8
KRT18
COL1A1
COL1A2
CXCL14
DLL1
FOXL1
F3
PDGFRA
PTCH1
ACTA2
TAGLN
MYH11
PLA2G2A
serosa cells
FOXL1 fibroblasts
smooth muscle
uniform progenitors
UMAP1
UMAP2
B
FOXL1 fibroblasts
Serosa cells
DAPI FOXL1 (F3)
DAPI UPK3B WNT2B
i
ii
iii
iv
C
Embryo
Fetus
DAPI MKI67 SHH BEX5 PLA2G2A
D
Endothelial
Glial cells
Myofibroblasts
Pericytes
Plasma cells
Stromal 1
Stromal 2
Stromal 3
no_prediction
UMAP1
UMAP2
E
Fetal stromal cells
Lymphatic EC
Arterial EC
Venous EC
SMC
myofibroblast
pericyte
FOXL1 FLC
FRZB FLC
WNT4 FLC
0.62
0.82
0.8
0.67
0.3
0.6
0.3
0.54
0.32
0.35
0.37
Endothelial
Glial cells
Myofibroblasts
Pericytes
Plasma cells
S1
S2
S3
S4
probability
0.75
0.50
0.25
Kinchen et al.,2018
healthy biopsies
F
Embryonic timepoints
Erythroblasts
UPK3B Serosa
FOXL1 FLC
WNT4 FLC
Arterial EC
Venous EC
pericyte
myofibroblast
SMC
mesoderm 2
ICC
Goblet progenitor
enteroendocrine
G2M phase
LGR5 stem
Neural crest cells
early enterocyte 2
S phase
early enterocyte 1
uniform progenitor
Lymphatic EC
NTS+ cells
enterocyte
Enteric neurons
mesoderm 1
BEST4 enterocyte
immune cells
Number of interactions
200
150
100
50
0
epithelial
mesenchymal
endothelial
neuronal
immune
G
Fetal timepoints
FOXL1 FLC
WNT4 FLC
Venous EC
Arterial EC
pericyte
myofibroblast
Erythroblasts
early enterocyte 1
Enteric neurons
immune cells
UPK3B Serosa
S phase
Neural crest cells
enterocyte
NTS+ cells
early enterocyte 2
BEST4 enterocyte
ICC
Lymphatic EC
SMC
mesoderm 2
mesoderm 1
LGR5 stem
Goblet progenitor
enteroendocrine
G2M phase
uniform progenitor
Number of interactions
150
100
50
epithelial
mesenchymal
endothelial
neuronal
immune

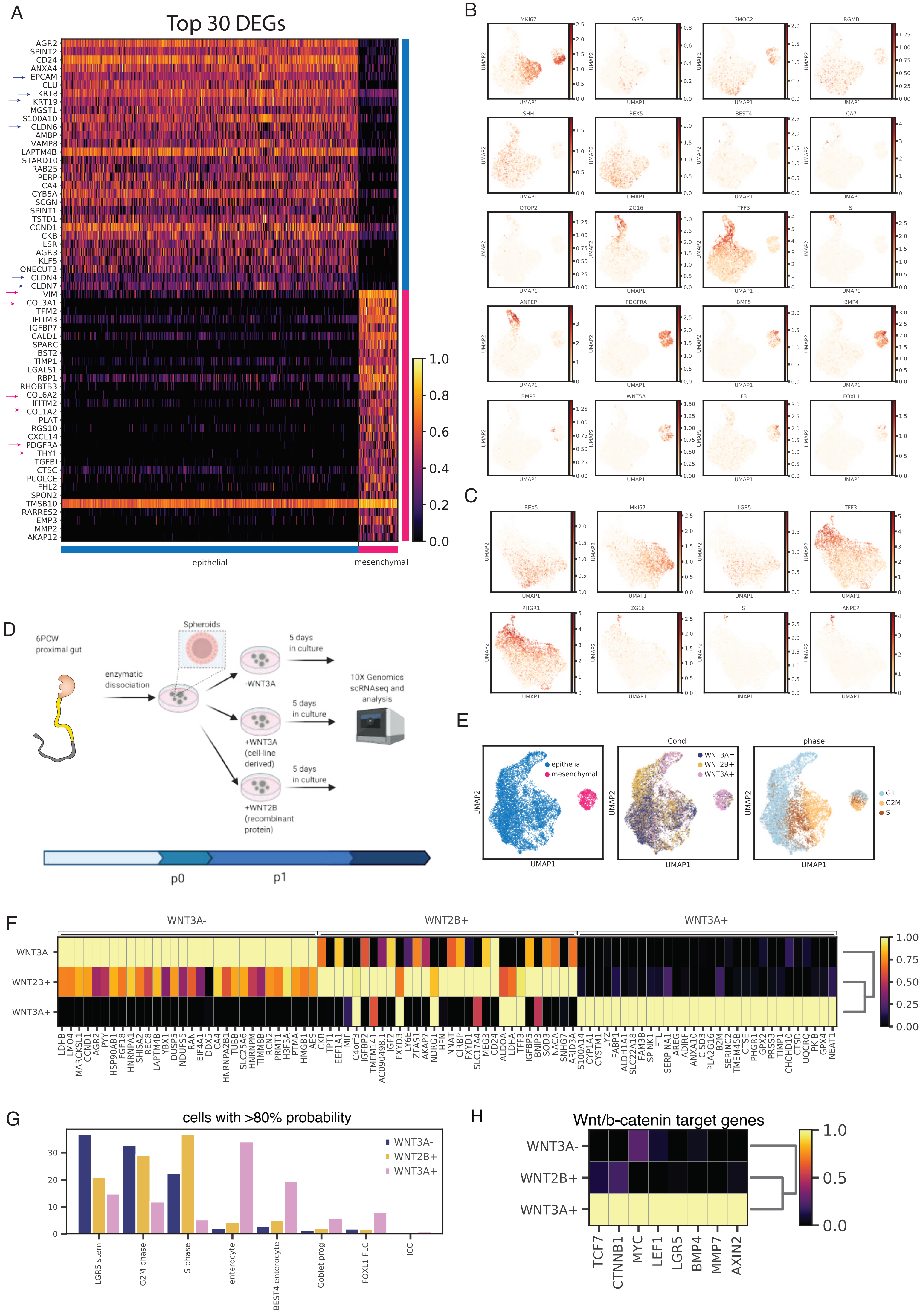
A
Top 30 DEGs
epithelial
mesenchymal
B
C
D
6PCW proximal gut
enzymatic dissociation
Spheroids
5 days in culture
-WNT3A
+WNT3A (cell-line derived)
+WNT2B (recombinant protein)
10X Genomics scRNAseq and analysis
p0
p1
E
epithelial
mesenchymal
Cond
WNT3A−
WNT2B+
WNT3A+
phase
G1
G2M
S
UMAP1
UMAP2
F
WNT3A-
WNT2B+
WNT3A+
G
cells with >80% probability
LGR5 stem
G2M phase
S phase
enterocyte
BEST4 enterocyte
Goblet prog
FOXL1 FLC
ICC
H
Wnt/b-catenin target genes
TCF7
CTNNB1
MYC
LEF1
LGR5
BMP4
MMP7
AXIN2

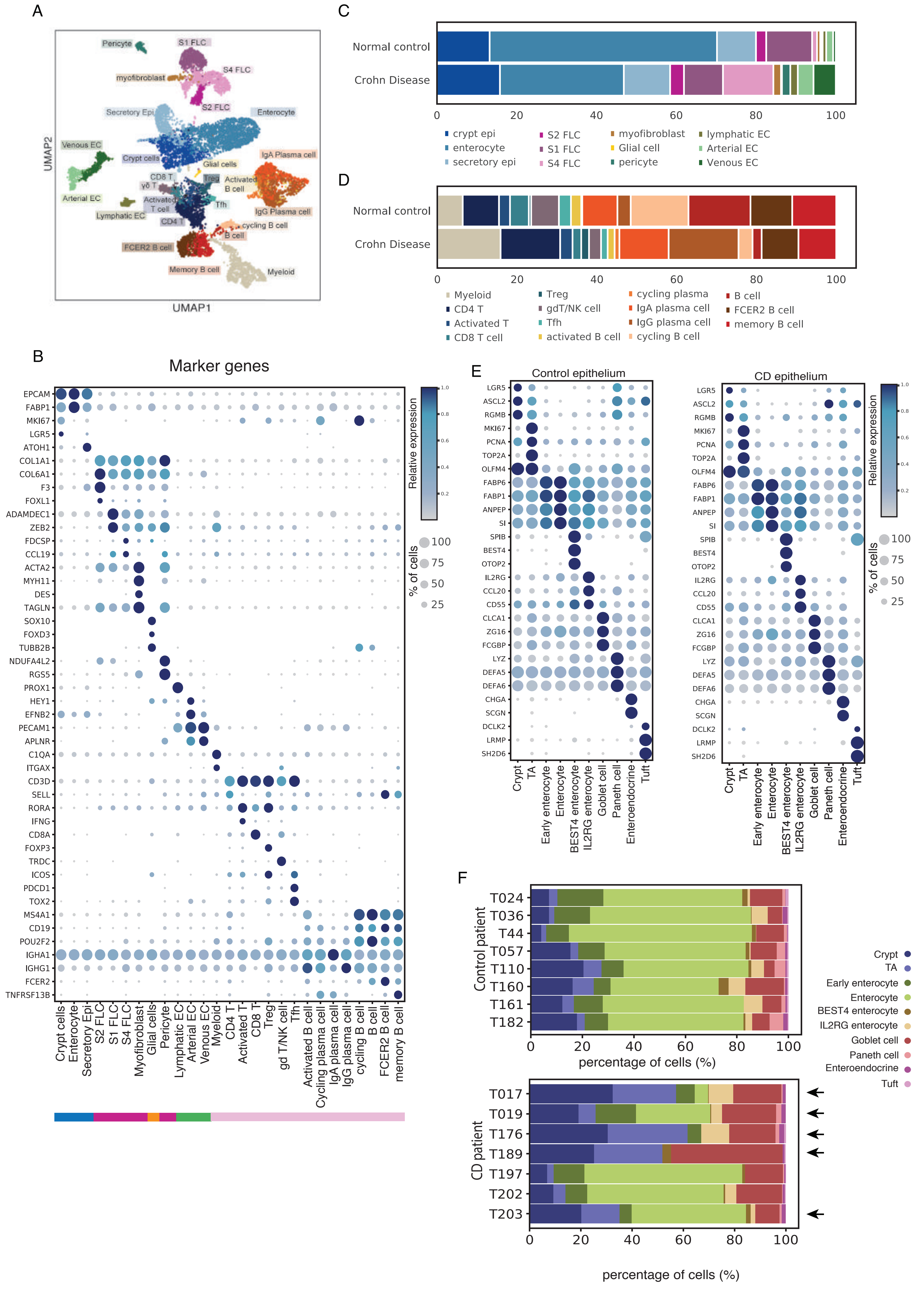
A
Pericyte
S1 FLC
S4 FLC
myofibroblast
S2 FLC
Secretory Epi
Enterocyte
Venous EC
Crypt cells
Glial cells
IgA Plasma cell
CD8 T
γδ T
Treg
Activated B cell
Arterial EC
Activated T cell
Tfh
Lymphatic EC
CD4 T
IgG Plasma cell
cycling B cell
FCER2 B cell
Memory B cell
Myeloid
UMAP1
UMAP2
C
Normal control
Crohn Disease
0 20 40 60 80 100
crypt epi
enterocyte
secretory epi
S2 FLC
S1 FLC
S4 FLC
myofibroblast
Glial cell
pericyte
lymphatic EC
Arterial EC
Venous EC
D
Normal control
Crohn Disease
0 20 40 60 80 100
Myeloid
CD4 T
Activated T
CD8 T cell
Treg
gdT/NK cell
Tfh
activated B cell
cycling plasma
IgA plasma cell
IgG plasma cell
cycling B cell
B cell
FCER2 B cell
memory B cell
B
Marker genes
EPCAM
FABP1
MKI67
LGR5
ATOH1
COL1A1
COL6A1
F3
FOXL1
ADAMDEC1
ZEB2
FDCSP
CCL19
ACTA2
MYH11
DES
TAGLN
SOX10
FOXD3
TUBB2B
NDUFA4L2
RGS5
PROX1
HEY1
EFNB2
PECAM1
APLNR
C1QA
ITGAX
CD3D
SELL
RORA
IFNG
CD8A
FOXP3
TRDC
ICOS
PDCD1
TOX2
MS4A1
CD19
POU2F2
IGHA1
IGHG1
FCER2
TNFRSF13B
Crypt cells
Enterocyte
Secretory Epi
S2 FLC
S1 FLC
S4 FLC
Myofibroblast
Glial cells
Pericyte
Lymphatic EC
Arterial EC
Venous EC
Myeloid
CD4 T
Activated T
CD8 T
Treg
gd T/NK cell
Tfh
Activated B cell
Cycling plasma cell
IgA plasma cell
IgG plasma cell
cycling B cell
B cell
FCER2 B cell
memory B cell
Relative expression
% of cells
100
75
50
25
E
Control epithelium
CD epithelium
LGR5
ASCL2
RGMB
MKI67
PCNA
TOP2A
OLFM4
FABP6
FABP1
ANPEP
SI
SPIB
BEST4
OTOP2
IL2RG
CCL20
CD55
CLCA1
ZG16
FCGBP
LYZ
DEFA5
DEFA6
CHGA
SCGN
DCLK2
LRMP
SH2D6
Crypt
TA
Early enterocyte
Enterocyte
BEST4 enterocyte
IL2RG enterocyte
Goblet cell
Paneth cell
Enteroendocrine
Tuft
Relative expression
% of cells
100
75
50
25
F
Control patient
T024
T036
T44
T057
T110
T160
T161
T182
0 20 40 60 80 100
percentage of cells (%)
CD patient
T017
T019
T176
T189
T197
T202
T203
0 20 40 60 80 100
percentage of cells (%)
Crypt
TA
Early enterocyte
Enterocyte
BEST4 enterocyte
IL2RG enterocyte
Goblet cell
Paneth cell
Enteroendocrine
Tuft

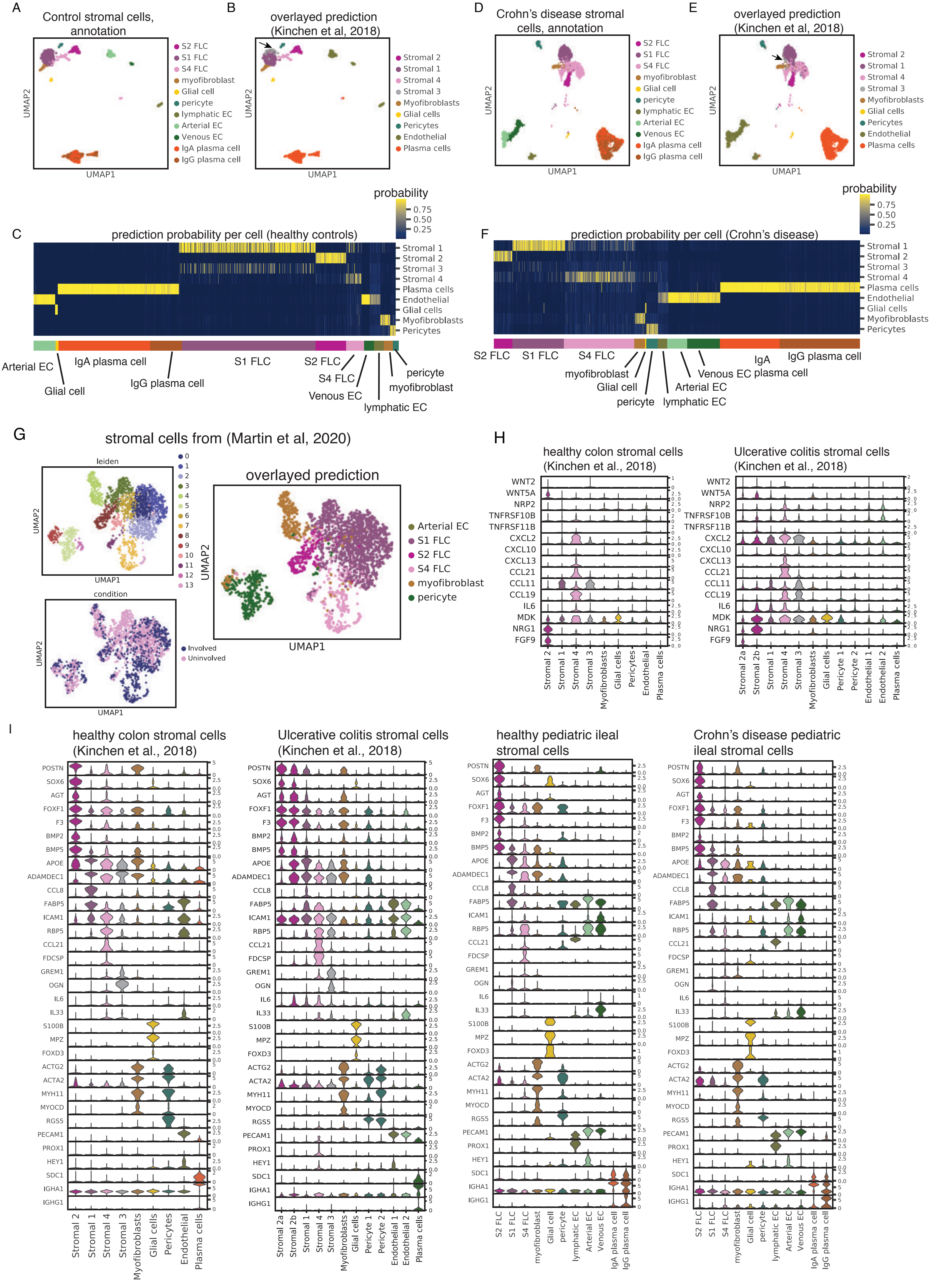
A
Control stromal cells, annotation
S2 FLC
S1 FLC
S4 FLC
myofibroblast
Glial cell
pericyte
lymphatic EC
Arterial EC
Venous EC
IgA plasma cell
IgG plasma cell
UMAP1
UMAP2
B
overlayed prediction (Kinchen et al, 2018)
Stromal 2
Stromal 1
Stromal 4
Stromal 3
Myofibroblasts
Glial cells
Pericytes
Endothelial
Plasma cells
D
Crohn's disease stromal cells, annotation
E
overlayed prediction (Kinchen et al, 2018)
probability
0.75
0.50
0.25
C
prediction probability per cell (healthy controls)
Arterial EC
IgA plasma cell
IgG plasma cell
Glial cell
S1 FLC
S2 FLC
S4 FLC
Venous EC
lymphatic EC
pericyte
myofibroblast
F
prediction probability per cell (Crohn's disease)
IgA plasma cell
IgG plasma cell
G
stromal cells from (Martin et al, 2020)
leiden
condition
Involved
Uninvolved
overlayed prediction
H
healthy colon stromal cells (Kinchen et al., 2018)
Ulcerative colitis stromal cells (Kinchen et al., 2018)
WNT2
WNT5A
NRP2
TNFRSF10B
TNFRSF11B
CXCL2
CXCL10
CXCL13
CCL21
CCL11
CCL19
IL6
MDK
NRG1
FGF9
Stromal 2a
Stromal 2b
Pericyte 1
Pericyte 2
Endothelial 1
Endothelial 2
I
healthy colon stromal cells (Kinchen et al., 2018)
Ulcerative colitis stromal cells (Kinchen et al., 2018)
healthy pediatric ileal stromal cells
Crohn's disease pediatric ileal stromal cells
POSTN
SOX6
AGT
FOXF1
F3
BMP2
BMP5
APOE
ADAMDEC1
CCL8
FABP5
ICAM1
RBP5
CCL21
FDCSP
GREM1
OGN
IL6
IL33
S100B
MPZ
FOXD3
ACTG2
ACTA2
MYH11
MYOCD
RGS5
PECAM1
PROX1
HEY1
SDC1
IGHA1
IGHG1

Figure S1. Related to Figures 1 and 2. Quality control of the three datasets. A) and B) Bar plots with a number of droplets after QC and Scrublet doublet exclusion grouped by cell type group. Scatter plots show the number of genes over the number of counts per cell, where each dot is a cell. Sample contribution to each cluster in C) Control and D) CD datasets. UMAP plots colored by sample age group in E) Control and F) CD samples. G) Bar plots with number of cells in fetal dataset and UMAP with sample contribution to each cluster. H) Number of fetal cells captured in each timepoint colored by enrichment strategy (top) and region (bottom). I) Number of cells of broad epithelial cell clusters (Uniform progenitor, Small Intestine Epi, Colonic Epi, Secretory Epi) in each of three developing anatomical regions (duo-jejunum, ileum and colon). Epi = epithelium. For fetal samples, the average cell recovery was 1,800 with a total of 62,849 cells at a mean depth of 13, 570 reads per cell and 3,027 mean genes per cell. For paediatric samples, the average recovery was 1,400 cells with 8,093 reads per cell and 1,859 mean genes per cell. CD = Crohn's disease. Fetal sample ages in post-conception weeks were as follows: BRC2029 - 6.1, BRC2026 - 8.4, BRC2043 - 10.2, BRC2046 - 6.7, BRC2049- 6.9, BRC2121 - 9.2, BRC2119- 7.9, BRC2133 - 9.9, BRC2134- 10. Patient ages were as follows: Control group (T036- 4 years, T110- 4 years, T161- 4 years, T057- 6 years, T182- 9 years, T44- 10 years, T024- 12 years, T160- 10 years);

Figure S2. Related to Figure 3. Marker genes and trajectory analysis of embryonic and fetal intestinal epithelium. A) UMAP projection of cells after cell-cycle gene exclusion from gene list and dimensionality reduction. The cells are coloured by their cell type annotation. B) Gene expression of adult-like LGR5 stem cell genes. C) Feature plots with selected differentially expressed genes in uniform progenitors. prog = progenitors. PAGA graphs (D and E) and velocity-driven paga graphs (F and I) for embryonic and fetal epithelial cells, respectively. prog = progenitor. PAGA graphs can illustrate either connectivities (solid/dashed lines) or transitions (arrows), where the latter are defined by scVelo. G) and J) Differentiating cell type marker gene expression along the pseudotime (latent time). Cells are colored by the cell type as in UMAP. *PCNA*, *CDK1*- S and G2M phase cells; *BEX5*- uniform progenitors; *LGR5*, *ASCL2*- LGR5 stem cells; *SI*, *ANPEP* - enterocytes; *CHGA*- enteroendocrine cells; *NTS*- NTS+ cells; *BEST4*- BEST4 enterocytes; *CLACA1*, *FCGB*P- goblet cells. H) and K) Top 50 driver genes that display pronounced dynamic behaviour along the pseudotime in embryonic and fetal epithelium, respectively. Top bars in H) and K) represent cell annotation and their colors match annotation colors in F) and I), respectively. prog= progenitor.

Figure S3. Related to Figure 4. Cell-cell interactions in embryonic and fetal gut. A) Feature plots with selected genes expressed in three stromal populations in embryonic and fetal gut. Circled populations are serosal cells (yellow), FOXL1 fibroblasts (purple), smooth muscle cells (brown). Arrow points to uniform progenitors. B) smFISH images of serosa mesothelial cells and FOXL1 fibroblasts. i and ii in B) are two biological replicates imaging location of FOXL1 fibroblasts using DAPI, *FOXL1*, and *F3* (stained only in ii), while iii and iv are two biological replicates for locating Serosa mesothelial cells using co-expression of DAPI, *UPK3B*, and *WNT2B*. Scale bar main panel = 100 µm, zoom panel = 50 µm. C) smFISH images of cycling cells (*MKI67*), epithelial cells (*SHH* and *BEX5*), and *PLA2G2A* gene that is expressed in multiple populations in the gut. White arrows point to *PLA2G2A*-expressing SMCs, red arrow points to *PLA2G2A*-expressing UPK3B Serosa cells. Scale bar main panel = 100 µm, zoom panel = 50 µm. D) UMAP with overlaid predictions of fetal cells. Predictions were made using logistic regression models trained on adult stromal cells from (Kinchen *et al.*, 2018). E) Heatmap with mean prediction probability in stromal cell clusters. Heatmaps with number of interactions in embryonic (F) and fetal (G) samples as quantified using CellphoneDB v2.0. Color legend indicates the cell type lineage (epithelial-blue, mesenchymal-pink, endothelial-green, neuronal-orange, immune/erythroblast-red) of each plotted cell type. Arrows point to cell-types discussed in text. FLC = fibroblasts, EC = endothelial cells, ICC = interstitial cells of Cajal, S= stromal, SMC = smooth muscle cells.

Figure S4. Related to Figure 5. Marker gene expression in fetal intestinal organoids and fetal intestinal organoid co-culture with recombinant WNT2B. A) Heatmap of top 30 differentially expressed genes (DEGs) between mesenchymal and epithelial cells captured in the intestinal organoid culture. The arrows point to key genes associated with either mesenchymal (pink) or epithelial (blue) identity. B) Feature plot with mesenchymal and epithelial marker gene expression in WNT3A- and WNT3A+ organoids. C) Feature plot of epithelial marker genes expressed in p1 and p17 organoid cells. *MKI67*- cycling cells; *LGR5, SMOC2, RGM*B- LGR5 stem cells; *SHH, BEX5*- uniform progenitors; *BEST4, CA7, OTOP2*- BEST4 enterocytes; *ZG16, TFF3*- goblet cells; *SI, ANPEP, PHGR1* - enterocytes; *PDGFRA, BMP3, BMP4, BMP5, WNT5A, F3, FOXL1*- FOXL1 fibroblasts; D) Schematic with experimental strategy for organoid culture comparison between WNT2B (recombinant protein) and WNT3A (cell-line derived). The organoids were generated from dissociated cells of embryonic proximal ileum, cultured in three different culture conditions and sequenced on the day 5 of passage 1. E) UMAP visualisation of integrated organoid cells from three conditions (WNT3A- or no WNT, WNT2B+, WNT3A+). The UMAPs are colored by cluster identity (left), culture condition (middle), or cell cycle phase score (right). F) Top 30 differentially expressed genes in the epithelial organoid cells between three culture conditions. G) abundance of cells as predicted with logistic regression with more than 80% probability of matching transcriptionally. H) Relative expression of key canonical WNT pathway genes in the epithelium of three culture conditions. PCW = post-conception weeks.

Figure S5. Related to Figure 6. Crohn's disease patient dataset. A) UMAP projection of CD samples colored by cell type annotation. B) Dot plot with relative expression and percentage of cells expressing marker genes in paediatric CD dataset. C) and D) Bar plots with percentage of cells in CD dataset as compared with healthy paediatric (Control) dataset, grouped by the broad cell group (stromal =epithelial, mesenchymal, endothelial and glial, and immune = myeloid, T cells and B cells). E) Dot plots with relative expression and percentage of cells expressing marker genes in paediatric healthy (left) and CD (right) epithelium. F) Barplots show abundance of epithelial cell subsets in healthy controls (top, n=8) and children with CD (bottom, n=7). Arrows point to samples that were grouped as "inflamed Crohn's disease". CD = Crohn's disease.

Figure S6. Related to Figure 6. Pediatric data comparisons with published single-cell datasets. UMAP projections of stromal cells from healthy pediatric dataset colored by A) original cell type annotation defined in this study and B) overlaid predicted annotation generated by using a logistic regression model trained on scRNAseq data from Kinchen et al. 2018. arrows point to cells predicted to be Stromal 3 cell type. The prediction probabilities for each cell are shown in heatmap C). X-axis shows predicted cell type labels as in Kinchen et al. (2018) and y-axis is grouped by the original annotation as in A). The same logistic regression analysis was done for CD samples and results are shown in UMAPs with C) original annotation, D) overlaid predicted annotations (arrows point to cells predicted to be Stromal 3 cell type), and probability heatmap F). G) The comparison of ileal stromal cells from (Martin *et al.*, 2019) UMAP plots show ileal stromal cells from (Martin *et al.*, 2019).
