# Supplementary material for: Single-Cell Sequencing of Developing Human Gut Reveals Transcriptional Links to Childhood Crohn’s Disease
Source: Dev Cell. 2020 Dec 21;55(6):771–783.e5. doi: 10.1016/j.devcel.2020.11.010 (PMC7762816; doi:10.1016/j.devcel.2020.11.010)
Supplement: Document S2. Article plus Supplemental Information [file mmc4.pdf]

Developmental Cell

Resource

Single-Cell Sequencing of Developing Human Gut Reveals Transcriptional Links to Childhood Crohn's Disease

Graphical Abstract

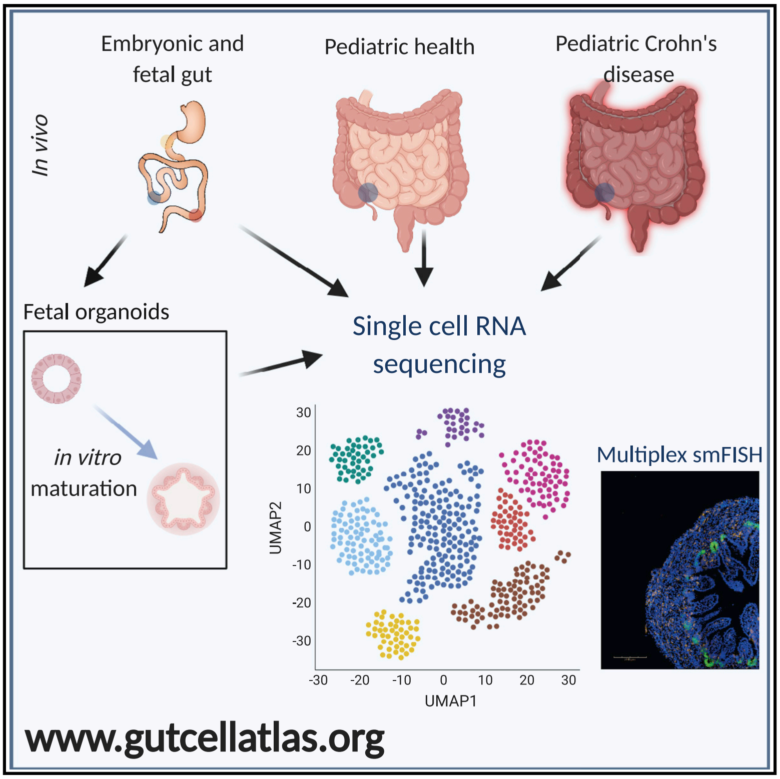

Authors

Rasa Elmentaite, Alexander D.B. Ross, Kenny Roberts, ..., Ludovic Vallier, Sarah A. Teichmann, Matthias Zilbauer

Correspondence

st9@sanger.ac.uk (S.A.T.),
mz304@medschl.cam.ac.uk (M.Z.)

In Brief

Elmentaite and Ross et al. generated a detailed single-cell RNA-seq map of the developing human intestine. Data analyses revealed the presence of an early intestinal epithelial precursor cell and the re-activation of fetal transcription factors in the intestinal epithelium of children diagnosed with Crohn's disease. The study provides a unique resource accessible to all researchers at www.gutcellatlas.org.

Highlights

- Single-cell RNA-seq map of the developing and pediatric human intestine
- Cycling BEX5+ epithelial precursors are distinct from adult LGR5+ stem cells
- Human fetal intestinal organoids mature in culture
- Fetal transcription factors are reactivated in the Crohn's disease epithelium

Elmentaite et al., 2020, Developmental Cell *55*, 771–783
December 21, 2020 © 2020 The Authors. Published by Elsevier Inc.
https://doi.org/10.1016/j.devcel.2020.11.010

Developmental Cell

CellPress OPEN ACCESS

Resource

Single-Cell Sequencing of Developing Human Gut Reveals Transcriptional Links to Childhood Crohn's Disease

Rasa Elmentaite,[1,8] Alexander D.B. Ross,[2,3,4,8] Kenny Roberts,[1] Kylie R. James,[1] Daniel Ortmann,[2,3] Tomás Gomes,[1] Komal Nayak,[4] Liz Tuck,[1] Sophie Pritchard,[1] Omer Ali Bayraktar,[1] Robert Heuschkel,[7] Ludovic Vallier,[2,3] Sarah A. Teichmann,[1,5,6,8,*] and Matthias Zilbauer[2,4,7,8,9,*]

[1]Wellcome Sanger Institute, Wellcome Genome Campus, Hinxton CB10 1SA, UK
[2]Wellcome Trust, MRC Cambridge Stem Cell Institute, University of Cambridge, Cambridge CB2 0SZ, UK
[3]Department of Surgery, University of Cambridge, Cambridge CB2 0QQ, UK
[4]Department of Paediatrics, University of Cambridge, Cambridge CB2 0QQ, UK
[5]Theory of Condensed Matter, Cavendish Laboratory, Department of Physics, University of Cambridge, Cambridge CB3 0HE, UK
[6]European Molecular Biology Laboratory, European Bioinformatics Institute (EBI), Wellcome Genome Campus, Hinxton CB10 1SA, UK
[7]Department of Paediatric Gastroenterology, Hepatology and Nutrition, Cambridge University Hospitals Trust, Cambridge CB2 0QQ, UK
[8]These authors contributed equally
[9]Lead Contact
*Correspondence: st9@sanger.ac.uk (S.A.T.), mz304@medschl.cam.ac.uk (M.Z.)
https://doi.org/10.1016/j.devcel.2020.11.010

SUMMARY

Human gut development requires the orchestrated interaction of differentiating cell types. Here, we generate an in-depth single-cell map of the developing human intestine at 6–10 weeks post-conception. Our analysis reveals the transcriptional profile of cycling epithelial precursor cells; distinct from LGR5-expressing cells. We propose that these cells may contribute to differentiated cell subsets via the generation of LGR5-expressing stem cells and receive signals from surrounding mesenchymal cells. Furthermore, we draw parallels between the transcriptomes of *ex vivo* tissues and *in vitro* fetal organoids, revealing the maturation of organoid cultures in a dish. Lastly, we compare scRNA-seq profiles from pediatric Crohn's disease epithelium alongside matched healthy controls to reveal disease-associated changes in the epithelial composition. Contrasting these with the fetal profiles reveals the re-activation of fetal transcription factors in Crohn's disease. Our study provides a resource available at www.gutcellatlas.org, and underscores the importance of unraveling fetal development in understanding disease.

INTRODUCTION

Development of the human intestine is a highly complex process that requires synergy between a wide range of cell types. Subtle differences between humans and mice (Chin et al., 2017; Yanai et al., 2017) combined with a limited access to human fetal and embryonic tissues, has rendered our understanding of these processes in humans rudimentary. Importantly, environmentally triggered alterations in early development have been implicated in a range of immune-mediated pathologies, including inflammatory bowel diseases (IBD) (Sonntag et al., 2007; Cilieborg et al., 2012; Dupaul-Chicoine et al., 2013; Kraiczy et al., 2016). Furthermore, a number of studies have reported a link between early fetal intestinal epithelial cell dynamics and IBD (Kraiczy et al., 2016; Yui et al., 2018; Wang et al., 2019), suggesting that fetal-like transcriptional programs may re-appear in the intestinal epithelium of IBD patients. Hence, deciphering physiological intestinal development is a critical step toward prevention and treatment of such conditions.

The human intestinal tract develops from the endodermal germ cell layer of the embryo, beginning with the formation of a simple tube at 3–4 weeks post-conception (PCW). Prior to villus formation, the intestinal epithelium, forming the innermost lining of the gut tube, is pseudostratified and is globally proliferative (Grosse et al., 2011). By the end of the first trimester (12 PCW), regionalization of the intestinal tube occurs and a crypt-villus axis starts to appear. While little is known about the villus formation in humans, two mechanisms have been proposed in model organisms: mesenchymal clustering in mice and the force generated by smooth muscle in chickens (Karlsson et al., 2000; Walton et al., 2012, 2016a, 2016b; Shyer et al., 2013). While there are significant differences between the two models, both employ similar signaling, including gradients of hedgehog (HH), PDGF, and bone morphogenetic protein (BMP) ligands (Kolterud et al., 2009; Korinek et al., 1998; Kurahashi et al., 2008; Madison et al., 2005; Geske et al., 2008; Shyer et al., 2015).

In the adult intestine, LGR5 is a marker of stem cells that reside at the bottom of intestinal crypts and give rise to all epithelial cell subsets (Barker et al., 2007). The ability to generate self-organizing intestinal epithelial organoids from fetal gut epithelium as early as 8–10 PCW implies the presence of these LGR5+

This is an open access article under the CC BY license (http://creativecommons.org/licenses/by/4.0/).

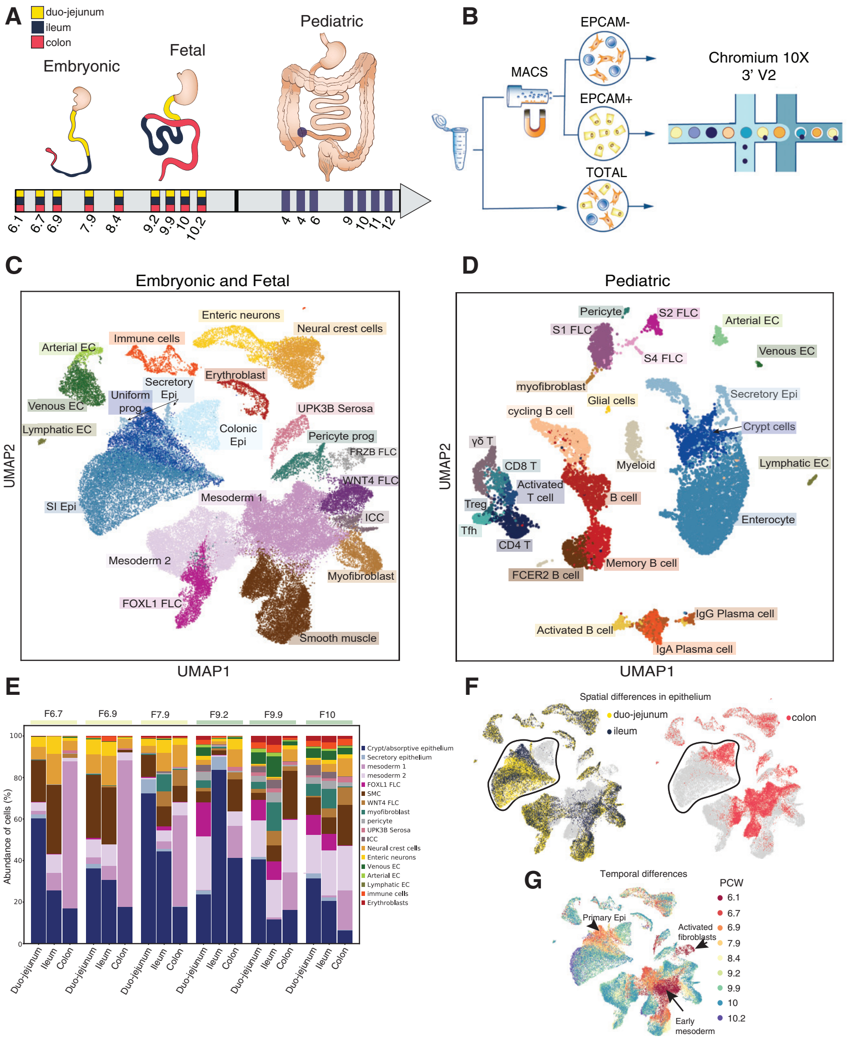

Figure 1. Single-Cell Profiling of Embryonic, Fetal and Pediatric Gut

(A) Schematic illustration of experimental design. Blue circles mark biopsy location (i.e., terminal ileum).

(B) Tissue dissociation and single-cell sequencing strategy.

(C and D) (C) and (D) UMAP projection of embryonic/fetal (n = 9 donors) and childhood/adolescence (n = 8 donors) scRNA-seq samples, respectively.

(E) Changes in embryonic/fetal cell type abundance (% of cells) at different developmental time points grouped by intestinal regions. Time point annotation colored in yellow and green are embryonic and fetal, respectively. Crypt/absorptive epithelium are SI, colonic epithelium, and uniform progenitors grouped together.

(F and G) UMAP plots colored by (F) gut region of the embryonic cells, (G) post-conception week of embryonic and fetal cells as in (C). Circled populations in F are epithelial cells. EC, endothelial cell; FLC, fibroblasts; Epi, epithelium; SI, small intestinal; prog, progenitors; ICC, interstitial cells of Cajal. See also Figure S1; Table S1.

stem cells (Fordham et al., 2013). Indeed, the use of organoid models as tools to investigate early fetal intestinal development has been demonstrated previously (Kraiczy et al., 2019). Nevertheless, the cross-talk between epithelial cell subsets and other mucosal cell types, as well as cell lineage trajectories, remain unknown. Recent studies have used single-cell RNA sequencing (scRNA-seq) to interrogate intestinal regional specification and immune system development in mice and humans (Gao et al., 2018; Nowotschin et al., 2019; Czerwinski et al., 2020; Yu et al., 2020; Li et al., 2019; Schreurs et al., 2019). However, human villus formation and epithelial dynamics have not yet been explored in detail.

In this study, we performed single-cell transcriptional profiling of human embryonic and early fetal gut samples obtained from nine human embryos spanning between ages 6 and 10 PCW. Additionally, we profiled mucosal biopsies from the small bowel of healthy children aged between 4 and 12 years and a group of children newly diagnosed with Crohn's disease (CD)—a common form of IBD. In total, we generated single-cell transcriptomes of ~90,000 primary human intestinal cells, providing a rich resource and a detailed roadmap. Using these data, as well as scRNA-seq profiles of human fetal gut derived organoids, we describe embryonic and fetal epithelium composition, trace their differentiation dynamics and signaling partners, and provide links to regenerating CD epithelium.

RESULTS

Single-Cell Map of the Human Embryonic, Fetal and Pediatric Gut

Human embryos with a post-conceptional age ranging from 6 to 10 weeks were dissected to remove the intestinal tube, which was divided into proximal small bowel (duodenum and jejunum), distal small bowel (ileum), and large bowel (colon). Additionally, we obtained small bowel (i.e., terminal ileum) mucosal biopsies from healthy children aged between 4 and 12 years (Figure 1A; Table S1). Tissue samples were dissociated into single-cell suspensions and processed using the 10x Genomics Chromium workflow (STAR Methods). In a subset of samples, the intestinal epithelial cell fraction was enriched by performing magnetic bead sorting for epithelial cell adhesion molecule (EPCAM) (Figure 1B; Table S1). In total, 62,854 fetal (n = 34) and 11,302 pediatric terminal ileal (n = 8) cells passed quality control and doublet exclusion criteria (Figure S1; Table S1).

Embryonic/fetal and pediatric datasets were processed individually to identify the cell types present in the samples. Clustering and cell-type-specific marker gene expression revealed seven major cell types in embryonic/fetal samples, including immune, erythroblast, endothelial, neural crest, smooth muscle (SMC), mesenchymal, and epithelial cell populations (Figures 2A–2D). Assessment of cellular subsets and their expression markers allowed us to further subdivide cell types, as outlined in Figures 1C and 1D. All major cell types were also identified in pediatric biopsy samples with the exception of enteric

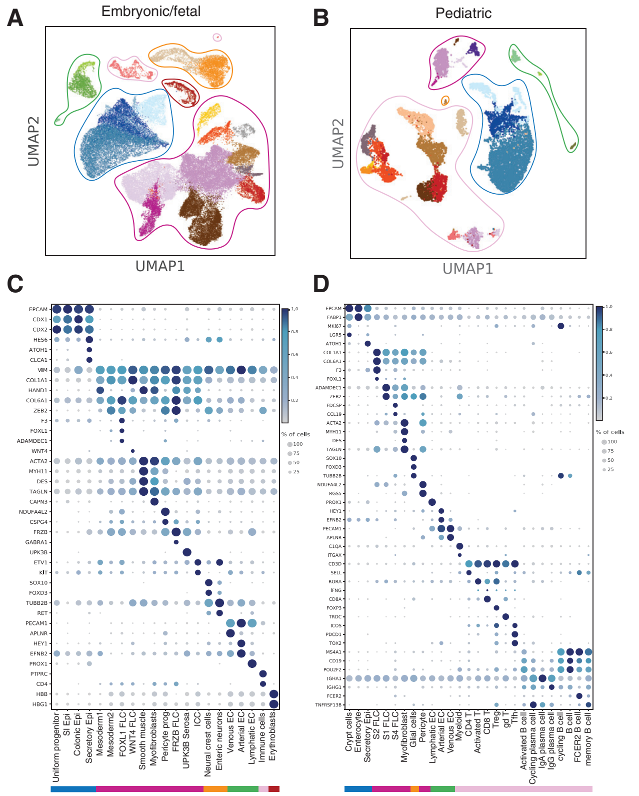

Figure 2. Cell-Type Groups and Their Marker Genes Identified in Fetal and Pediatric Datasets

(A and C) UMAP plots of fetal and pediatric datasets (A and C, respectively) broadly grouped into seven groups: epithelial (blue), mesenchymal (dark pink), neural (orange), endothelial (green), immune (light pink), and erythroid lineage (brown).

(B and D) Dot plots of relative expression and percentage of cells expressing marker genes in fetal (B) and pediatric (D) datasets. The color bars match the cell-type group colors. Epi, epithelium; FLC, fibroblasts; EC, endothelial cells; ICC, interstitial cells of Cajal.

neurons, smooth muscle, and serosal cells (Figure 1D), whose exclusion was expected, given that the depth of forceps biopsy is restricted to mucosa.

Comparing the cellular composition across the three developmental stages (embryonic, fetal, and pediatric), we observed notable differences. For example, the mesenchymal cell compartment was greatly expanded in proportion as well as diversity in embryonic and fetal samples (Figures 1C and 1D). Conversely, pediatric samples were dominated by immune cells, including follicular/memory B cells, T cells, and plasma and myeloid cells. Embryonic and fetal samples contained a comparatively smaller proportion of immune cells, including macrophages, dendritic cells, monocytes, as well as T and B cells (data not shown).

Differentiated cell states were more frequently observed in the proximal than distal regions of the developing gut (Figure 1E). For example, we observed the highest proportion of secretory epithelial subset as well as cells of immune, erythrocyte, and endothelial lineages captured in the duo-jejunum, followed by expansion in the ileum, while the smallest proportion was observed in the colon at any given time point. Conversely, the undifferentiated mesodermal cells (mesoderm 1) were most abundant in the early colonic samples and decreased by 10 PCW, while the same subset was observed only in low abundance in the duo-jejunum samples even at the earliest time points (Figure 1E).

As shown in Figure 1F, the spatiotemporal distribution of individual cell clusters demonstrated a significant separation of embryonic/fetal epithelial cell clusters according to gut region and developmental time point (Figures 1F, 1G, and S1I). Temporal separation in epithelial cells of healthy pediatric samples was less pronounced (Figures S1C and S1E). These differences highlight major developmental changes in the intestinal epithelium during the captured time periods. Hence, we next aimed to further elucidate underlying mechanisms and pathways.

Intestinal Epithelial Cell-Type Changes during Human Villus Formation

Approximately midway through the first trimester, the human intestine is lined by a thick, pseudostratified epithelium that largely fills the intestinal lumen (Figure 3A). Only 3–4 weeks later a single-cell layer starts to appear and by 10 PCW a primitive villus structure can be observed (Figure 3A). In order to examine transcriptional changes that occur during this transition, we subclustered fetal small bowel (duo-jejunum and ileum) epithelial cells based on the expression of *EPCAM*. Following dimensionality reduction, we identified 11 epithelial cell clusters and their differentially expressed genes (Figures 3B and 2E). Among them were two clusters: a cluster with high expression of canonical adult-stem-cell genes, including leucine-rich repeat-containing G-protein coupled receptor 5 (*LGR5*), achaete-scute complex homolog 2 (*ASCL2*), ephrin type-B receptor 2 (*EPHB2*), and repulsive guidance molecule BMP co-receptor B (*RGMB*) (Figures 3B and 2E), and a cluster that was defined by high expression of sonic hedgehog (*SHH*), phospholipase A2 group IIA (*PLA2G2A*), brain expressed X-linked 5 (*BEX5*), and cadherin-2 (*CDH2*) (Figures 3C and 2E). The latter cluster, which we refer to as "uniform progenitors," displayed relatively low or absent expression of *LGR5*, *ASCL2*, and *SMOC2* (Figures 3E and S2B). Conversely, the LGR5-high stem-cell cluster showed downregulation of genes expressed in the uniform progenitors, such as *BEX5* and *CDH2* (Figure S2C).

The number of uniform progenitors decreased at 10 PCW (Figure 3C), coinciding with villus emergence and the appearance of LGR5+ stem cells, immature and maturing enterocytes, and goblet and enteroendocrine cells (Figure 3C). Paneth cells were not evident and a population of *BEST4*/*OTOP2*+ enterocytes was present in the first trimester of development, confirming previous reports (Mallow et al., 1996; Parikh et al., 2019; Smillie et al., 2019). In addition, we used expression data to define the position of differentiating epithelial cell subsets along the crypt-villus (base-top) axis using scoring of gene sets previously reported in adults (Moor et al., 2018; Parikh et al., 2019) (STAR Methods). While immature and differentiated enterocytes as well as *BEST4*-enterocytes localized to the top of the axis, the cycling cells, *LGR5*+ stem cells, and uniform progenitors localized to the crypt bottom (Figure 3D).

In both chicken and mouse, the process of villus emergence affects epithelial proliferation (Walton et al., 2016a, 2016b).

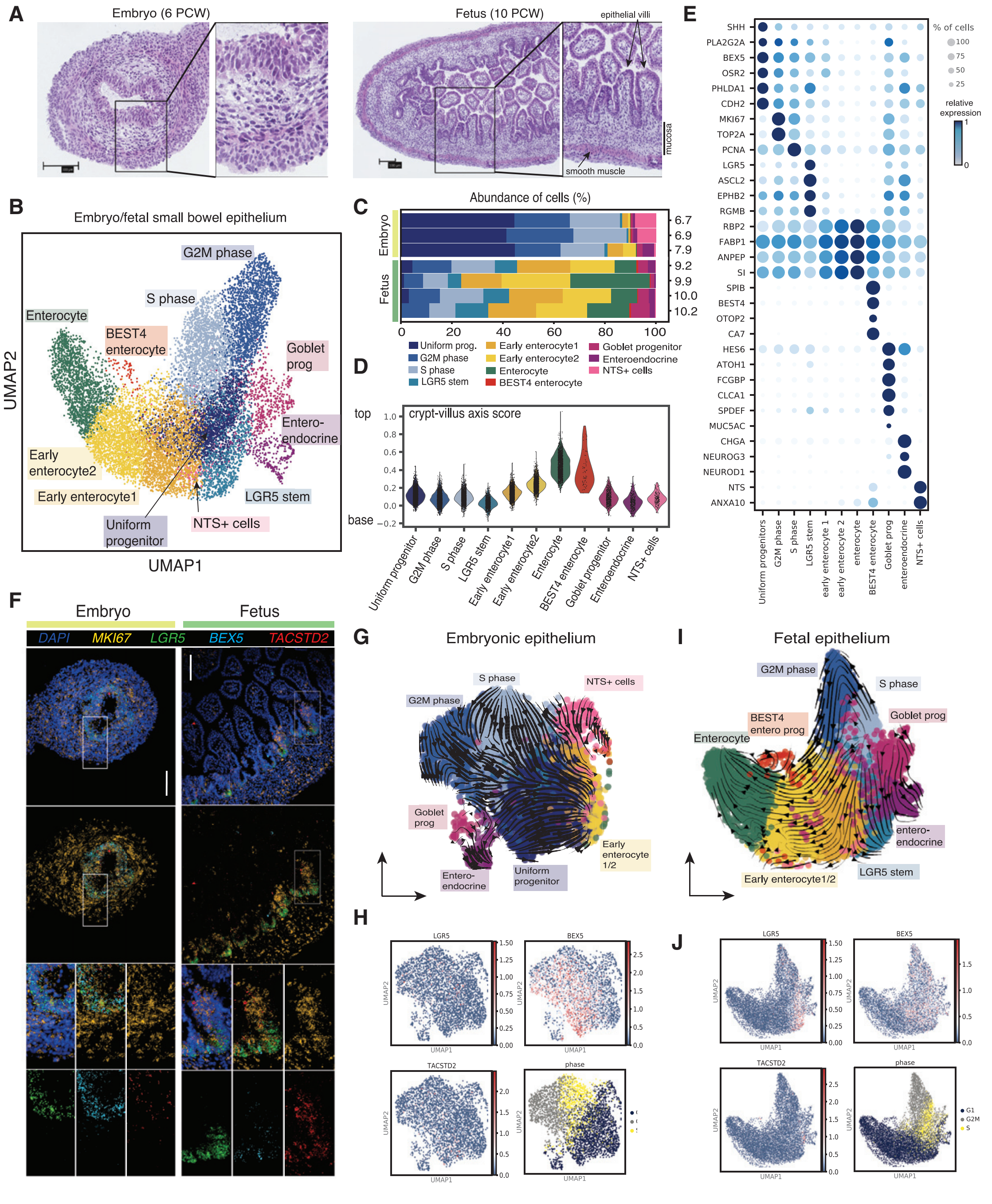

Figure 3. Epithelial Cell Composition during Villus Formation in Humans

(A) Representative hematoxylin and eosin staining of embryonic and fetal ileum at 6 and 10 PCW (n = 3 donors).

(B) Sub-clustered epithelial cells from duo-jejunum and ileum colored by cell type.

(C) Changes in epithelial cell-type abundance (% of cells) at different developmental time points and in two small bowel regions. Colors match the cell-type annotation in (B).

(D) Pseudo-spatial distribution of developing epithelial cells along the crypt-villus (base-top) axis. The axis score was derived by using the expression of selected crypt-villus axis markers as defined by Moor et al. (2018) and Parikh et al. (2019).

(E) Dotplot with marker genes used to annotate fetal epithelial cell subtypes.

(F) smFISH analysis of *MKI67*, *LGR5*, *BEX5*, and *TACSTD2* transcripts in embryonic (pseudostratified) and fetal (vilified) epithelium. (Embryonic: 6 PCW; fetal: 10 PCW.) Zoom-in boxes show channels with and without DAPI, as well as each channel independently. Scale bar: main panel, 100 μm; zoom panel, 50 μm.

(G–J) (G) Embryonic and (I) fetal epithelium scVelo graphs with overlaid arrows. Expression of *LGR5*, *BEX5*, *TACSTD2*, and cell cycle phase overlaid on (H) embryonic and (J) fetal epithelial cells shown as feature plots. See also Figure S2.

While epithelial cells located at the inter-villus region remain proliferative, those at the tip of the villus withdraw from the cell cycle. This is considered a critical event in the development of intestinal adult stem cells. We used *BEX5* as a marker of uniform progenitors alongside *LGR5* and *MKI67* to visualize epithelial changes during villus emergence. In addition, we included *TACSTD2* (*Trop2A* in mice), which has been shown to mark the murine fetal intestinal progenitors (Mustata et al., 2013). Our imaging confirmed that a similar proliferation restriction occurs in the developing human intestine, whereby embryonic epithelium is uniformly cycling, whereas at 10 PCW cycling cells become restricted to the inter-villus domains. We also noted that at the embryonic stages, uniform progenitors are *LGR5*-low and *BEX5*-high, whereas the cells restricted at the inter-villus domains upregulate *LGR5* expression and downregulate *BEX5* (Figure 3F). *TACSTD2* was expressed in both embryonic epithelium and fetal developing crypts, showing similarities to mouse models. Together, these results suggest that at the embryonic stages, the epithelium is composed of highly cycling, uniform progenitors that express low levels of LGR5.

In addition, we applied the scVelo and partition-based graph abstraction (PAGA) trajectory algorithms to epithelial cells of the small bowel (duo-jejunum and ileum) to better understand cell differentiation dynamics during the transition from embryonic (6–8 PCW) to early fetal (9–10 PCW) epithelium (Figures 3G–3J, S2D, S2E, and S2F–S2I). At the embryonic stages, rapidly cycling cells formed the start point of the trajectory and appeared to give rise to *BEX5*-high uniform progenitors. We also observed a proportion of differentiated cells, such as enteroendocrine and goblet cells, that may differentiate from cycling cells (Figures 3G–3J). Combined scVelo and PAGA trajectory analyses suggest that uniform progenitors may be capable of self-renewing as well as giving rise to differentiated cell lineages (Figures S2D, S2E, and S2F–S2I), as recently proposed in mice (Guiu et al., 2019). At the fetal stages, the differentiation dynamics change, suggesting the beginning of adult-like differentiation of LGR5+ stem cells into its progeny (Figures 3G–3J, S2E, and S2I). This finding indicates that the cycling epithelium undergoes transcriptional transition from uniform epithelium into *LGR5*+ stem cells and therefore may act as both a primitive stem cell of the early gut and as a progenitor to *LGR5*+ stem cells later in development.

In summary, *in silico* trajectory analysis revealed complex cell dynamics in the embryonic and fetal epithelium and further supports the conclusion that epithelium at embryonic stages represent a uniformly cycling epithelial progenitor cell.

Cell-Cell Cross-Talk That Supports Villus Formation in Humans

Remodeling of epithelium from pseudostratified to crypt-villus critically relies on the cross-talk with non-epithelial cell subsets. Next, we aimed to address mechanisms and signaling pathways implicated in human villus formation. First, we defined the changes in mesenchymal cell abundance across developmental time points in both small and large intestines in order to identify potential cell types that appear or become restricted during villus formation (Figure 4A). At sampled timepoints, we observed the disappearance of undifferentiated mesodermal subsets and the appearance of more differentiated mesenchymal subsets, suggesting remodeling of the mesenchymal compartment. Coinciding with epithelial remodeling, we detected the emergence of *FOXL1*+ fibroblasts in small intestinal regions. In contrast, developing SMCs were captured in both regions across embryonic and fetal time points (Figure 4A). Apart from high expression of PDGF receptor genes, *FOXL1*+ fibroblasts were marked by expression of multiple BMP ligands (*BMP3/5/7*) as well as the adult colonic mucosal S2 fibroblast marker, *F3* (Kinchen et al., 2018). We further show that *FOXL1*+ cells transcriptionally best match adult S2 cells described in the colon (Kinchen et al., 2018) (Figures S3D and S3E). In addition, we visualized *PDGFRA*-high and *FOXL1/F3* expressing mesenchymal cell clustering around the thick, pseudostratified epithelium in 6 PCW human embryos (Figures 4E and S3B) and near-forming villi at 10 PCW (Figure 4E). This observation is reminiscent of mesenchymal clustering seen in E14.5 mouse embryos (Walton et al., 2016a, 2016b).

HH pathway activation is instrumental in regulating many aspects of intestinal development. For example, mesenchymal cluster size and subsequent villus emergence have recently been shown to be controlled by GLI2-driven activation of cell polarity pathway (Walton et al., 2012; Rao-Bhatia et al., 2020). In addition, GLI2 also regulates Wnt ligand expression in *FOXL1*+ telocytes, both in stomach and intestine (Kim et al., 2020). Using human scRNA-seq data, we further address which mesenchymal subsets show activation of HH signaling based on the co-expression of HH signaling pathway components (STAR Methods). The analysis identified mesodermal cells, *FOXL1*+ fibroblasts and SMCs as the main responders to HH signaling (Figure 4B). We further validated the expression of the Sonic hedgehog gene (*SHH*) by embryonic epithelium and *in situ* expression of its receptor, *PTCH1* (Figure 4F). We show that *PTCH1* expression forms ripples with high expression by cells located around the epithelium (at the site of clustering *FOXL1*+ fibroblasts) and SMCs that were marked by *PLA2G2A* expression in scRNA-seq data (Figures 4C and S3A) as well as *in situ* (Figure S3C, white arrows).

While *FOXL1*+ fibroblasts highly expressed PDGF receptor and BMP ligand genes (Figure 4C), developing SMCs and myofibroblasts were marked by expression of BMP antagonists (*NOG*, *GREM1/2*) (Figure 4C). Similar opposing gradients were reported in the microenvironment of the adult mouse gut (McCarthy et al., 2020). We also observed expression of multiple WNT and RSPO ligand genes primarily in mesothelial serosa cells (Figure 4C), and further visualized the expression of *WNT2B* transcripts in these cells *in situ* (Figure S3B). We summarize these findings in the schematic (Figure 3D).

Using CellPhoneDB (Vento-Tormo et al., 2018; Efremova et al., 2020), we found that the mesenchymal populations displaying the highest number of cell-type-specific interactions with epithelial cell types were *FOXL1*+ and *WNT4*+ fibroblasts in both fetal and embryonic time points as well as mesothelial serosal cells in embryonic timepoints (Figures S3F and S3G). The most specific interactions between *FOXL1*+ fibroblasts and the uniform progenitors were via BMP, PDGF, Notch, Wnt, and FGF signaling pathways (Figure 4G). Other ligands secreted by *FOXL1*+ fibroblast included *NRG1*, *CXCL12* and *VEGFA*. We also identified growth factors, such as *IGF2*, *PTN*, and *MDK*, as secreted by

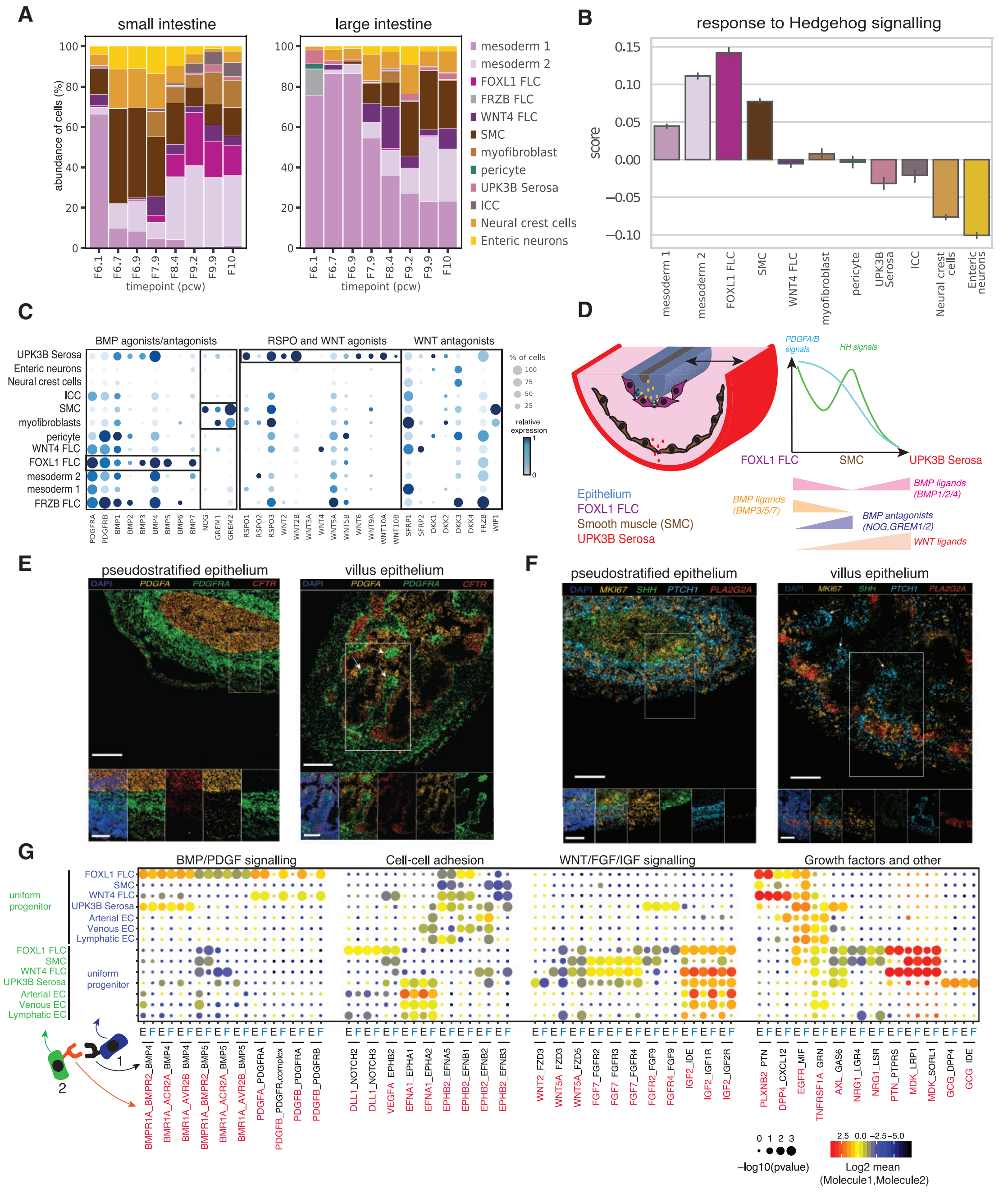

Figure 4. Cell-Cell Interactions that Support Transition from Embryonic to Fetal Epithelium in Humans

(A) Abundance of mesenchymal and neuronal cell subsets (% of cells) in developing gut from small (left panel) and large intestines (right panel).

(B) Average expression score of hedgehog (HH) pathway genes.

(C) Dot plot with expression of *BMP* and *WNT* agonists/antagonist and *RSPO* genes in all mesenchymal cells.

(D) Pseudo-positioning schematic of *PDGF* and *HH* receptor expression as well as *BMP* and *WNT* ligand expression in the cross-section of the developing small bowel upon villus formation.

(E and F) (E) Visualization of *PDGF* ligand and receptor and (F) HH pathway genes in the embryonic small intestine using smFISH at two developmental time points (left panel: embryonic; right panel: fetal). Scale bar: main panel, 100 μm. *PLA2G2A* expression marks developing smooth muscle, UPK3B+ serosal cells, and uniform progenitor cells.

(G) Dot plot of ligand-receptor interactions between uniform progenitor cells and mesenchymal/endothelial populations as predicted using CellPhoneDB analysis in embryonic (columns marked with E) and fetal (columns marked with F) samples. Point size indicates permutation p value and color indicates the scaled mean expression level of ligand and receptor. The interacting cell type and molecule pair relationship is explained in a schematic, where molecule 1 (black) in cell-type cluster 1 (blue) interacts with molecule 2 (red) in cell-type cluster 2 (yellow). FLC, fibroblasts; SMC, smooth muscle cells; EC, endothelial cells; ICC, interstitial cells of Cajal; PCW, post-conception weeks. See also Figure S3.

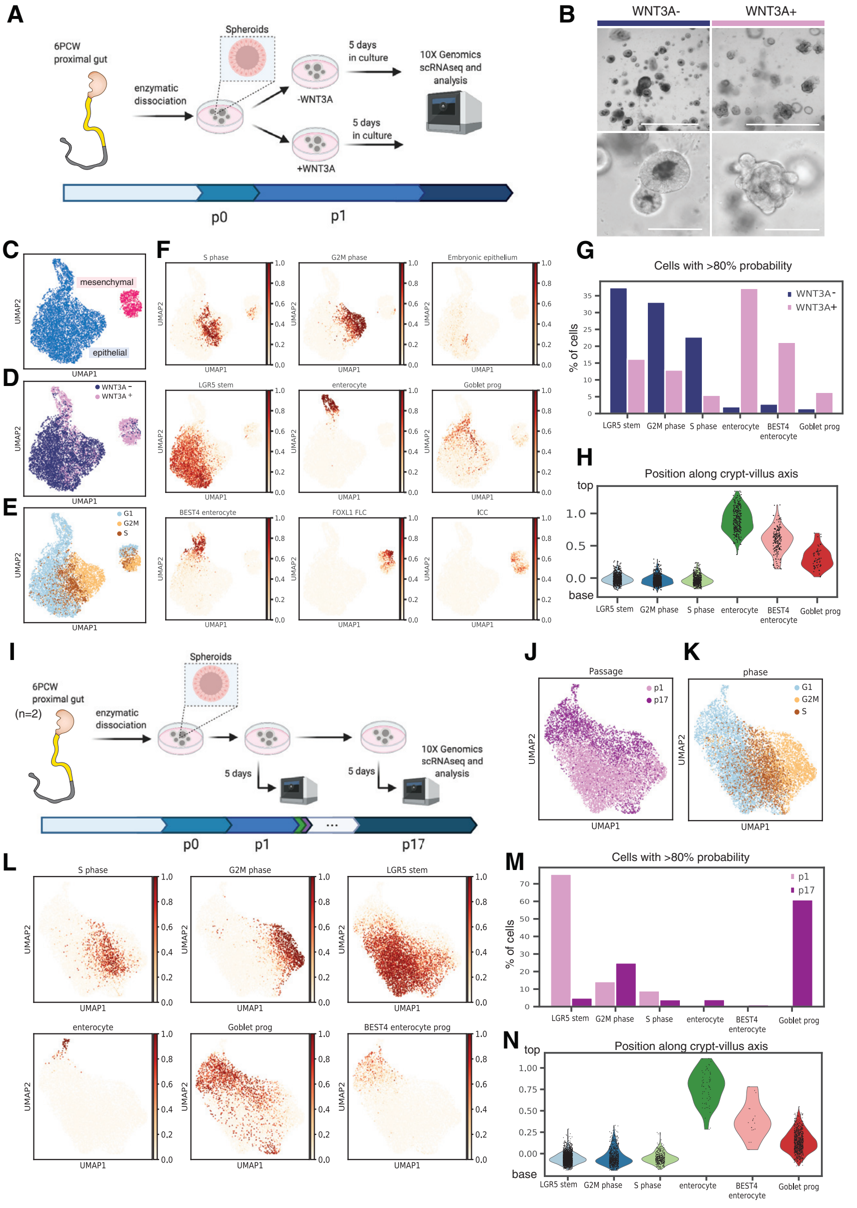

Figure 5. Fetal Intestinal Organoids Mature in Culture

(A) Schematic representation of WNT3A organoid culture experiment.

(B) Brightfield images of fetal organoids grown without (WNT3A−) or with (WNT3A+) conditional medium at passage 2, day 5 post-passage. Scale bar: top panels, 1,000 µm, top panels, 200 µm. UMAP plots of single cells from fetal organoids grown with or without WNT3A.

(C–E) Cells are colored by either (C) cell type, (D) condition, or (E) cell cycle phase.

(F) Cell-type prediction in WNT3A± organoid culture using logistic regression classifiers trained on all primary small intestinal fetal cells. UMAP plots show overlaid predicted probability for selected cell types. Abundance of cell types (% of cells) in organoids, as confidently predicted (over 80% probability of a single cell type) by the logistic regression classifier.

(H) Pseudo-spatial distribution of organoid epithelial cells along the crypt-villus (base-top) axis.

(I) Schematic representation of experimental design. The experiment was performed using two independent biological samples (replicate 1 from 5.4 PCW and replicate 2 from 6.4 PCW).

(J and K) On day 5 of passage 1 (p1) organoids were split and one fraction was kept in the culture while the other was dissociated and processed using 3′ V2 10× protocol. On day 5 of passage 17 (p17) the organoids were dissociated and processed using the 10× platform again. UMAP plots with single cells colored by either (J) passage or (K) cell cycle phase.

(L) Prediction of cell types of p1 and p17 organoids using logistic regression trained on primary fetal cells. UMAP plots represent visualization with overlaid predicted probability for selected cell types as in (F). (M) Predicted cell-type abundance or PCW age of fetal organoids grown for 1 or 17 passages as predicted using logistic classifier. N) Pseudo-spatial distribution of organoid epithelial cells along the crypt-villus (base-top) axis. FLC, fibroblasts; SMC, smooth muscle cells; EC, endothelial cells; ICC, interstitial cells of Cajal; PCW, post-conception weeks; prog, progenitor. Organoids were derived from fetal tissues BRC2038- 6.4 PCW, BRC2039 −5.4 PCW, BRC2206- 6.5 PCW. See also Figure S4.

this mesenchymal subset and received by uniform progenitors (Figure 4G).

Together, these data demonstrate interactions relevant to the human villus formation and point toward signaling pathways implicated in early human gut development.

Fetal Organoids Show *In Vitro* Maturation Recapitulating *In Vivo* Epithelial Transition

The ability of intestinal epithelial stem cells to give rise to all cell subsets has led to the development of intestinal organoid culture models (Sato et al., 2011a, 2011b; Tsai et al., 2017). Such organoids can be generated from the human fetal gut, providing the opportunity to investigate epithelial cell-intrinsic and -extrinsic developmental mechanisms (Fordham et al., 2013; Kraiczy et al., 2019). Here, we applied scRNA-seq to developing human gut organoids (STAR Methods, Table S1) and performed *in silico* analyses by classifying epithelial cells using transcriptional profiles derived from primary tissue.

In the adult small bowel mucosa, Paneth cells have been found to express the Wnt-agonist WNT3A, thereby providing a critical signal to the stem-cell niche (Sato et al., 2011a, 2011b). In addition to the absence of Paneth cells in the developing gut, we were also unable to identify any *WNT3A*-expressing mesenchymal cells in our scRNA-seq datasets (Figure 4C). Culture of intestinal epithelial organoids (IEOs) in the presence of recombinant WNT2B protein, expressed by the embryonic/fetal mesenchymal subsets (Figure 4C), did not lead to morphological difference or transcriptional activation of the Wnt signaling pathway (Figures S4D–S4H). However, given that WNT3A forms a key ingredient of previously reported human adult and fetal mucosa-derived intestinal epithelial organoid cultures (Sato et al., 2011a, 2011b; Fordham et al., 2013; Kraiczy et al., 2019) we aimed at assessing its requirement

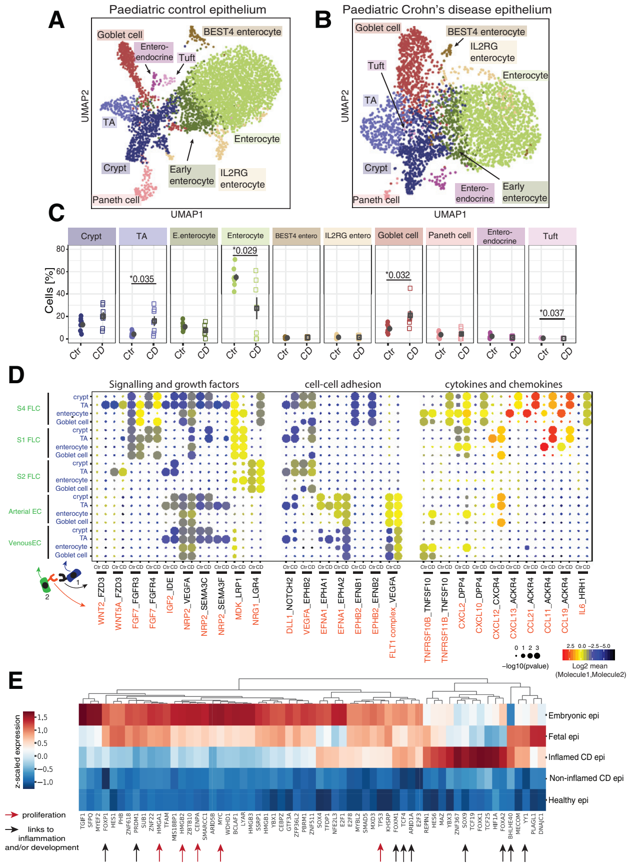

Figure 6. Epithelial Cell Dynamics in Crohn's Disease Patients Show Transcriptional Similarities to Developing Epithelium

(A and B) (A) and (B) UMAP plots of epithelial cell subtypes in healthy children (n = 8) and patients with CD (n = 7), respectively.

(C) Epithelial cell-type changes in pediatric health and CD patients. TA, enterocytes, goblet cells, and tuft cell proportions were changed significantly between control and CD patients (p values indicated, t test).

(D) Dot plot with ligand-receptor interactions between stromal (S1-S4 FLC) and endothelial cells (Arterial/venous EC) and selected epithelial cell types. Point size indicates permutation p value (CellPhoneDB). Color indicates the scaled mean expression level of ligand and receptor. FLC, fibroblasts; EC, endothelial cell.

(E) Heatmap showing the relative mean expression of transcription factors, which were identified to be differentially expressed in CD epithelium, across epithelium from five groups. "Non-inflamed CD" was a group of patients with minimal epithelial composition changes as in (Figure S5F, arrows). Arrows point to genes discussed in text that either have previously been linked to proliferation (red arrows) or inflammation and/or development (black arrows). Epi, epithelium; CD, Crohn's disease. See also Figures S5 and S6.

and impact on fetal gut organoids. IEO cultures were generated from the proximal small bowel and cultured in the presence or absence of WNT3A-conditioned medium (Figure 5A). Inclusion of WNT3A led to the presence of budding, crypt structures, while organoids lacking WNT3A appeared more spheroid-like (Figure 5B). Interestingly, single-cell transcriptional profiling of these cultures at an early passage (i.e., passage 2—approximately 2–3 weeks in culture) revealed the presence of intestinal epithelial cells as well as a small fraction of mesenchymal cells that resembled *FOXL1*-fibroblasts (Figures 5F, S4A, and S4B). Organoids were viable for several weeks even if cultured in the absence of WNT3A and showed evidence of active cell cycling (Figure 5E). However, WNT3A was required for long-term culture as WNT3A− organoids showed reduced viability and could not be cultured beyond 6 weeks. Importantly, observed phenotypic differences were matched by dramatic transcriptional changes leading to a clear separation of cells according to culture conditions (Figure 5D). Removing or reducing WNT3A in adult mucosa-derived IEOs has been shown to induce differentiation of epithelial cells and a reduced expression of LGR5 (Kraiczy et al., 2019). In contrast, when assessing epithelial cell identity and composition of fetal organoids using a logistic regression model trained on the primary fetal scRNA-seq profiles, organoids cultured in the presence of WNT3A were found to contain a greater proportion of differentiated cell types, including enterocytes and enteroendocrine cells compared with those cultured in its absence (Figures 5F and 5G).

Previous work suggests that human fetal gut organoids undergo a degree of *in vitro* maturation in culture (Tsai et al., 2017; Kraiczy et al., 2019). In order to examine this further, we generated organoids from embryonic gut samples aged 6 PCW and kept them in complete culture medium (containing WNT3A) over 5 months (Figure 5I). Single-cell profiling was applied to cultures once they were first established (after one week, passage 1) and following 5 months in culture (17 passages). Uniform manifold approximation and projection (UMAP) clustering revealed separation according to duration in culture, suggesting that significant transcriptional changes occur over time (Figure 5J). Major differences were also observed with regard to the predicted cell composition, such that older cultures contained a higher proportion of differentiated cell types, including enterocytes and goblet cell progenitors (Figures 5L, 5M, and S4C). Similar to the primary fetal scRNA-seq, organoid-derived enterocytes and BEST4-enterocytes were predicted to localize to the top of the villus axis, while the cycling cells—LGR5-stem cells, to the bottom of this axis (Figures 5H and 5N)—providing further evidence of epithelial cell maturity.

In summary, our findings reveal effects of Wnt signaling and specifically WNT3A on human fetal epithelial organoid cell diversity and maturity.

Parallels between Fetal and Inflamed Epithelium in Crohn's Disease Patients

Alterations in the composition, function, and cell dynamics of the intestinal epithelium are thought to play a critical role in the pathogenesis of CD. Moreover, a link has been proposed between early fetal development and regenerating epithelium in mice by demonstrating partial reprogramming of the regenerating colonic epithelium (Yui et al., 2018; Wang et al., 2019). In order to investigate these observations in humans, we performed scRNA-seq terminal ileum biopsies obtained from children newly diagnosed with CD (n = 7).

Compared with non-IBD samples, CD patients showed increased vascularization marked by expansion of arterial and venous endothelial cells as well as increased numbers of fibroblasts resembling S4 stromal cells (Figure S5C) first described in adult ulcerative colitis patients (Kinchen et al., 2018). We directly compared their transcriptional profiles using marker genes and a logistic regression model that showed high transcriptional similarity between colonic and ileal stromal populations (Figures S6A–S6F). In the immune compartment, we observed expansion of

myeloid cells, CD4 T cells, and IgG plasma cells (Figure S5D), features recently described in ileal CD patients (Martin et al., 2019).

Comparing epithelial cell composition between CD and age-matched control samples, we observed significant differences, including an increase in transit amplifying (TA), goblet, and tuft cells, while the proportion of fully differentiated enterocytes was significantly reduced in CD epithelium (Figures 6A–6C and S5F). We then interrogated the cross-talk between stromal cells and the affected intestinal epithelial subtypes in the context of CD. We identified a number of cell-cell interactions that were specific between CD cell-type pairs (Figure 6D). For example, S4 fibroblasts were found to uniquely signal to CD TA cells via a *WNT2* ligand that were received by epithelial subtypes via *FZD3* receptor (Figure 6D). In addition, we observed chemokines and cytokines, such as *CXCL2*, *CXCL10*, *CXCL13*, *CCL11*, *and IL6* that were expressed by the S4 fibroblast population and received by intestinal epithelial cells. Interestingly, among cytokine interactions we found *TNFSF10-TNFRSF10B* signals that promote tumor necrosis factor (TNF)-related apoptosis and elimination of intestinal epithelial cells (Begue et al., 2006; Wu et al., 2019). Compared to healthy patients, specificity of *TNFSF10-TNFRSF10B* signaling in CD patients was reduced between goblet cells and S4 fibroblasts, while it remained similar in enterocytes. These changes may contribute to the selective loss of enterocytes and increase in goblet cell abundance seen in CD. Together, these findings highlight the complex cross-talk between the epithelium and surrounding stromal cells, which is likely to contribute toward chronic mucosal inflammation observed in childhood-onset CD.

Finally, growth factor interactions that we observed during development, such as interactions via *WNT5A*, *FGF7*, *IGF2*, *MDK*, and *NRG1* ligands, were also changed between controls and CD patients. To further understand the regulation of cell-cell interactions, we aimed to identify the transcription factors that were shared between CD and developing epithelium (STAR Methods). As shown in Figure 6E, we identified a number of such genes, some of which have been linked to IBD pathogenesis. Examples include the B lymphocyte-induced maturation protein-1 (Blimp1; encoded by the gene *PRDM1*) (Harper et al., 2011; Muncan et al., 2011; Ellinghaus et al., 2013); Forkhead Box transcription factors, *FOXP1* and *FOXM1*, linked to CD (de Lange et al., 2017; Bo et al., 2018); tumor suppressor *ARID1A*, recently reported to be under positive selection for somatic mutations in IBD colon (Olafsson et al., 2020); cell proliferation genes, such as *TP53* and *MYC*, associated with inflammation induced colorectal cancers (Horvath et al., 2015; Du et al., 2017; Lu et al., 2017); and *HMGA1*/*HMGB2* genes involved in the stem-cell expansion and associated with IBD (Vitali et al., 2011; Takaishi et al., 2012; Bush et al., 2013; Xian et al., 2017). Furthermore, our analyses identified several transcription factors implicated in stem cell and embryonic developmental. For example, the Wnt signaling transcription factor, TCF4 (Barker et al., 1999; Wehkamp et al., 2007), as well as *FOXA2*, and *SOX9*, expression of which is associated with development.

Taken together, our results confirm previous reports of altered intestinal epithelial cell dynamics in regenerating CD epithelium and identify several disease-associated cell-cell interactions in childhood-onset CD. Importantly, we provide supportive evidence for the partial re-activation of developmental transcriptional pathways in CD epithelium.

DISCUSSION

Previous studies have reported the presence of proliferative, immature progenitor epithelial cells in the human fetal intestine at around 10 PCW (Fordham et al., 2013; Guiu et al., 2019). We demonstrate that this cell population forms the vast majority of pseudostratified intestinal epithelium in the human embryo (6–8 PCW) and expresses *CDH2*, *BEX5*, *SSH*, and *PLA2G2A*, all of which have been previously linked to the stem-cell potential. For example, *CDH2* was linked to regulation of cell fate decision in the mesodermal lineage (Alimperti and Andreadis, 2015), and *SHH* to the initiation of villus formation in the developing mouse intestine (Shyer et al., 2015), while BEX-family genes were found to be expressed in tissue stem/progenitor cells (Ito et al., 2014). In addition, we demonstrate *LGR5* expression in the embryonic epithelium, albeit at lower levels than in fetal tissue. At the embryonic stages, all epithelium is uniformly cycling, while at the fetal stages, cycling cells become restricted to the bottom of the inter-villus domains, and upregulate *LGR5* expression. While analogous processes of early epithelial development were previously reported in chicken and mouse (Shyer et al., 2015), scRNA-seq data provide insights into the diversity and maturity of epithelial cell types found at fetal stages. Nevertheless, lineage tracing experiments in organoid cultures are necessary to provide evidence on the source of LGR5+ stem cells in humans.

The presence of secretory cells, including enteroendocrine and goblet cells, in multiple embryonic samples prompted us to hypothesize that the more abundant, cycling, uniform progenitors, as opposed to a few captured LGR5 stem cells, may be the source of secretory cells. These observations are in keeping with recent lineage tracing experiments by Guiu et al. (2019) that suggest the presence of alternative sources for differentiated cell subsets during development, given that fetal LGR5 cells alone are unable to sustain intestinal growth. *In silico* trajectory analysis imply that the uniform embryonic progenitors and cycling cells may be the source of differentiated cell subsets captured in embryonic stages; however, future studies are needed to address the source of differentiated cell subsets in the fetal gut.

Intestinal villus formation is one of the key developmental milestones of the first trimester. While common pathways have been proposed to mediate villus formation in chicken and mouse (*Bmp*, *Hh*, *Pdgf*), the mechanisms differ between species. In the chicken model, force generated by smooth muscle progressively deforms the epithelium to generate mucosal folds. In turn, the mesenchymal clusters form at the villus tips and restrict epithelial proliferation via Bmp signals (Shyer et al., 2015). A different mechanism was proposed in mice, where villus formation is driven by self-organizing mesenchymal cell clusters (Karlsson et al., 2000; Walton et al., 2012, 2016a, 2016b) and is uncoupled from the development of smooth muscle layers. Recent reports implicate the mesenchymal Fat4/PCP pathway in organization of mesenchymal clusters and demarcation of the emerging villus (Rao-Bhatia et al., 2020).

Using scRNA-seq data, we show that the appearance of FOXL1 fibroblasts was coincidental with epithelial changes in human intestinal epithelium. Foxl1 in mice marks a population of subepithelial telocytes that are essential for the intestinal stem-cell niche (Shoshkes-Carmel et al., 2018; Kaestner, 2019; McCarthy et al., 2020). In adult mice, telocytes support epithelial

zonation along the villus and were recently shown to express Lgr5, Bmp, and Wnt ligands at the villus tip (Bahar Halpern et al., 2020). Our scRNA-seq and imaging data suggest that *FOXL1*+ fibroblasts start developing at the embryonic stage in the absence of any visible villi. *FOXL1*+ fibroblasts were also the main responders to HH signaling and expressed BMP and PDGFR genes during development, suggestive of their role as clustering mesenchymal cells critical for villus formation. Using *in silico* ligand-receptor analyses, we further identified multiple ligands-encoding genes, including *WNT5A*, *WNT2*, *NRG1*, and *IGF2*, with potential to modulate early villus formation in humans. Finally, we show transcriptional similarities between *FOXL1*+ fibroblasts and *S2*+ fibroblasts found in the human adult gut, suggesting that two cell states may represent the same lineage of mesenchymal cells equivalent to murine telocytes.

The mesenchymal cell clustering and subsequent villus emergence were proposed to follow a proximal-to-distal wave (Spence et al., 2011; Walton et al., 2012). Fordham et al. (2013) challenged this view by culturing fetal epithelial cells from proximal, middle, and distal regions of the mouse gut and observing the opposite trend of differentiation in the organoid system. While in this work restricted cell lineages were traced (Pdgfra+ mesenchymal cell clusters or differentiating epithelial cells), scRNA-seq allows for the investigation of multiple cell lineages at the same time. Our dataset provides evidence that multiple cell lineages differentiate and, in case of immune cells, home to the proximal intestinal regions first. We also observed differences in vascularization between three regions, where endothelial cells differentiated in the proximal-to-distal wave during human intestinal development.

IEOs have been generated from human fetal gut and shown to undergo a degree of *in vitro* development highlighting their use as powerful experimental tools (Fordham et al., 2013; Guiu et al., 2019; Kraiczy et al., 2019). Here, we combined generation of fetal organoids with single-cell profiling to interrogate fetal culture composition. Our findings indicate that, while not required for their establishment and short-term culture, WNT3A is essential for long-term propagation of fetal organoids and was found to be associated with a higher proportion of differentiated cell subsets. This parallels studies in mice, where embryonic progenitors were able to proliferate independent of Wnt prior to villus formation but not after (Chin et al., 2016). These findings also point to differences between adult and fetal gut epithelium as the generation of adult mucosa-derived intestinal organoids critically relies on the presence of WNT3A, while its withdrawal leads to increased differentiation into epithelial cell subsets and reduced expression of *LGR5* (Fordham et al., 2013; Kraiczy et al., 2019). Furthermore, organoids kept in culture for several months were found to contain an increased proportion of differentiated cell subsets as well as an increased number of LGR5+ cells. This suggests that current intestinal culture conditions select for highly proliferating cells. Finally, our *in vitro* studies further illustrate the utility of single-cell transcriptomics as a critical reference for validating and interpreting fetal organoid culture work.

A developmental origin of disease pathogenesis has been proposed for many complex, multifactorial conditions. IBD, and particularly CD, are thought to be caused by a complex interplay between the environment and genetic predisposition leading to an irreversibly altered immune response. Recent studies have reported expansion of a colonic mesenchymal subset in adult ulcerative colitis and associated this with resistance to anti-TNF treatment (Kinchen et al., 2018; Smillie et al., 2019). We observed expansion of a similar mesenchymal population in childhood CD, suggesting similarities between adult- and pediatric-onset IBD. In addition, comparison between CD and healthy epithelium suggests that CD epithelium is rapidly cycling and poised for goblet cell differentiation, consistent with previous reports (Gersemann et al., 2009). Furthermore, we describe ligand and receptor pairs that uniquely signal between affected epithelial subsets and expanded stromal populations, including WNT2 ligands received by TA cells. This provides a possible mechanism to sustain intestinal regeneration in disease.

Previous studies in mice have linked epithelial cell properties in the inflamed gut to the physiological status observed in early fetal development (Yui et al., 2018; Guiu et al., 2019; Wang et al., 2019). Here, we provide evidence in humans that regenerating CD epithelium shares transcription factor programs otherwise present only in fetal epithelium. Identified transcription factors including *TP53*, *MYC*, *HMGA1*, and *HMGA2* point to increased epithelial proliferation, which we also observed as an increase in TA cell abundance. Other genes have been reported in development of epithelium or inflammatory cells in other organs. Among them is the zinc-finger transcription factor, Yin and Yang (*YY1*), which has been shown to play a critical role in lung epithelial cell development and TGF-beta-induced lung fibrosis (Boucherat et al., 2015; Zhang et al., 2019). Another example is the expression of basic helix–loop–helix 40 (*BHLH40*), expression of which has been observed in a wide range of cells and tissues, including T cells, macrophages, dendritic cells, and the gastric epithelium (Lin et al., 2014; Teng et al., 2020). *BHLH40* was found to control cytokine production by T cells, thereby playing a critical role in the development of autoimmune neuroinflammation (Lin et al., 2014; Yu et al., 2018). Finally, our analyses confirm previous reports of Forkhead BoxM1 (*FOXM1*, also HFH-11) transcription factor being expressed in embryonic epithelial cell with its expression becoming reactivated in adult cell types by proliferative signals or oxidative stress (Ye et al., 1997).

In summary, we provide a detailed single-cell map of the human gut during embryonic, fetal, and pediatric health as well as during inflammatory disease and dissect transcriptional changes in epithelial cell dynamics during intestinal life.

STAR★METHODS

Detailed methods are provided in the online version of this paper and include the following:

- KEY RESOURCES TABLE
- RESOURCE AVAILABILITY
  - Lead Contact
  - Materials Availability
  - Data and Code Availability
- EXPERIMENTAL MODEL AND SUBJECT DETAILS
  - Fetal and Paediatric Tissue Sampling
- METHOD DETAILS
  - Fetal and Paediatric Tissue Dissociation
  - Intestinal Organoid Culture
  - Tissue Freezing, Sectioning and RNAscope
  - Single-cell RNA-sequencing

CellPress
OPEN ACCESS

- Processing FASTQ Files and Quality Control
- Doublet Removal
- Clustering, Visualisation and Cell Annotation
- SCVELO AND PAGA TRAJECTORY ANALYSIS
  - Inferring Cell-Cell Interactions
  - Cellular Composition Classification
  - Transcription Factors in Epithelium
  - Crypt-Villus Axis and HH Signalling Score
- QUANTIFICATION AND STATISTICAL ANALYSIS
  - Percentage of Cells and Statistical Analysis

SUPPLEMENTAL INFORMATION

Supplemental Information can be found online at https://doi.org/10.1016/j.devcel.2020.11.010.

ACKNOWLEDGMENTS

We acknowledge the support received from the Wellcome Sanger Cytometry Core Facility, Cellular Genetics Informatics team, Cellular Generation and Phenotyping (CGaP) core facility and Core DNA Pipelines. This work was financially supported by: a Horizon 2020 grant, (668294, "Intestinal Tissue Engineering Solution for children with short bowel syndrome," L.V.); an ERC Advanced Grant (New-Chol, L.V.); the Cambridge University Hospitals National Institute for Health Research Biomedical Research Centre (L.V.); a core support grant from the Wellcome Trust and MRC to the Wellcome Trust - Medical Research Council Cambridge Stem Cell Institute (L.V.); the Wellcome Trust (WT206194, S.A.T.); the European Research Council (646794, ThDefine, S.A.T.); an MRC New Investigator research grant (MR/T001917/1, M.Z.); and a project grant from the Great Ormond Street Hospital Children's Charity, Sparks (V4519, M.Z.). We thank Dr. Franco Torrente and Dr. Camilla Salvestrini as well as Claire Glemas for recruiting pediatric patients and obtaining biopsy samples, and Professor Roger Barker and Xiaoling He for access to fetal tissue. We thank Mirjana Efremova, Krzysztof Polański, and Jong-Eun Park for analysis support and insightful discussions, and Lira Mamanova and Liam Bolt for sequencing support. We acknowledge J. Eliasova for the graphical images. We thank the tissue donors and donor families. This publication is part of the Human Cell Atlas -www.humancellatlas.org/publications.

AUTHOR CONTRIBUTIONS

A.R., R.E., L.V., M.Z., and S.A.T. initiated, designed, and supervised the project. A.R., R.E., and K.N. performed tissue processing, organoid culture, and scRNA-seq experiments. R.E. analyzed single-cell data and generated figures. T.G. contributed to data analysis. K.R.J., D.O., and T.G. supported analyses, critical discussion, and interpretation of data. K.R., L.T., S.P., and O.A.B. performed tissue sectioning, staining, and imaging. R.H. carried out pediatric patient recruitment, obtained biopsy samples, and documented clinical data. R.E., A.R., and M.Z. wrote the manuscript. All authors contributed to discussion and interpretation of results as well as editing of the manuscript.

DECLARATION OF INTERESTS

The authors declare no competing interests.

Received: April 30, 2020
Revised: September 4, 2020
Accepted: November 6, 2020
Published: December 7, 2020

REFERENCES

Alimperti, S., and Andreadis, S.T. (2015). CDH2 and CDH11 act as regulators of stem cell fate decisions. Stem Cell Res. *14*, 270–282.

Bahar Halpern, K., Massalha, H., Zwick, R.K., Moor, A.E., Castillo-Azofeifa, D., Rozenberg, M., Farack, L., Egozi, A., Miller, D.R., Averbukh, I., et al. (2020). Lgr5+ telocytes are a signaling source at the intestinal villus tip. Nat. Commun. *11*, 1936.

Barker, N., Huls, G., Korinek, V., and Clevers, H. (1999). Restricted high level expression of Tcf-4 protein in intestinal and mammary gland epithelium. Am. J. Pathol. *154*, 29–35.

Barker, N., van Es, J.H., Kuipers, J., Kujala, P., van den Born, M., Cozijnsen, M., Haegebarth, A., Korving, J., Begthel, H., Peters, P.J., and Clevers, H. (2007). Identification of stem cells in small intestine and colon by marker gene Lgr5. Nature *449*, 1003–1007.

Bayraktar, O.A., Bartels, T., Holmqvist, S., Kleshchevnikov, V., Martirosyan, A., Polioudakis, D., Ben Haim, L., Young, A.M.H., Batiuk, M.Y., Prakash, K., et al. (2020). Astrocyte layers in the mammalian cerebral cortex revealed by a single-cell in situ transcriptomic map. Nat. Neurosci. *23*, 500–509.

Becht, E., McInnes, L., Healy, J., Dutertre, C.A., Kwok, I.W.H., Ng, L.G., Ginhoux, F., and Newell, E.W. (2018). Dimensionality reduction for visualizing single-cell data using UMAP. Nat. Biotechnol. https://doi.org/10.1038/nbt.4314.

Begue, B., Wajant, H., Bambou, J.C., Dubuquoy, L., Siegmund, D., Beaulieu, J.F., Canioni, D., Berrebi, D., Brousse, N., Desreumaux, P., et al. (2006). Implication of TNF-related apoptosis-inducing ligand in inflammatory intestinal epithelial lesions. Gastroenterology *130*, 1962–1974.

Bergen, V., Lange, M., Peidli, S., Wolf, F.A., and Theis, F.J. (2020). Generalizing RNA velocity to transient cell states through dynamical modeling. Nat. Biotechnol. https://doi.org/10.1038/s41587-020-0591-3.

Bo, L., Fu, H., and Yang, J. (2018). Comprehensive analysis of gene expression profiles provides insight into the pathogenesis of Crohn's disease. Mol. Med. Rep. *18*, 2643–2650.

Boucherat, O., Landry-Truchon, K., Bérubé-Simard, F.A., Houde, N., Beuret, L., Lezmi, G., Foulkes, W.D., Delacourt, C., Charron, J., and Jeannotte, L. (2015). Epithelial inactivation of Yy1 abrogates lung branching morphogenesis. Development *142*, 2981–2995.

Bush, B.M., Brock, A.T., Deng, J.A., Nelson, R.A., and Sumter, T.F. (2013). The Wnt/β-catenin/T-cell factor 4 pathway up-regulates high-mobility group A1 expression in colon cancer. Cell Biochem. Funct. *31*, 228–236.

Chin, A.M., Hill, D.R., Aurora, M., and Spence, J.R. (2017). Morphogenesis and maturation of the embryonic and postnatal intestine. Semin. Cell Dev. Biol. *66*, 81–93.

Chin, A.M., Tsai, Y.H., Finkbeiner, S.R., Nagy, M.S., Walker, E.M., Ethen, N.J., Williams, B.O., Battle, M.A., and Spence, J.R. (2016). A dynamic WNT/β-catenin signaling environment leads to WNT-independent and WNT-dependent proliferation of embryonic intestinal progenitor cells. Stem Cell Rep. *7*, 826–839.

Cilieborg, M.S., Boye, M., and Sangild, P.T. (2012). Bacterial colonization and gut development in preterm neonates. Early Hum. Dev. *88*, S41–S49.

Czerwinski, M., Holloway, E.M., Tsai, Y.-H., Wu, A., Yu, Q., Wu, J., Walton, K.D., Sweet, C., Childs, C., et al. (2020). In vitro and in vivo development of the human intestinal niche at single cell resolution. bioRxiv. https://doi.org/10.1101/2020.01.31.928788.

de Lange, K.M., Moutsianas, L., Lee, J.C., Lamb, C.A., Luo, Y., Kennedy, N.A., Jostins, L., Rice, D.L., Gutierrez-Achury, J., Ji, S.-G., et al. (2017). Genome-wide association study implicates immune activation of multiple integrin genes in inflammatory bowel disease. Nat. Genet. *49*, 256–261.

Du, L., Kim, J.J., Shen, J., Chen, B., and Dai, N. (2017). KRAS and TP53 mutations in inflammatory bowel disease-associated colorectal cancer: a meta-analysis. Oncotarget *8*, 22175–22186.

Dupaul-Chicoine, J., Dagenais, M., and Saleh, M. (2013). Crosstalk between the intestinal microbiota and the innate immune system in intestinal homeostasis and inflammatory bowel disease. Inflam. Bowel Dis. *19*, 2227–2237.

Efremova, M., Vento-Tormo, M., Teichmann, S.A., and Vento-Tormo, R. (2020). CellPhoneDB: inferring cell-cell communication from combined expression of multi-subunit ligand-receptor complexes. Nat. Protoc. *15*, 1484–1506.

Ellinghaus, D., Zhang, H., Zeissig, S., Lipinski, S., Till, A., Jiang, T., Stade, B., Bromberg, Y., Ellinghaus, E., Keller, A., et al. (2013). Association between

variants of PRDM1 and NDP52 and Crohn's disease, based on exome sequencing and functional studies. Gastroenterology *145*, 339–347.

Fordham, R.P., Yui, S., Hannan, N.R., Soendergaard, C., Madgwick, A., Schweiger, P.J., Nielsen, O.H., Vallier, L., Pedersen, R.A., Nakamura, T., et al. (2013). Transplantation of expanded fetal intestinal progenitors contributes to colon regeneration after injury. Cell Stem Cell *13*, 734–744.

Gao, S., Yan, L., Wang, R., Li, J., Yong, J., Zhou, X., Wei, Y., Wu, X., Wang, X., Fan, X., et al. (2018). Publisher Correction: tracing the temporal-spatial transcriptome landscapes of the human fetal digestive tract using single-cell RNA-sequencing. Nat. Cell Biol. *20*, 1227.

Gersemann, M., Becker, S., Kübler, I., Koslowski, M., Wang, G., Herrlinger, K.R., Griger, J., Fritz, P., Fellermann, K., Schwab, M., et al. (2009). Differences in goblet cell differentiation between Crohn's disease and ulcerative colitis. Differentiation *77*, 84–94.

Geske, M.J., Zhang, X., Patel, K.K., Ornitz, D.M., and Stappenbeck, T.S. (2008). FGF9 signaling regulates small intestinal elongation and mesenchymal development. Development *135*, 2959–2968.

Grosse, A.S., Pressprich, M.F., Curley, L.B., Hamilton, K.L., Margolis, B., Hildebrand, J.D., and Gumucio, D.L. (2011). Cell dynamics in fetal intestinal epithelium: implications for intestinal growth and morphogenesis. Development *138*, 4423–4432.

Guiu, J., Hannezo, E., Yui, S., Demharter, S., Ulyanchenko, S., Maimets, M., Jørgensen, A., Perlman, S., Lundvall, L., Mamsen, L.S., et al. (2019). Tracing the origin of adult intestinal stem cells. Nature *570*, 107–111.

Harper, J., Mould, A., Andrews, R.M., Bikoff, E.K., and Robertson, E.J. (2011). The transcriptional repressor Blimp1/Prdm1 regulates postnatal reprogramming of intestinal enterocytes. Proc. Natl. Acad. Sci. USA *108*, 10585–10590.

Horvath, B., Liu, G., Wu, X., Lai, K.K., Shen, B., and Liu, X. (2015). Overexpression of p53 predicts colorectal neoplasia risk in patients with inflammatory bowel disease and mucosa changes indefinite for dysplasia. Gastroenterol. Rep. *3*, 344–349.

Ito, K., Yamazaki, S., Yamamoto, R., Tajima, Y., Yanagida, A., Kobayashi, T., Kato-Itoh, M., Kakuta, S., Iwakura, Y., Nakauchi, H., and Kamiya, A. (2014). Gene targeting study reveals unexpected expression of brain-expressed X-linked 2 in endocrine and tissue stem/progenitor cells in mice. J. Biol. Chem. *289*, 29892–29911.

Kaestner, K.H. (2019). The intestinal stem cell niche: a central role for Foxl1-expressing subepithelial Telocytes. Cell. Mol. Gastroenterol. Hepatol. *8*, 111–117.

Karlsson, L., Lindahl, P., Heath, J.K., and Betsholtz, C. (2000). Abnormal gastrointestinal development in PDGF-A and PDGFR-(alpha) deficient mice implicates a novel mesenchymal structure with putative instructive properties in villus morphogenesis. Development *127*, 3457–3466.

Kim, J.E., Fei, L., Yin, W.C., Coquenlorge, S., Rao-Bhatia, A., Zhang, X., Shi, S.S.W., Lee, J.H., Hahn, N.A., Rizvi, W., et al. (2020). Single cell and genetic analyses reveal conserved populations and signaling mechanisms of gastrointestinal stromal niches. Nat. Commun. *11*, 334.

Kinchen, J., Chen, H.H., Parikh, K., Antanaviciute, A., Jagielowicz, M., Fawkner-Corbett, D., Ashley, N., Cubitt, L., Mellado-Gomez, E., Attar, M., et al. (2018). Structural remodeling of the human colonic mesenchyme in inflammatory bowel disease. Cell *175*, 372–386.e17.

Kolterud, A., Grosse, A.S., Zacharias, W.J., Walton, K.D., Kretovich, K.E., Madison, B.B., Waghray, M., Ferris, J.E., Hu, C., Merchant, J.L., et al. (2009). Paracrine Hedgehog signaling in stomach and intestine: new roles for hedgehog in gastrointestinal patterning. Gastroenterology *137*, 618–628.

Korinek, V., Barker, N., Moerer, P., van Donselaar, E., Huls, G., Peters, P.J., and Clevers, H. (1998). Depletion of epithelial stem-cell compartments in the small intestine of mice lacking Tcf-4. Nat. Genet. *19*, 379–383.

Kraiczy, J., Nayak, K., Ross, A., Raine, T., Mak, T.N., Gasparetto, M., Cario, E., Rakyan, V., Heuschkel, R., and Zilbauer, M. (2016). Assessing DNA methylation in the developing human intestinal epithelium: potential link to inflammatory bowel disease. Mucosal Immunol. *9*, 647–658.

Kraiczy, J., Nayak, K.M., Howell, K.J., Ross, A., Forbester, J., Salvestrini, C., Mustata, R., Perkins, S., Andersson-Rolf, A., Leenen, E., et al. (2019). DNA methylation defines regional identity of human intestinal epithelial organoids and undergoes dynamic changes during development. Gut *68*, 49–61.

Kurahashi, M., Niwa, Y., Cheng, J., Ohsaki, Y., Fujita, A., Goto, H., Fujimoto, T., and Torihashi, S. (2008). Platelet-derived growth factor signals play critical roles in differentiation of longitudinal smooth muscle cells in mouse embryonic gut. Neurogastroenterol. Motil. *20*, 521–531.

Li, N., van Unen, V., Abdelaal, T., Guo, N., Kasatskaya, S.A., Ladell, K., McLaren, J.E., Egorov, E.S., Izraelson, M., Chuva de Sousa Lopes, S.M., et al. (2019). Memory CD4+ T cells are generated in the human fetal intestine. Nat. Immunol. *20*, 301–312.

Lin, C.C., Bradstreet, T.R., Schwarzkopf, E.A., Sim, J., Carrero, J.A., Chou, C., Cook, L.E., Egawa, T., Taneja, R., Murphy, T.L., et al. (2014). Bhlhe40 controls cytokine production by T cells and is essential for pathogenicity in autoimmune neuroinflammation. Nat. Commun. *5*, 3551.

Lu, X., Yu, Y., and Tan, S. (2017). p53 expression in patients with ulcerative colitis - associated with dysplasia and carcinoma: a systematic meta-analysis. BMC Gastroenterol. *17*, 111.

Madison, B.B., Braunstein, K., Kuizon, E., Portman, K., Qiao, X.T., and Gumucio, D.L. (2005). Epithelial hedgehog signals pattern the intestinal crypt-villus axis. Development *132*, 279–289.

Mallow, E.B., Harris, A., Salzman, N., Russell, J.P., DeBerardinis, R.J., Ruchelli, E., and Bevins, C.L. (1996). Human enteric defensins. Gene structure and developmental expression. J. Biol. Chem. *271*, 4038–4045.

Martin, J.C., Chang, C., Boschetti, G., Ungaro, R., Giri, M., Grout, J.A., Gettler, K., Chuang, L.S., Nayar, S., Greenstein, A.J., et al. (2019). Single-cell analysis of Crohn's disease lesions identifies a pathogenic cellular module associated with resistance to anti-TNF therapy. Cell *178*, 1493–1508.e20.

McCarthy, N., Manieri, E., Storm, E.E., Saadatpour, A., Luoma, A.M., Kapoor, V.N., Madha, S., Gaynor, L.T., Cox, C., Keerthivasan, S., et al. (2020). Distinct mesenchymal cell populations generate the essential intestinal BMP signaling gradient. Cell Stem Cell *26*, 391–402.e5.

Moor, A.E., Harnik, Y., Ben-Moshe, S., Massasa, E.E., Rozenberg, M., Eilam, R., Bahar Halpern, K., and Itzkovitz, S. (2018). Spatial reconstruction of single enterocytes uncovers broad zonation along the intestinal villus axis. Cell *175*, 1156–1167.e15.

Muncan, V., Heijmans, J., Krasinski, S.D., Büller, N.V., Wildenberg, M.E., Meisner, S., Radonjic, M., Stapleton, K.A., Lamers, W.H., Biemond, I., et al. (2011). Blimp1 regulates the transition of neonatal to adult intestinal epithelium. Nat. Commun. *2*, 452.

Mustata, R.C., Vasile, G., Fernandez-Vallone, V., Strollo, S., Lefort, A., Libert, F., Monteyne, D., Pérez-Morga, D., Vassart, G., and Garcia, M.I. (2013). Identification of Lgr5-independent spheroid-generating progenitors of the mouse fetal intestinal epithelium. Cell Rep. *5*, 421–432.

Nowotschin, S., Setty, M., Kuo, Y.Y., Liu, V., Garg, V., Sharma, R., Simon, C.S., Saiz, N., Gardner, R., Boutet, S.C., et al. (2019). The emergent landscape of the mouse gut endoderm at single-cell resolution. Nature *569*, 361–367.

Olafsson, S., McIntyre, R.E., Coorens, T., Butler, T., Jung, H., Robinson, P.S., Lee-Six, H., Sanders, M.A., Arestang, K., Dawson, C., et al. (2020). Somatic evolution in non-neoplastic IBD-affected colon. Cell *182*, 672–684.e11.

Parikh, K., Antanaviciute, A., Fawkner-Corbett, D., Jagielowicz, M., Aulicino, A., Lagerholm, C., Davis, S., Kinchen, J., Chen, H.H., Alham, N.K., et al. (2019). Colonic epithelial cell diversity in health and inflammatory bowel disease. Nature *567*, 49–55.

Pedregosa, F., Varoquaux, G., Gramfort, A., Michel, V., Thirion, B., Grisel, O., Blondel, M., Müller, A., Nothman, J., Louppe, G., et al. (2011). Scikit-learn machine learning in Python. J. Mach. Learn. Res. *12*, 2825–2830.

Polański, K., Young, M.D., Miao, Z., Meyer, K.B., Teichmann, S.A., and Park, J.E. (2020). BBKNN: fast batch alignment of single cell transcriptomes. Bioinformatics *36*, 964–965.

Rao-Bhatia, A., Zhu, M., Yin, W.C., Coquenlorge, S., Zhang, X., Woo, J., Sun, Y., Dean, C.H., Liu, A., Hui, C.-C., et al. (2020). Hedgehog-activated Fat4 and PCP pathways mediate mesenchymal cell clustering and villus formation in gut development. Dev. Cell *52*, 647–658.e6.

CellPress
OPEN ACCESS

Sato, T., Stange, D.E., Ferrante, M., Vries, R.G., Van Es, J.H., Van den Brink, S., Van Houdt, W.J., Pronk, A., Van Gorp, J., Siersema, P.D., and Clevers, H. (2011a). Long-term expansion of epithelial organoids from human colon, adenoma, adenocarcinoma, and Barrett's epithelium. Gastroenterology *141*, 1762–1772.

Sato, T., van Es, J.H., Snippert, H.J., Stange, D.E., Vries, R.G., van den Born, M., Barker, N., Shroyer, N.F., van de Wetering, M., and Clevers, H. (2011b). Paneth cells constitute the niche for Lgr5 stem cells in intestinal crypts. Nature *469*, 415–418.

Schreurs, R.R.C.E., Baumdick, M.E., Sagebiel, A.F., Kaufmann, M., Mokry, M., Klarenbeek, P.L., Schaltenberg, N., Steinert, F.L., van Rijn, J.M., Drewniak, A., et al. (2019). Human fetal TNF-α-cytokine-producing CD4+ effector memory T cells promote intestinal development and mediate inflammation early in life. Immunity *50*, 462–476.e8.

Scialdone, A., Natarajan, K.N., Saraiva, L.R., Proserpio, V., Teichmann, S.A., Stegle, O., Marioni, J.C., and Buettner, F. (2015). Computational assignment of cell-cycle stage from single-cell transcriptome data. Methods *85*, 54–61.

Shoshkes-Carmel, M., Wang, Y.J., Wangensteen, K.J., Tóth, B., Kondo, A., Massasa, E.E., Itzkovitz, S., and Kaestner, K.H. (2018). Author Correction: subepithelial telocytes are an important source of Wnts that supports intestinal crypts. Nature *560*, E29.

Shyer, A.E., Huycke, T.R., Lee, C., Mahadevan, L., and Tabin, C.J. (2015). Bending gradients: how the intestinal stem cell gets its home. Cell *161*, 569–580.

Shyer, A.E., Tallinen, T., Nerurkar, N.L., Wei, Z., Gil, E.S., Kaplan, D.L., Tabin, C.J., and Mahadevan, L. (2013). Villification: how the gut gets its villi. Science *342*, 212–218.

Smillie, C.S., Biton, M., Ordovas-Montanes, J., Sullivan, K.M., Burgin, G., Graham, D.B., Herbst, R.H., Rogel, N., Slyper, M., Waldman, J., et al. (2019). Intra- and inter-cellular rewiring of the human colon during ulcerative colitis. Cell *178*, 714–730.e22.

Sonntag, B., Stolze, B., Heinecke, A., Luegering, A., Heidemann, J., Lebiedz, P., Rijcken, E., Kiesel, L., Domschke, W., Kucharzik, T., and Maaser, C. (2007). Preterm birth but not mode of delivery is associated with an increased risk of developing inflammatory bowel disease later in life. Inflam. Bowel Dis. *13*, 1385–1390.

Spence, J.R., Lauf, R., and Shroyer, N.F. (2011). Vertebrate intestinal endoderm development. Dev. Dyn. *240*, 501–520.

Strimmer, K. (2008). fdrtool: a versatile R package for estimating local and tail area-based false discovery rates. Bioinformatics *24*, 1461–1462.

Svensson, V., and Pachter, L. (2018). RNA velocity: molecular kinetics from single-cell RNA-seq. Mol. Cell *72*, 7–9.

Takaishi, H., Kanai, T., Nakazawa, A., Sugata, F., Nikai, A., Yoshizawa, S., Hamamoto, Y., Funakoshi, S., Yajima, T., Iwao, Y., et al. (2012). Anti-high mobility group box 1 and box 2 non-histone chromosomal proteins (HMGB1/HMGB2) antibodies and anti-Saccharomyces cerevisiae antibodies (ASCA): accuracy in differentially diagnosing UC and CD and correlation with inflammatory bowel disease phenotype. J. Gastroenterol. *47*, 969–977.

Teng, Y.-S., Zhao, Y.-L., Li, M.-S., Liu, Y.-G., Cheng, P., Lv, Y.-P., Mao, F.-Y., Chen, W., Yang, S.-M., Hao, C.-J., et al. (2020). Upexpression of BHLHE40 in gastric epithelial cells increases CXCL12 production through interaction with p-STAT3 in Helicobacter pylori-associated gastritis. FASEB J. *34*, 1169–1181.

Traag, V.A., Waltman, L., and van Eck, N.J. (2019). From Louvain to Leiden: guaranteeing well-connected communities. Sci. Rep. *9*, 5233.

Tsai, Y.H., Nattiv, R., Dedhia, P.H., Nagy, M.S., Chin, A.M., Thomson, M., Klein, O.D., and Spence, J.R. (2017). In vitro patterning of pluripotent stem cell-derived intestine recapitulates in vivo human development. Development *144*, 1045–1055.

Vento-Tormo, R., Efremova, M., Botting, R.A., Turco, M.Y., Vento-Tormo, M., Meyer, K.B., Park, J.E., Stephenson, E., Polański, K., Goncalves, A., et al. (2018). Single-cell reconstruction of the early maternal-fetal interface in humans. Nature *563*, 347–353.

Vitali, R., Stronati, L., Negroni, A., Di Nardo, G., Pierdomenico, M., del Giudice, E., Rossi, P., and Cucchiara, S. (2011). Fecal HMGB1 is a novel marker of intestinal mucosal inflammation in pediatric inflammatory bowel disease. Am. J. Gastroenterol. *106*, 2029–2040.

Walton, K.D., Kolterud, A., Czerwinski, M.J., Bell, M.J., Prakash, A., Kushwaha, J., Grosse, A.S., Schnell, S., and Gumucio, D.L. (2012). Hedgehog-responsive mesenchymal clusters direct patterning and emergence of intestinal villi. Proc. Natl. Acad. Sci. USA *109*, 15817–15822.

Walton, K.D., Freddo, A.M., Wang, S., and Gumucio, D.L. (2016a). Generation of intestinal surface: an absorbing tale. Development *143*, 2261–2272.

Walton, K.D., Whidden, M., Kolterud, Å., Shoffner, S.K., Czerwinski, M.J., Kushwaha, J., Parmar, N., Chandhrasekhar, D., Freddo, A.M., Schnell, S., and Gumucio, D.L. (2016b). Villification in the mouse: Bmp signals control intestinal villus patterning. Development *143*, 427–436.

Wang, Y., Chiang, I.L., Ohara, T.E., Fujii, S., Cheng, J., Muegge, B.D., Ver Heul, A., Han, N.D., Lu, Q., Xiong, S., et al. (2019). Long-term culture captures injury-repair cycles of colonic stem cells. Cell *179*, 1144–1159.e15.

Wehkamp, J., Wang, G., Kübler, I., Nuding, S., Gregorieff, A., Schnabel, A., Kays, R.J., Fellermann, K., Burk, O., Schwab, M., et al. (2007). The Paneth Cell α-Defensin Deficiency of Ileal Crohn's disease Is Linked to Wnt/Tcf-4. J. Immunol. *179*, 3109–3118.

Wolf, F.A., Angerer, P., and Theis, F.J. (2018). SCANPY: large-scale single-cell gene expression data analysis. Genome Biol. *19*, 15.

Wolf, F.A., Hamey, F.K., Plass, M., Solana, J., Dahlin, J.S., Göttgens, B., Rajewsky, N., Simon, L., and Theis, F.J. (2019). PAGA: graph abstraction reconciles clustering with trajectory inference through a topology preserving map of single cells. Genome Biol. *20*, 59.

Wolock, S.L., Lopez, R., and Klein, A.M. (2019). Scrublet: computational identification of cell doublets in single-cell transcriptomic data. Cell Syst *8*, 281–291.e9.

Wu, Y., Kimura, Y., Okamoto, T., Matsuhisa, K., Asada, R., Saito, A., Sakaue, F., Imaizumi, K., and Kaneko, M. (2019). Inflammatory bowel disease-associated ubiquitin ligase RNF183 promotes lysosomal degradation of DR5 and TRAIL-induced caspase activation. Sci. Rep. *9*, 20301.

Xian, L., Georgess, D., Huso, T., Cope, L., Belton, A., Chang, Y.T., Kuang, W., Gu, Q., Zhang, X., Senger, S., et al. (2017). HMGA1 amplifies Wnt signalling and expands the intestinal stem cell compartment and Paneth cell niche. Nat. Commun. *8*, 15008.

Yanai, H., Atsumi, N., Tanaka, T., Nakamura, N., Komai, Y., Omachi, T., Tanaka, K., Ishigaki, K., Saiga, K., Ohsugi, H., et al. (2017). Intestinal stem cells contribute to the maturation of the neonatal small intestine and colon independently of digestive activity. Sci. Rep. *7*, 9891.

Ye, H., Kelly, T.F., Samadani, U., Lim, L., Rubio, S., Overdier, D.G., Roebuck, K.A., and Costa, R.H. (1997). Hepatocyte nuclear factor 3/fork head homolog 11 is expressed in proliferating epithelial and mesenchymal cells of embryonic and adult tissues. Mol. Cell. Biol. *17*, 1626–1641.

Yu, F., Sharma, S., Jankovic, D., Gurram, R.K., Su, P., Hu, G., Li, R., Rieder, S., Zhao, K., Sun, B., and Zhu, J. (2018). The transcription factor Bhlhe40 is a switch of inflammatory versus antiinflammatory Th1 cell fate determination. J. Exp. Med. *215*, 1813–1821.

Yu, Q., Kilik, U., Holloway, E.M., Tsai, Y.-H., Wu, A., Wu, J.H., Czerwinski, M., Childs, C., He, Z., Glass, I.A., et al. (2020). An organoid and multi-organ developmental cell atlas reveals multilineage fate specification in the human intestine bioRxiv. https://doi.org/10.1101/2020.07.24.219147.

Yui, S., Azzolin, L., Maimets, M., Pedersen, M.T., Fordham, R.P., Hansen, S.L., Larsen, H.L., Guiu, J., Alves, M.R.P., Rundsten, C.F., et al. (2018). YAP/TAZ-dependent reprogramming 7of colonic epithelium links ECM remodeling to tissue regeneration. Cell Stem Cell *22*, 35–49.e7.

Zhang, C., Zhu, X., Hua, Y., Zhao, Q., Wang, K., Zhen, L., Wang, G., Lü, J., Luo, A., Cho, W.C., et al. (2019). YY1 mediates TGF-β1-induced EMT and pro-fibrogenesis in alveolar epithelial cells. Respir. Res. *20*, 249.

Zheng, G.X.Y., Terry, J.M., Belgrader, P., Ryvkin, P., Bent, Z.W., Wilson, R., Ziraldo, S.B., Wheeler, T.D., McDermott, G.P., Zhu, J., et al. (2017). Massively parallel digital transcriptional profiling of single cells. Nat. Commun. *8*, 14049.

STAR★METHODS

KEY RESOURCES TABLE

REAGENT/ RESOURCE	SOURCE	IDENTIFIER
RNAscope probes		
BEX5	ACD, Bio-Techne	581118
CFTR	ACD, Bio-Techne	603298-C2
F3	ACD, Bio-Techne	407618-C2
FOXL1	ACD, Bio-Techne	558088-C3
LGR5	ACD, Bio-Techne	311028-C2
MKI67	ACD, Bio-Techne	591778-C3
PDGFA	ACD, Bio-Techne	406728-C4
PDGFRA	ACD, Bio-Techne	604488
PLA2G2A	ACD, Bio-Techne	581108-C4
PTCH1	ACD, Bio-Techne	405788
SHH	ACD, Bio-Techne	600958-C2
TACSTD2	ACD, Bio-Techne	405478-C4
UPK3B	ACD, Bio-Techne	581098-C4
WNT2B	ACD, Bio-Techne	453368
RNAscope reagents		
RNAscope Multiplex Fluorescent Reagent Kit	ACD, Bio-techne	322800
RNAscope 4-plex Ancillary Kit for Multiplex Fluorescent Reagent Kit	ACD, Bio-techne	322830
Opal 520	Akoya Biosciences	FP1487001KT
Opal 570	Akoya Biosciences	FP1488001KT
Opal 650	Akoya Biosciences	FP1496001KT
TSA-biotin	Akoya Biosciences	NEL749A001KT
Streptavidin-conjugated Atto 425	Sigma Aldrich	09260-1MG-F
Biological Samples		
Human fetal intestinal tissue	University of Cambridge	Acquired directly from ethically approved internal study (REC-96/085)
Chemicals, Peptides, and Recombinant Proteins		
Chromium Single Cell 3' Library & Gel Bead Kit v2	10x Genomics	Catalogue # PN-120237
WNT3A	Conditioned cell line (internal)	N/A
R-Spondin-1	Conditioned cell line (internal)	N/A
EGF	Invitrogen	PHG0313
A8301	Tocris	2939
Y27632	Sellech Chem	S1049
Noggin	N/A	N/A
B27	Thermo Fisher Scientific	17504044
HEPES buffer	Gibco (Life Technologies)	15630080
Glutamax	Gibco (Life Technologies)	35050061
DMEM-F12	Gibco (Life Technologies)	11320033
Liberase DH	Sigma Aldrich	5401054001
WNT2B	Abcam	ab132538
Hyaluronidase	Merck	HX0514

(*Continued on next page*)

Continued

REAGENT/ RESOURCE	SOURCE	IDENTIFIER
Deposited Data		
Fetal, pediatric and organoid single cell RNAseq data	This study	https://www.ebi.ac.uk/arrayexpress/experiments/E-MTAB-8901/
Single cell RNAseq data	GEO: GSE134809	Martin et al., 2019
Single cell RNAseq data	GEO: GSE114374	Kinchen et al., 2018
Software and Algorithms		
Cellranger 10x Genomics, version 2.1.1 (reference transcriptome GRCh38-1.2.0)	https://support.10xgenomics.com/single-cell-gene-expression/software/pipelines/latest/installation	Zheng et al., 2017
Scanpy package v1.4	https://icb-scanpy.readthedocs-hosted.com/en/stable/	Wolf et al., 2018
Scrublet	https://github.com/AllonKleinLab/scrublet	Wolock et al., 2019
human_cycle_markers.rds, scran package	https://github.com/MarioniLab/scran/tree/master/inst/exdata	Scialdone et al., 2015
BBKNN	https://bbknn.readthedocs.io/en/latest/	Polański et al., 2020
UMAP	https://scanpy.readthedocs.io/en/stable/api/scanpy.tl.umap.html	Becht et al., 2018
Leiden	https://scanpy.readthedocs.io/en/stable/api/scanpy.tl.leiden.html	Traag et al., 2019
scVelo 1.24 package	https://github.com/theislab/scvelo	Bergen et al., 2020
PAGA	https://github.com/theislab/paga	Wolf et al., 2019
CellPhoneDB v2.0	https://www.cellphonedb.org/	Efremova et al., 2020
Sklearn implementation linear_model.LogisticRegression	https://scikit-learn.org/stable/modules/generated/sklearn.linear_model.LogisticRegression.html	Pedregosa et al., 2011
fdrtool package (R version 3.5.0)	https://cran.r-project.org/web/packages/fdrtool/fdrtool.pdf	Strimmer, 2008
Pandas, Version 0.25.2	https://pandas.pydata.org/	N/A
NumPy, Version 1.16.2	https://pypi.org/project/numpy/1.16.2/	N/A
Anndata	https://pypi.org/project/anndata/	N/A
Scipy	https://www.scipy.org/	N/A

RESOURCE AVAILABILITY

Lead Contact

Further information and requests for resources and reagents should be directed to and will be fulfilled by the Lead Contact; Matthias Zilbauer (mz304@medschl.cam.ac.uk).

Materials Availability

Human cell lines developed in this study can only be distributed following MTA arrangement.

Data and Code Availability

The accession number for the raw sequencing data reported in this paper is E-MTAB-8901. Processed single-cell RNA sequencing objects will be available for online visualisation and download at gutcellatlas.org. The code generated during this study will be available at Github https://github.com/Raselel/DevCell_GutAtlas/.

EXPERIMENTAL MODEL AND SUBJECT DETAILS

Fetal and Paediatric Tissue Sampling

First trimester human fetal tissue was collected from patients undergoing elective termination of pregnancy. Patients gave informed consent as part of the ethically approved research study (REC-96/085). Fetal age (post conception weeks, PCW) was estimated using the independent measurement of the crown rump length (CRL), using the formula PCW (days) = 0.9022 × CRL (mm) + 27.372. Fetal sample ages in post-conception weeks were as follows: BRC2029 - 6.1, BRC2026 - 8.4, BRC2043 - 10.2, BRC2046 - 6.7,

BRC2049- 6.9, BRC2121 - 9.2, BRC2119- 7.9, BRC2133 - 9.9, BRC2134- 10. Organoids were derived from fetal tissues BRC2038- 6.4, BRC2039 - 5.4, BRC2206- 6.5.

Human intestinal mucosal biopsies were obtained from patients undergoing colonoscopy at Addenbrooke's Hospital, Cambridge, UK. All patients gave informed consent for extra biopsy samples to be taken for research use when undergoing elective colonoscopy (REC 17/EE/0265). Patients were then included if, after macroscopic visualization and histological analysis, they were diagnosed as either having CD, or being without an inflammatory diagnosis (control). Patient ages were as follows: Control group (T036- 4 years, T110- 4 years, T161- 4 years, T057- 6 years, T182- 9 years, T44- 10 years, T024- 12 years, T160- 10 years); CD group (T197- 9 years, T176- 11 years, T019- 12 years, T202- 12 years, T017-13 years, T189-13 years, T203-14 years).

METHOD DETAILS

Fetal and Paediatric Tissue Dissociation

Fetal intestinal samples were dissected into duodenum-jejunum (further referred to as the duo-jejunum), ileum and colon using anatomical landmarks, and processed to single-cell suspension in parallel. Both fetal samples and paediatric samples were processed using the same protocol. Briefly, paediatric biopsies or fetal tissue sections were immediately rinsed twice with Hank's Balanced Salt Solution (HBSS) medium (Sigma-Aldrich) and digested in HBSS medium containing 1.07 Wünsch units/ml of Liberase DH (Roche) and 600 IU of Hyaluronidase (Calbiochem) on a shaking platform (750 rpm) at 37°C for up to 30 min. The tissue was gently homogenised using a P1000 pipette every 15 mins. A single-cell suspension was then passed through a 40 μm cell strainer to remove undigested tissue. Cells were spun down at 400 g at 4°C for 5 min and the pelleted cells were washed in DMEM/F12 three times using centrifugation.

Fetal cells were either loaded for scRNAseq directly following sample processing or subjected to EPCAM selection to enrich epithelial cells. For enrichment, single cells were suspended in MACS modified solution (PBS with 0.5% BSA, 2 mM EDTA and 100 IU/mL DNaseII) and stained with EPCAM (CD326) magnetic microbeads (Milteny biotec) according to the manufacturer's protocol. Enrichment was performed using an autoMACS Pro Separator. Either only EPCAM positive (PCW 6.7, 6.9, 10.2, 9.3) or both EPCAM positive and negative (PCW 9.9, 10.1, 10) fractions were processed using the 10x Genomics single-cell transcriptomics system. All paediatric single-cell suspensions were subjected to the MACS enrichment using the same protocol as described for fetal samples. Both fetal and paediatric single-cell suspensions were carried forward into single-cell sequencing only if the viability was >60% (Table S1).

Intestinal Organoid Culture

Fetal organoids were cultured in Matrigel® (Corning) using media described in (Fordham et al., 2013) and also provided in Table S2. During organoid culture, the media was replaced every 4872hours. Organoids were passaged using mechanical disruption with a P1000 pipette and re-seeded in fresh growth-factor reduced Matrigel® (Corning). When comparing culture media, multiple wells were seeded from a single dissociated sample, and wells assigned to either of the media. Organoid lines grown in WNT2B were grown in identical conditions, but with WNT3A replaced by recombinant human WNT2B at 100ng/μl (Abcam).

Organoids were derived from fetal ileum from embryos aged 5.5 and 6.4 PCW (BRC2038, BRC2039) and were maintained *in vitro* for 17 passages. Organoids were dissociated for single cell RNA-sequencing at passage 1 (1 week) or passage 17 (~4 months) in culture and profiled using 10x Genomics single-cell transcriptomics. For the WNT2B/WNT3A comparison experiment, cells from fetal ileum from an embryo at 6.4 PCW (BRC2206) were used to generate organoid lines in either WNT2B and WNT3A, with dissociation for single cell analysis performed five days after the first passage. Processing for single-cell sequencing analysis was performed by removing the organoids from matrigel using incubation with Cell Recovery Solution at 4°C for 20 minutes, pelleting the cells, and re-suspending in TrypLE enzyme solution (Thermo Fisher) for incubation at 37°C for 10 mins. Cells were pelleted again and re-suspended in DMEM/F12. Brightfield images of organoids were taken using an EVOS Cell Imaging Systems microscope (Thermo Fisher).

Tissue Freezing, Sectioning and RNAscope

Tissue was prepared, stained, and imaged largely as described previously (Bayraktar et al., 2020). In short, fresh tissue samples were either embedded in OCT and frozen at -80°C on an isopentane-dry ice slurry, or fixed in 10% neutral-buffered formalin at 4°C for ~24 hours, and then embedded and frozen as above. Cryosections were cut at a thickness of 10-16 μm using a Leica CM3050 S cryostat and placed onto SuperFrost®Plus™ slides (VWR). Prior to staining, tissue sections were post-fixed in 4% paraformaldehyde in PBS for 15 minutes at 4°C, then dehydrated through a series of 50%, 70%, 100%, and 100% ethanol, for 5 minutes each.

Tissue sections were then processed using a Leica BOND RX to automate staining with the RNAscope® Multiplex Fluorescent Reagent Kit v2 Assay and RNAscope® 4-plex Ancillary Kit for Multiplex Fluorescent Reagent Kit v2 (Advanced Cell Diagnostics, Bio-Techne), according to the manufacturers' instructions. Automated processing of non-fixed sections included pre-treatment with Protease IV for 30 minutes, but no heat treatment; fixed frozen sections were subjected to heat-induced epitope retrieval at 95°C in buffer ER2, and digestion with Protease III for 15 minutes. Tyramide signal amplification with Opal 520, Opal 570, and Opal 650 (Akoya Biosciences) was used to develop three probe channels. The fourth was developed using TSA-biotin (TSA Plus Biotin Kit, Perkin Elmer) and streptavidin-conjugated Atto 425 (Sigma Aldrich).

Stained sections were imaged with a Perkin Elmer Opera® Phenix™ High-Content Screening System, in confocal mode with 1 μm z-step size, using 20× (NA 0.16, 0.299 μm/pixel) and 40× (NA 1.1, 0.149 μm/pixel) water-immersion objectives.

Single-cell RNA-sequencing

Single-cell suspension for each primary intestinal or organoid sample was loaded onto a separate channel of a Chromium 10x Genomics single cell 3'v2 library chip as per manufacturer's protocol (10x Genomics; PN-120233), aiming for a cell capture recovery of 3000-5000 cells. cDNA sequencing libraries were prepared according to the manufacturer's protocol and sequenced on an Illumina Hi-seq 4000 (2x50bp paired-end reads).

Processing FASTQ Files and Quality Control

Raw sequence reads in FASTQ format from fetal, paediatric and organoid samples were processed and aligned to the GRCh38-1.2.0 human reference transcriptome using the Cellranger v2.1.1 pipeline (10x Genomics) with default parameters.

The resulting gene expression matrices integrated together using Scanpy package v1.4 (Wolf et al., 2018). A total of 34 fetal sample count matrices were merged together. Separately, 15 gene expression matrices of healthy and CD paediatric biopsy samples were merged together for cell annotation and direct comparisons. Organoid gene expression matrices from the same experiment were also merged separately.

The pre-processing followed the guidelines provided by Scanpy V1.4 tutorial (Wolf et al., 2018). In short, entries with fewer than 200 genes and greater than 9000 total genes were filtered to remove empty droplets and probable doublets, respectively. The distribution of Unique Molecular Identifiers (UMIs) and genes per cell were visualised using scanpy pl.scatter function (Figures S1A–S1C). To account for differences in sequencing depth across samples, we normalised expression values for total UMIs per cell and log transformed the counts.

Doublet Removal

All 10x runs were processed using Scrublet doublet detection pipeline with threshold of 0.25-0.3 (Wolock et al., 2019), and predicted doublets were excluded from the analysis. We further annotated doublets by sub-clustering the data and identifying clusters with gene expression of other clusters. In fetal samples the doublets were largely epithelial-mesenchymal and neuronal-mesenchymal. In paediatric samples, we found mostly T cell-enterocyte and Goblet cell-enterocyte doublets.

Clustering, Visualisation and Cell Annotation

For cell clustering we used highly variable genes selected using sc.pp.highly_variable_genes function with default parameters. In addition, cell-cycle signatures were determined using cycle stage marker genes imported from human_cycle_markers.rds, scran package (Scialdone et al., 2015) and removed from highly variable genes of the full dataset. Similarly, ribosomal protein genes were removed from the highly variable genes as they contributed to the highest variability in the F6.1 sample. In addition, UMI counts, percentage of mitochondrial genes were considered to be the source of unwanted variability and were regressed using Scanpy regress_out function (Wolf et al., 2018).

To remove variation of each 10X Genomics run and maintain the development related biological variation, we used batch balanced k nearest neighbour (BBKNN) method (Polański et al., 2020) on 40 principal components and trim parameter set to 20. Dimensionality reduction was performed on remaining highly variable genes and cells were visualised using Uniform Manifold Approximation and Projection (UMAP) plots (Becht et al., 2018). We then used Scanpy implementation of Leiden algorithm for unsupervised clustering of the data (Traag et al., 2019). Clusters were annotated using markers genes found in the literature in combination with differentially expressed genes (Wilcoxon test, function sc.tl.rank_genes_groups). Paediatric healthy and CD samples were annotated together, in order to draw direct comparisons. Marker gene expression was visualised using dot-plots where the size of the dot reflects the percentage of cells expressing the gene and color indicates relative expression.

scVelo AND PAGA TRAJECTORY ANALYSIS

Fetal epithelial cell dynamics in small bowel samples were analysed using scVelo 1.24 package implementation in Scanpy 1.4.5 (Bergen et al., 2020; Svensson and Pachter, 2018). The data was sub-clustered to epithelial cells and split into two groups 6–8 PCW (including F6.7, F6.9, F.7.9), and 9-10 PCW (including F9.9, F10, F10.1, F10.2). The clustering and visualisation was repeated using the same parameters as above for the sub-clustered cells. The data was then processed using default parameters following preprocessing as described in Scanpy scVelo implementation.

In short, the gene-specific velocities are obtained by fitting a ratio between unspliced and spliced mRNA abundances and then computing how the observed abundances change from those observed in steady state. The ratio of 'spliced', 'unspliced', and 'ambiguous' transcripts was calculated to be 0.67, 0.25, 0.07, and 0.76, 0.17, 0.06 for 6-8 PCW and 9-10 PCW groups, respectively. The samples were pre-processed using functions for detection of minimum number of counts, filtering and normalisation using scv.pp.filter_and_normalise and followed by scv.pp.moments function. The gene specific velocities were then calculated using scv.tl.velocity with mode set to stochastic, and scv.tl.velocity_graph functions) and visualised using scv.pl.velocity_graph function. In addition, we used tl.recover_latent_time function to infer a shared latent time from splicing dynamics and plotted the genes along time axis sorted by expression along dynamics using scv.pl.heatmap function.

For Partition-based graph abstraction (PAGA) analysis (Wolf et al., 2019), we use scanpy implementation, sc.tl.draw_graph (init_pos='paga') followed by sc.tl.paga (threshold=0.3) and sc.pl.paga function for analysis and plotting, and similarly Scanpy scVelo implementation function scv.tl.paga with default parameters and scv.pl.paga function for velocity-driven paga analysis and plotting.

Inferring Cell-Cell Interactions

To infer cell-cell interactions we applied the CellPhoneDB v2.0 python package (Vento-Tormo et al., 2018; Efremova et al., 2020) to four separate datasets: 1) embryonic cells from duo-jejunum and ileum; 2) fetal cells from duo-jejunum and ileum; 3) healthy paediatric samples; and 4) CD samples. Log transformed and normalised counts, and cell type annotations were used as an input. To narrow down the most relevant interactions, we looked for specific interactions classified by ligand/receptor expression in more than 10% of cells within a cluster and where $\log_2$ mean expression of ligand/receptor pair is greater than 0.

Cellular Composition Classification

Raw counts for Kinchen et al. (2018) and Martin et al. (2019) were downloaded from GEO website and processed-annotated according to the original publication. The python package Sklearn implementation linear_model.LogisticRegression (Pedregosa et al., 2011) was used to predict the cellular composition and temporal identity of either the organoid datasets (Figures 5F and 5L) or pediatric datasets (Figure S6). In case of organoids, expression matrix and annotation labels of all primary cells from the developing small bowel were used as an input for training the model. For prediction of fetal stromal cells, the model was trained on healthy biopsy data from Kinchen et al. (2018). For pediatric data predictions, expression matrix and annotation labels of either healthy biopsy data from Kinchen et al. (2018), or healthy pediatric ileal cells from this study were used as an input for training the model. In all cases above, we used C=0.20, solver='saga' and penalty set to L1 to favour sparsity in the scRNAseq expression matrix. The classifier estimated sparsity was over 95% and lr.score was over 0.9. We took into account predictions with probability higher than 80% and used top labels to calculate relative abundance of predicted cell types. The relative abundance of predicted cell-types in organoids was shown as percentage of cells per experimental condition (p1 vs p17, or WNT3A- vs WNT3A+).

Transcription Factors in Epithelium

For comparisons between fetal and paediatric epithelium, we merged and analysed cells collected from the matching anatomical location (fetal terminal ileum only) and enriched using the same strategy. First, we selected all transcription factors (TF) based on a list obtained from (http://bioinfo.life.hust.edu.cn/AnimalTFDB/#!/download) and used Scanpy sc.tl.rank_genes_groups function (Wilcoxon test) to select differentially expressed TF (from total of 1529 expressed TF) between inflamed CD (Figure S5, arrows) versus control and non-inflamed epithelium (5F, bottom barplot, samples with no arrows). Out of these, we selected TFs that were differentially expressed in CD patients and upregulated in fetal epithelium and plotted their relative mean expression as a heatmap using sns.clustermap, z-score calculated for genes (rows).

Crypt-Villus Axis and HH Signalling Score

The axis score was derived by using the expression of selected crypt-villus axis markers as defined by Moor et al. (2018) and Parikh et al. (2019) (SEPP1, CEACAM7, PLAC8, CEACAM1, TSPAN1, CEACAM5, CEACAM6, IFI27, DHRS9, KRT20, RHOC, CD177, PKIB, HPGD, LYPD8, APOBEC1, APOB, APOA4, APOA1, NPC1L1, EGFR, KLF4, ENPP3, NT5E, SLC28A2, ADA). Similarly, we use selected genes for the scoring of HH pathway activation (*PTCH1*, *PTCH2*, *GLI1*, *GLI2*, *GLI3*, *SMO*). The scoring was done using sc.tl.score_genes() function with default parameters to calculate the average expression of selected genes substrated with the average expression of reference genes.

QUANTIFICATION AND STATISTICAL ANALYSIS

Percentage of Cells and Statistical Analysis

First, we calculated relative abundance of each epithelial cell type as percentage of cells per condition (control and CD, fetal ileum and fetal duodenum). We test for statistical significance using two-tailed t.test (R version 3.5.0) and report the p-values as extracted by fdrtool package (statistics = p_values). To assess regional contribution to epithelial clusters, we used total number of cells and two-way ANOVA for multiple comparisons (GraphPad).

Developmental Cell, Volume *55*

Supplemental Information

Single-Cell Sequencing of Developing Human Gut Reveals Transcriptional Links to Childhood Crohn's Disease

Rasa Elmentaite, Alexander D.B. Ross, Kenny Roberts, Kylie R. James, Daniel Ortmann, Tomás Gomes, Komal Nayak, Liz Tuck, Sophie Pritchard, Omer Ali Bayraktar, Robert Heuschkel, Ludovic Vallier, Sarah A. Teichmann, and Matthias Zilbauer

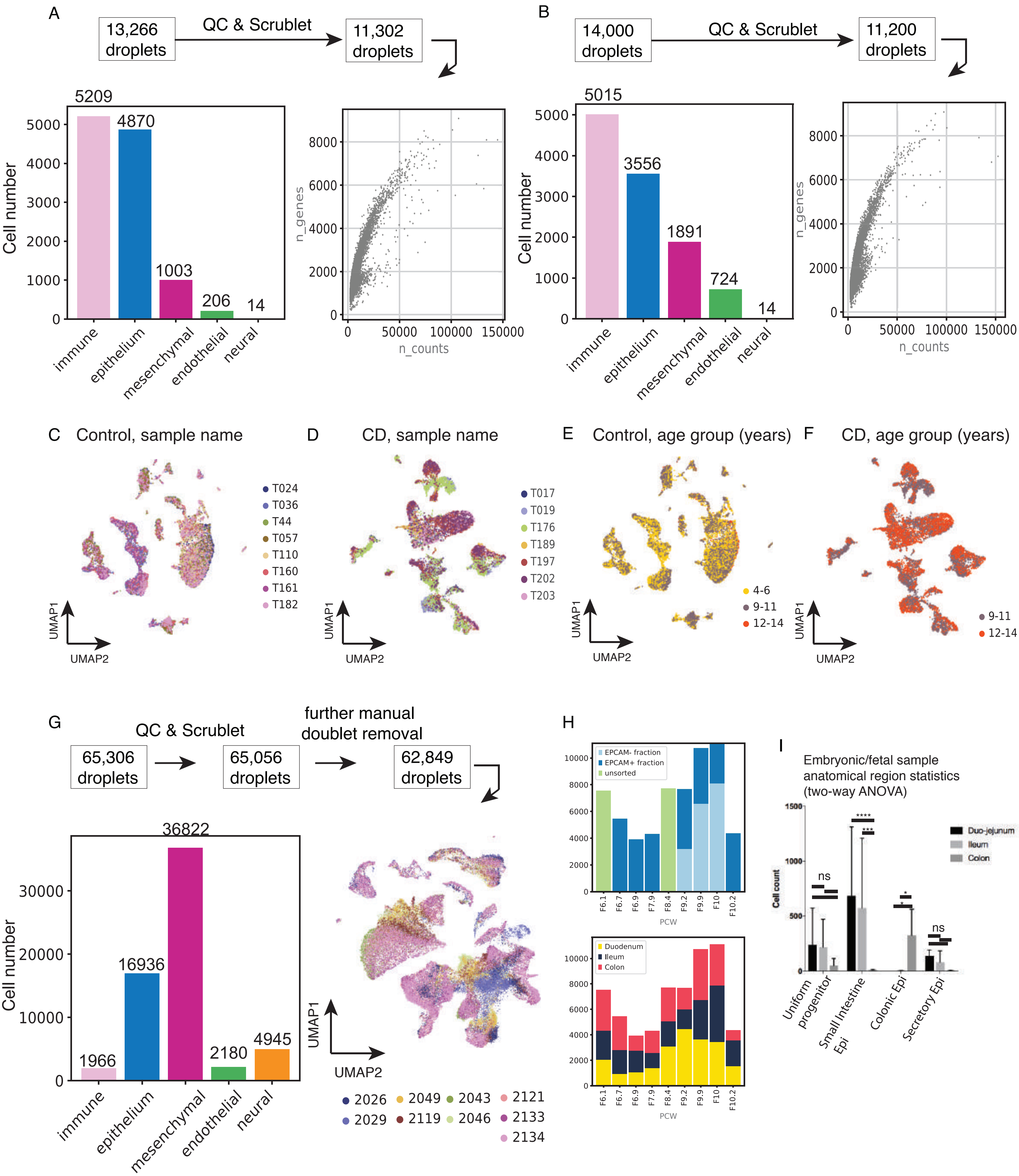

A
13,266 droplets
QC & Scrublet
11,302 droplets
Cell number
5209
4870
1003
206
14
immune
epithelium
mesenchymal
endothelial
neural
n_genes
n_counts
B
14,000 droplets
QC & Scrublet
11,200 droplets
5015
3556
1891
724
14
C Control, sample name
T024
T036
T44
T057
T110
T160
T161
T182
D CD, sample name
T017
T019
T176
T189
T197
T202
T203
E Control, age group (years)
4-6
9-11
12-14
F CD, age group (years)
9-11
12-14
UMAP1
UMAP2
G
QC & Scrublet
65,306 droplets
65,056 droplets
further manual doublet removal
62,849 droplets
36822
16936
1966
2180
4945
2026
2029
2049
2119
2043
2046
2121
2133
2134
H
EPCAM- fraction
EPCAM+ fraction
unsorted
PCW
Duodenum
Ileum
Colon
I
Embryonic/fetal sample anatomical region statistics (two-way ANOVA)
Cell count
Duo-jejunum
Ileum
Colon
ns
Uniform progenitor
Small Intestine Epi
Colonic Epi
Secretory Epi

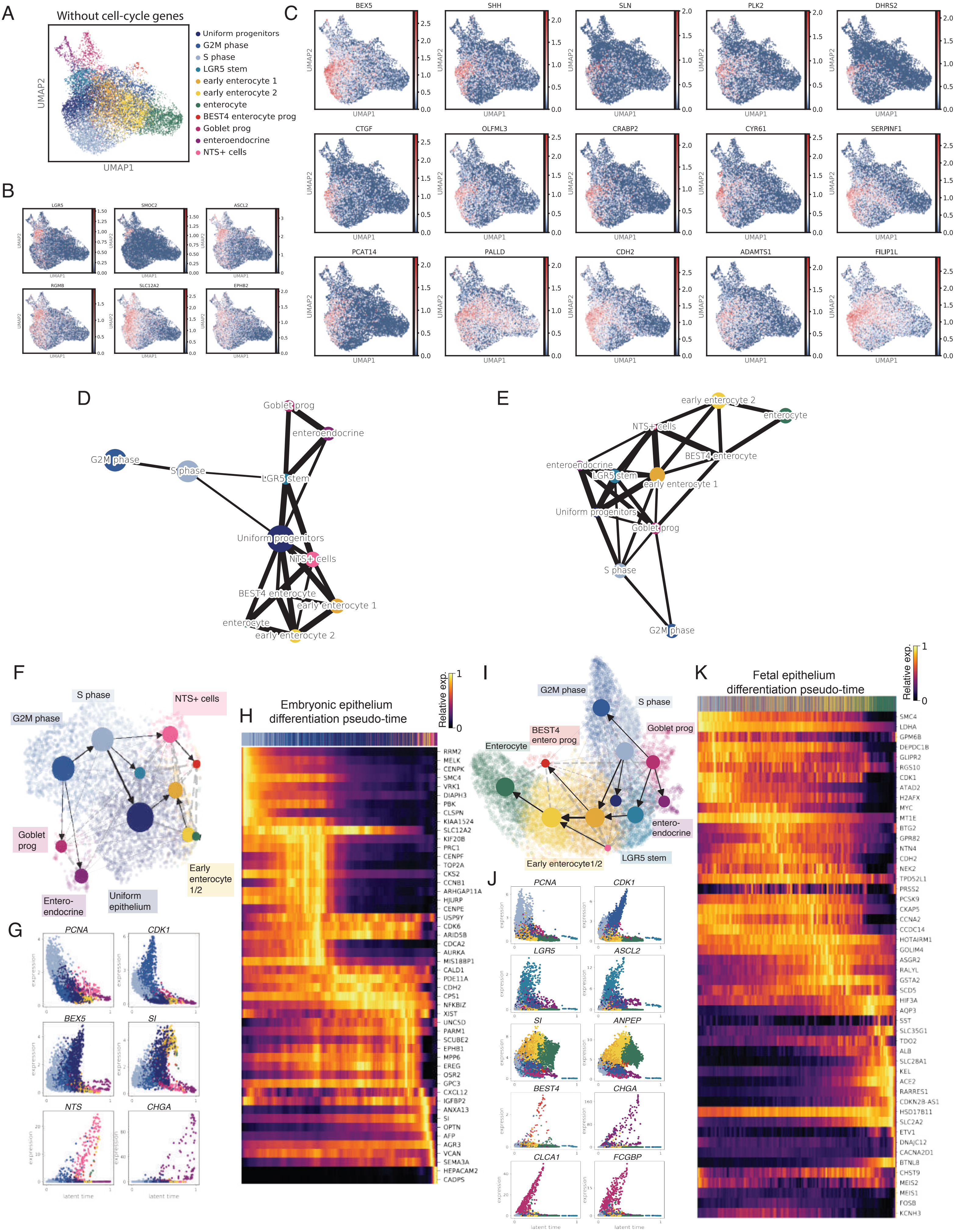

A
Without cell-cycle genes
UMAP2
UMAP1
Uniform progenitors
G2M phase
S phase
LGR5 stem
early enterocyte 1
early enterocyte 2
enterocyte
BEST4 enterocyte prog
Goblet prog
enteroendocrine
NTS+ cells
B
LGR5
SMOC2
ASCL2
RGMB
SLC12A2
EPHB2
C
BEX5
SHH
SLN
PLK2
DHRS2
CTGF
OLFML3
CRABP2
CYR61
SERPINF1
PCAT14
PALLD
CDH2
ADAMTS1
FILIP1L
D
Goblet prog
enteroendocrine
G2M phase
S phase
LGR5 stem
Uniform progenitors
NTS+ cells
BEST4 enterocyte
early enterocyte 1
enterocyte
early enterocyte 2
E
early enterocyte 2
enterocyte
NTS+ cells
BEST4 enterocyte
enteroendocrine
LGR5 stem
early enterocyte 1
Uniform progenitors
Goblet prog
S phase
G2M phase
F
S phase
NTS+ cells
G2M phase
Goblet prog
Entero-endocrine
Uniform epithelium
Early enterocyte 1/2
G
PCNA
CDK1
BEX5
SI
NTS
CHGA
expression
latent time
H
Embryonic epithelium differentiation pseudo-time
Relative exp.
1
0
RRM2
MELK
CENPK
SMC4
VRK1
DIAPH3
PBK
CLSPN
KIAA1524
SLC12A2
KIF20B
PRC1
CENPF
TOP2A
CKS2
CCNB1
ARHGAP11A
HJURP
CENPE
USP9Y
CDK6
ARID5B
CDCA2
AURKA
MIS18BP1
CALD1
PDE11A
CDH2
CPS1
NFKBIZ
XIST
UNC5D
PARM1
SCUBE2
EPHB1
MPP6
EREG
OSR2
GPC3
CXCL12
IGFBP2
ANXA13
SI
OPTN
AFP
AGR3
VCAN
SEMA3A
HEPACAM2
CADPS
I
G2M phase
S phase
BEST4 entero prog
Goblet prog
Enterocyte
entero-endocrine
Early enterocyte1/2
LGR5 stem
J
PCNA
CDK1
LGR5
ASCL2
SI
ANPEP
BEST4
CHGA
CLCA1
FCGBP
expression
latent time
K
Fetal epithelium differentiation pseudo-time
Relative exp.
1
0
SMC4
LDHA
GPM6B
DEPDC1B
GLIPR2
RGS10
CDK1
ATAD2
H2AFX
MYC
MT1E
BTG2
GPR82
NTN4
CDH2
NEK2
TPD52L1
PRSS2
PCSK9
CKAP5
CCNA2
CCDC14
HOTAIRM1
GOLIM4
ASGR2
RALYL
GSTA2
SCD5
HIF3A
AQP3
SST
SLC35G1
TDO2
ALB
SLC28A1
KEL
ACE2
RARRES1
CDKN2B-AS1
HSD17B11
SLC2A2
ETV1
DNAJC12
CACNA2D1
BTNL8
CHST9
MEIS2
MEIS1
FOSB
KCNH3

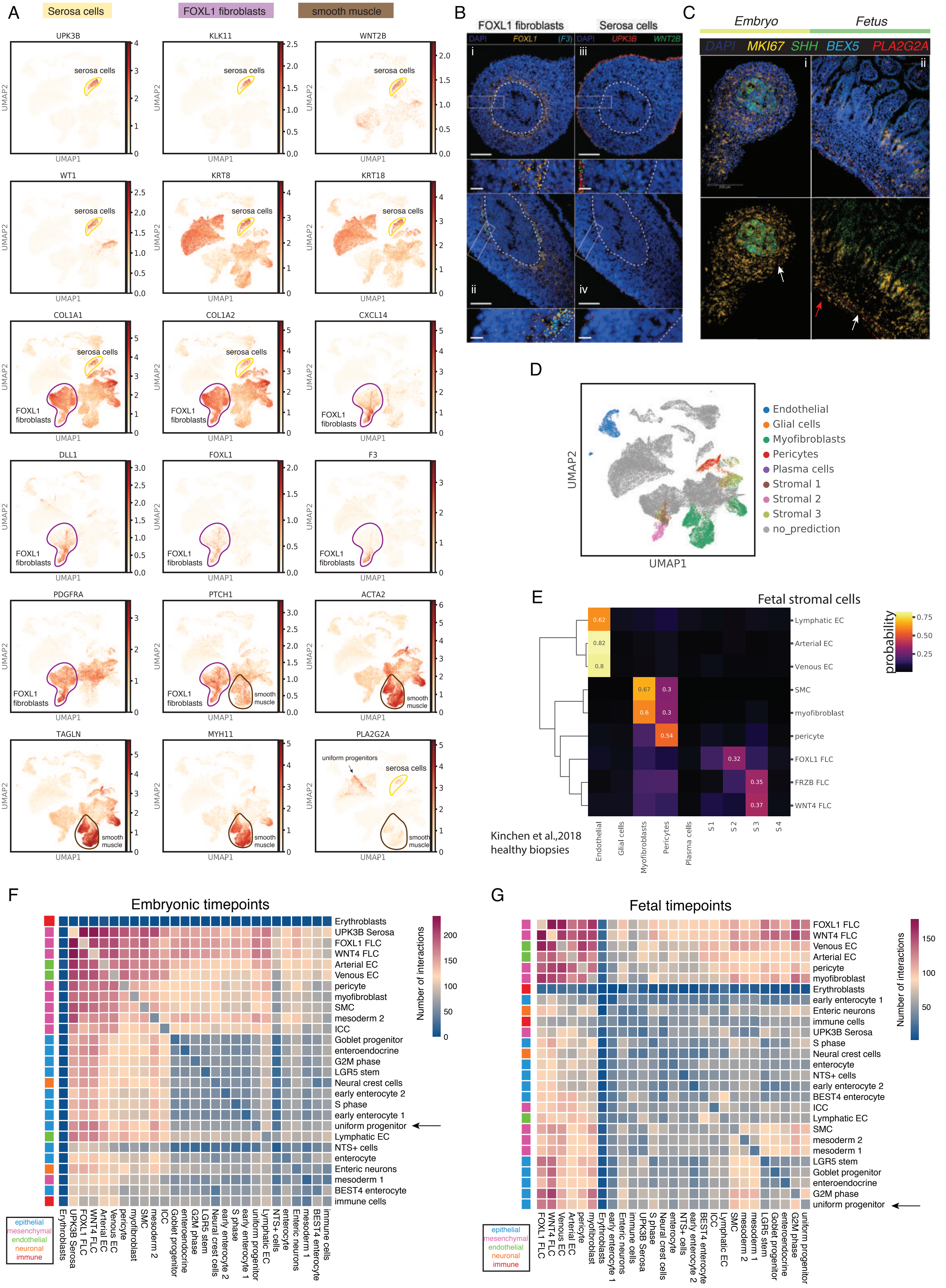
A
Serosa cells
FOXL1 fibroblasts
smooth muscle
UPK3B
KLK11
WNT2B
WT1
KRT8
KRT18
COL1A1
COL1A2
CXCL14
DLL1
FOXL1
F3
PDGFRA
PTCH1
ACTA2
TAGLN
MYH11
PLA2G2A
serosa cells
FOXL1 fibroblasts
smooth muscle
uniform progenitors
UMAP1
UMAP2
B
FOXL1 fibroblasts
Serosa cells
DAPI FOXL1 (F3)
DAPI UPK3B WNT2B
C
Embryo
Fetus
DAPI MKI67 SHH BEX5 PLA2G2A
D
Endothelial
Glial cells
Myofibroblasts
Pericytes
Plasma cells
Stromal 1
Stromal 2
Stromal 3
no_prediction
E
Fetal stromal cells
probability
Lymphatic EC
Arterial EC
Venous EC
SMC
myofibroblast
pericyte
FOXL1 FLC
FRZB FLC
WNT4 FLC
Endothelial
Glial cells
Myofibroblasts
Pericytes
Plasma cells
S1
S2
S3
S4
Kinchen et al.,2018 healthy biopsies
F
Embryonic timepoints
Number of interactions
Erythroblasts
UPK3B Serosa
FOXL1 FLC
WNT4 FLC
Arterial EC
Venous EC
pericyte
myofibroblast
SMC
mesoderm 2
ICC
Goblet progenitor
enteroendocrine
G2M phase
LGR5 stem
Neural crest cells
early enterocyte 2
S phase
early enterocyte 1
uniform progenitor
Lymphatic EC
NTS+ cells
enterocyte
Enteric neurons
mesoderm 1
BEST4 enterocyte
immune cells
epithelial
mesenchymal
endothelial
neuronal
immune
G
Fetal timepoints
Number of interactions
FOXL1 FLC
WNT4 FLC
Venous EC
Arterial EC
pericyte
myofibroblast
Erythroblasts
early enterocyte 1
Enteric neurons
immune cells
UPK3B Serosa
S phase
Neural crest cells
enterocyte
NTS+ cells
early enterocyte 2
BEST4 enterocyte
ICC
Lymphatic EC
SMC
mesoderm 2
mesoderm 1
LGR5 stem
Goblet progenitor
enteroendocrine
G2M phase
uniform progenitor
epithelial
mesenchymal
endothelial
neuronal
immune

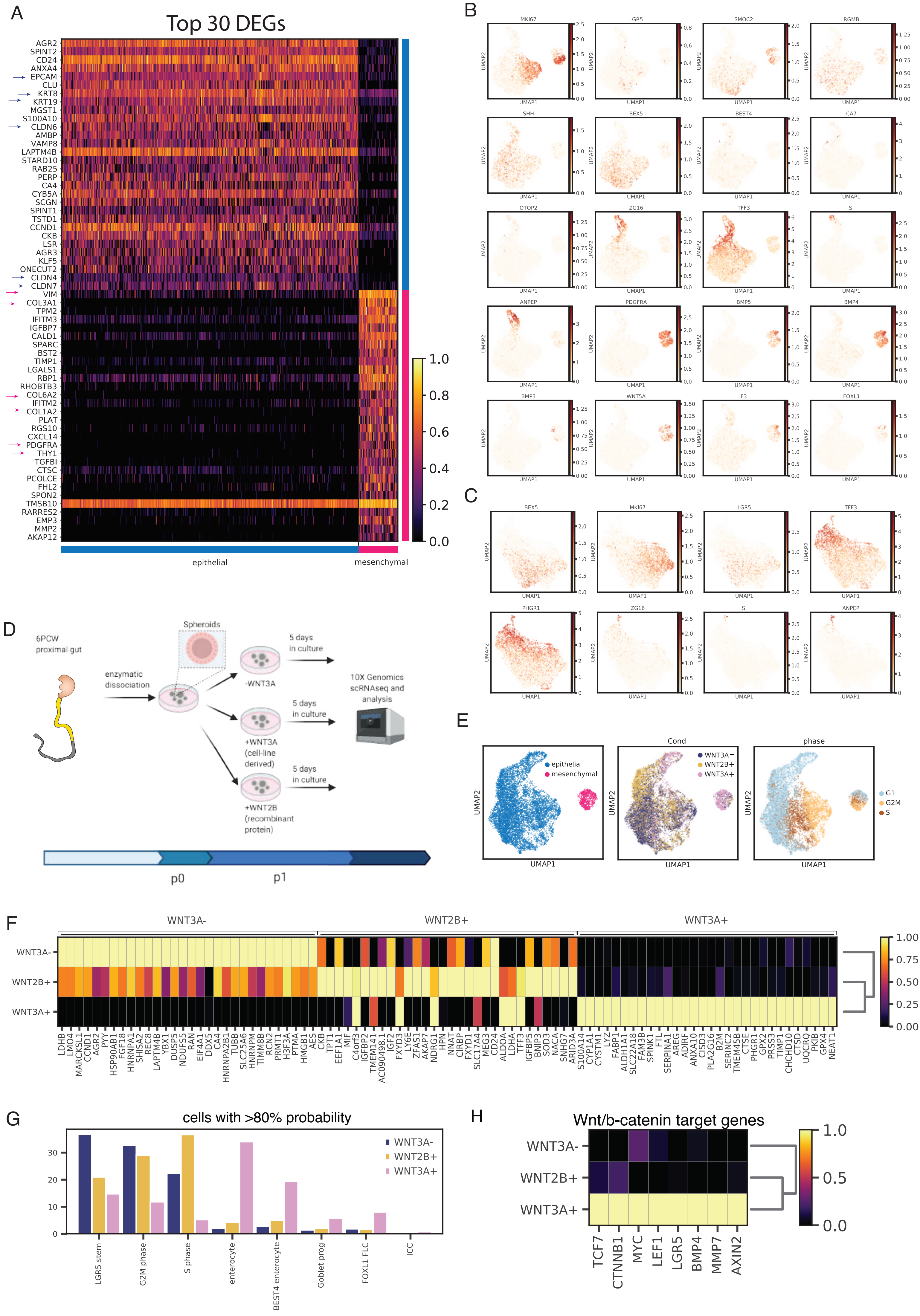
A
Top 30 DEGs
epithelial
mesenchymal
B
C
D
6PCW proximal gut
enzymatic dissociation
Spheroids
5 days in culture
-WNT3A
+WNT3A (cell-line derived)
+WNT2B (recombinant protein)
10X Genomics scRNAseq and analysis
p0
p1
E
epithelial
mesenchymal
Cond
WNT3A-
WNT2B+
WNT3A+
phase
G1
G2M
S
UMAP1
UMAP2
F
WNT3A-
WNT2B+
WNT3A+
G
cells with >80% probability
LGR5 stem
G2M phase
S phase
enterocyte
BEST4 enterocyte
Goblet prog
FOXL1 FLC
ICC
H
Wnt/b-catenin target genes
TCF7
CTNNB1
MYC
LEF1
LGR5
BMP4
MMP7
AXIN2

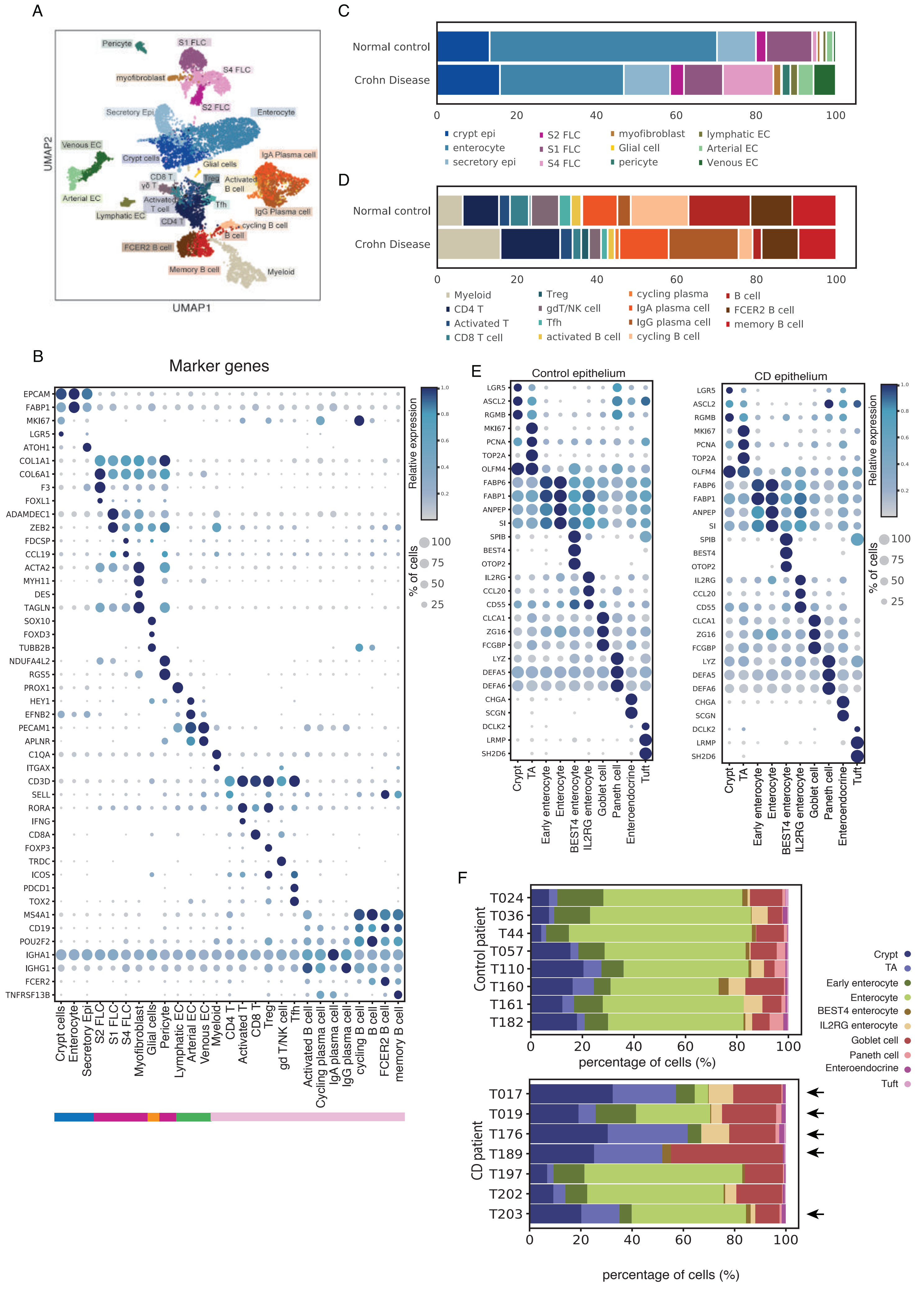
A
B
C
D
E
F
UMAP1
UMAP2
Pericyte
S1 FLC
S4 FLC
myofibroblast
S2 FLC
Secretory Epi
Enterocyte
Venous EC
Crypt cells
Glial cells
IgA Plasma cell
Arterial EC
Lymphatic EC
Tfh
IgG Plasma cell
cycling B cell
FCER2 B cell
Memory B cell
Myeloid
Normal control
Crohn Disease
crypt epi
enterocyte
secretory epi
S2 FLC
S1 FLC
S4 FLC
myofibroblast
Glial cell
pericyte
lymphatic EC
Arterial EC
Venous EC
Myeloid
CD4 T
Activated T
CD8 T cell
Treg
gdT/NK cell
Tfh
activated B cell
cycling plasma
IgA plasma cell
IgG plasma cell
cycling B cell
B cell
FCER2 B cell
memory B cell
Marker genes
Relative expression
% of cells
Control epithelium
CD epithelium
Control patient
CD patient
percentage of cells (%)
Crypt
TA
Early enterocyte
Enterocyte
BEST4 enterocyte
IL2RG enterocyte
Goblet cell
Paneth cell
Enteroendocrine
Tuft
T024
T036
T44
T057
T110
T160
T161
T182
T017
T019
T176
T189
T197
T202
T203

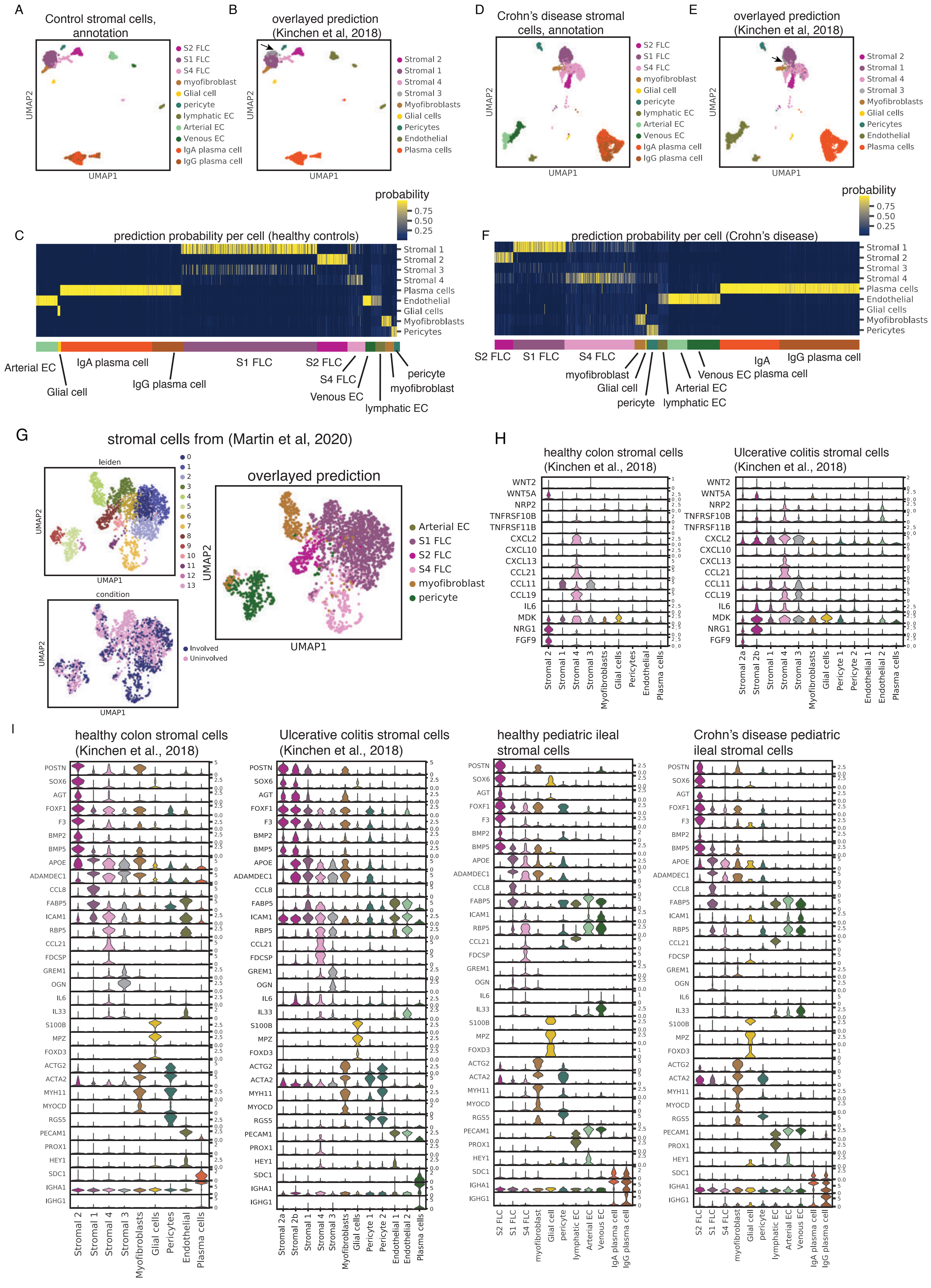
A
Control stromal cells, annotation
S2 FLC
S1 FLC
S4 FLC
myofibroblast
Glial cell
pericyte
lymphatic EC
Arterial EC
Venous EC
IgA plasma cell
IgG plasma cell
UMAP1
UMAP2
B
overlayed prediction (Kinchen et al, 2018)
Stromal 2
Stromal 1
Stromal 4
Stromal 3
Myofibroblasts
Glial cells
Pericytes
Endothelial
Plasma cells
C
prediction probability per cell (healthy controls)
probability
0.75
0.50
0.25
Stromal 1
Stromal 2
Stromal 3
Stromal 4
Plasma cells
Endothelial
Glial cells
Myofibroblasts
Pericytes
Arterial EC
IgA plasma cell
IgG plasma cell
S1 FLC
S2 FLC
S4 FLC
Venous EC
lymphatic EC
pericyte
myofibroblast
Glial cell
D
Crohn's disease stromal cells, annotation
E
overlayed prediction (Kinchen et al, 2018)
F
prediction probability per cell (Crohn's disease)
IgA plasma cell
IgG plasma cell
G
stromal cells from (Martin et al, 2020)
leiden
0
1
2
3
4
5
6
7
8
9
10
11
12
13
condition
Involved
Uninvolved
overlayed prediction
H
healthy colon stromal cells (Kinchen et al., 2018)
Ulcerative colitis stromal cells (Kinchen et al., 2018)
WNT2
WNT5A
NRP2
TNFRSF10B
TNFRSF11B
CXCL2
CXCL10
CXCL13
CCL21
CCL11
CCL19
IL6
MDK
NRG1
FGF9
Stromal 2a
Stromal 2b
Pericyte 1
Pericyte 2
Endothelial 1
Endothelial 2
I
healthy colon stromal cells (Kinchen et al., 2018)
Ulcerative colitis stromal cells (Kinchen et al., 2018)
healthy pediatric ileal stromal cells
Crohn's disease pediatric ileal stromal cells
POSTN
SOX6
AGT
FOXF1
F3
BMP2
BMP5
APOE
ADAMDEC1
CCL8
FABP5
ICAM1
RBP5
CCL21
FDCSP
GREM1
OGN
IL6
IL33
S100B
MPZ
FOXD3
ACTG2
ACTA2
MYH11
MYOCD
RGS5
PECAM1
PROX1
HEY1
SDC1
IGHA1
IGHG1

Figure S1. Related to Figures 1 and 2. Quality control of the three datasets. A) and B) Bar plots with a number of droplets after QC and Scrublet doublet exclusion grouped by cell type group. Scatter plots show the number of genes over the number of counts per cell, where each dot is a cell. Sample contribution to each cluster in C) Control and D) CD datasets. UMAP plots colored by sample age group in E) Control and F) CD samples. G) Bar plots with number of cells in fetal dataset and UMAP with sample contribution to each cluster. H) Number of fetal cells captured in each timepoint colored by enrichment strategy (top) and region (bottom). I) Number of cells of broad epithelial cell clusters (Uniform progenitor, Small Intestine Epi, Colonic Epi, Secretory Epi) in each of three developing anatomical regions (duo-jejunum, ileum and colon). Epi = epithelium. For fetal samples, the average cell recovery was 1,800 with a total of 62,849 cells at a mean depth of 13, 570 reads per cell and 3,027 mean genes per cell. For paediatric samples, the average recovery was 1,400 cells with 8,093 reads per cell and 1,859 mean genes per cell. CD = Crohn's disease. Fetal sample ages in post-conception weeks were as follows: BRC2029 - 6.1, BRC2026 - 8.4, BRC2043 - 10.2, BRC2046 - 6.7, BRC2049- 6.9, BRC2121 - 9.2, BRC2119- 7.9, BRC2133 - 9.9, BRC2134- 10. Patient ages were as follows: Control group (T036- 4 years, T110- 4 years, T161- 4 years, T057- 6 years, T182- 9 years, T44- 10 years, T024- 12 years, T160- 10 years);

Figure S2. Related to Figure 3. Marker genes and trajectory analysis of embryonic and fetal intestinal epithelium. A) UMAP projection of cells after cell-cycle gene exclusion from gene list and dimensionality reduction. The cells are coloured by their cell type annotation. B) Gene expression of adult-like LGR5 stem cell genes. C) Feature plots with selected differentially expressed genes in uniform progenitors. prog = progenitors. PAGA graphs (D and E) and velocity-driven paga graphs (F and I) for embryonic and fetal epithelial cells, respectively. prog = progenitor. PAGA graphs can illustrate either connectivities (solid/dashed lines) or transitions (arrows), where the latter are defined by scVelo. G) and J) Differentiating cell type marker gene expression along the pseudotime (latent time). Cells are colored by the cell type as in UMAP. *PCNA*, *CDK1*- S and G2M phase cells; *BEX5*- uniform progenitors; *LGR5*, *ASCL2*- LGR5 stem cells; *SI*, *ANPEP* - enterocytes; *CHGA*- enteroendocrine cells; *NTS*- NTS+ cells; *BEST4*- BEST4 enterocytes; *CLACA1*, *FCGB*P- goblet cells. H) and K) Top 50 driver genes that display pronounced dynamic behaviour along the pseudotime in embryonic and fetal epithelium, respectively. Top bars in H) and K) represent cell annotation and their colors match annotation colors in F) and I), respectively. prog= progenitor.

Figure S3. Related to Figure 4. Cell-cell interactions in embryonic and fetal gut. A) Feature plots with selected genes expressed in three stromal populations in embryonic and fetal gut. Circled populations are serosal cells (yellow), FOXL1 fibroblasts (purple), smooth muscle cells (brown). Arrow points to uniform progenitors. B) smFISH images of serosa mesothelial cells and FOXL1 fibroblasts. i and ii in B) are two biological replicates imaging location of FOXL1 fibroblasts using DAPI, *FOXL1*, and *F3* (stained only in ii), while iii and iv are two biological replicates for locating Serosa mesothelial cells using co-expression of DAPI, *UPK3B*, and *WNT2B*. Scale bar main panel = 100 µm, zoom panel = 50 µm. C) smFISH images of cycling cells (*MKI67*), epithelial cells (*SHH* and *BEX5*), and *PLA2G2A* gene that is expressed in multiple populations in the gut. White arrows point to *PLA2G2A*-expressing SMCs, red arrow points to *PLA2G2A*-expressing UPK3B Serosa cells. Scale bar main panel = 100 µm, zoom panel = 50 µm. D) UMAP with overlaid predictions of fetal cells. Predictions were made using logistic regression models trained on adult stromal cells from (Kinchen *et al.*, 2018). E) Heatmap with mean prediction probability in stromal cell clusters. Heatmaps with number of interactions in embryonic (F) and fetal (G) samples as quantified using CellphoneDB v2.0. Color legend indicates the cell type lineage (epithelial-blue, mesenchymal-pink, endothelial-green, neuronal-orange, immune/erythroblast-red) of each plotted cell type. Arrows point to cell-types discussed in text. FLC = fibroblasts, EC = endothelial cells, ICC = interstitial cells of Cajal, S= stromal, SMC = smooth muscle cells.

Figure S4. Related to Figure 5. Marker gene expression in fetal intestinal organoids and fetal intestinal organoid co-culture with recombinant WNT2B. A) Heatmap of top 30 differentially expressed genes (DEGs) between mesenchymal and epithelial cells captured in the intestinal organoid culture. The arrows point to key genes associated with either mesenchymal (pink) or epithelial (blue) identity. B) Feature plot with mesenchymal and epithelial marker gene expression in WNT3A- and WNT3A+ organoids. C) Feature plot of epithelial marker genes expressed in p1 and p17 organoid cells. *MKI67*- cycling cells; *LGR5, SMOC2, RGM*B- LGR5 stem cells; *SHH, BEX5*- uniform progenitors; *BEST4, CA7, OTOP2*- BEST4 enterocytes; *ZG16, TFF3*- goblet cells; *SI, ANPEP, PHGR1* - enterocytes; *PDGFRA, BMP3, BMP4, BMP5, WNT5A, F3, FOXL1*- FOXL1 fibroblasts; D) Schematic with experimental strategy for organoid culture comparison between WNT2B (recombinant protein) and WNT3A (cell-line derived). The organoids were generated from dissociated cells of embryonic proximal ileum, cultured in three different culture conditions and sequenced on the day 5 of passage 1. E) UMAP visualisation of integrated organoid cells from three conditions (WNT3A- or no WNT, WNT2B+, WNT3A+). The UMAPs are colored by cluster identity (left), culture condition (middle), or cell cycle phase score (right). F) Top 30 differentially expressed genes in the epithelial organoid cells between three culture conditions. G) abundance of cells as predicted with logistic regression with more than 80% probability of matching transcriptionally. H) Relative expression of key canonical WNT pathway genes in the epithelium of three culture conditions. PCW = post-conception weeks.

Figure S5. Related to Figure 6. Crohn's disease patient dataset. A) UMAP projection of CD samples colored by cell type annotation. B) Dot plot with relative expression and percentage of cells expressing marker genes in paediatric CD dataset. C) and D) Bar plots with percentage of cells in CD dataset as compared with healthy paediatric (Control) dataset, grouped by the broad cell group (stromal =epithelial, mesenchymal, endothelial and glial, and immune = myeloid, T cells and B cells). E) Dot plots with relative expression and percentage of cells expressing marker genes in paediatric healthy (left) and CD (right) epithelium. F) Barplots show abundance of epithelial cell subsets in healthy controls (top, n=8) and children with CD (bottom, n=7). Arrows point to samples that were grouped as "inflamed Crohn's disease". CD = Crohn's disease.

Figure S6. Related to Figure 6. Pediatric data comparisons with published single-cell datasets. UMAP projections of stromal cells from healthy pediatric dataset colored by A) original cell type annotation defined in this study and B) overlaid predicted annotation generated by using a logistic regression model trained on scRNAseq data from Kinchen et al. 2018. arrows point to cells predicted to be Stromal 3 cell type. The prediction probabilities for each cell are shown in heatmap C). X-axis shows predicted cell type labels as in Kinchen et al. (2018) and y-axis is grouped by the original annotation as in A). The same logistic regression analysis was done for CD samples and results are shown in UMAPs with C) original annotation, D) overlaid predicted annotations (arrows point to cells predicted to be Stromal 3 cell type), and probability heatmap F). G) The comparison of ileal stromal cells from (Martin *et al.*, 2019) UMAP plots show ileal stromal cells from (Martin *et al.*, 2019).
